# Single-Cell Expression Variability Implies Cell Function

**DOI:** 10.3390/cells9010014

**Published:** 2019-12-19

**Authors:** Daniel Osorio, Xue Yu, Yan Zhong, Guanxun Li, Erchin Serpedin, Jianhua Z. Huang, James J. Cai

**Affiliations:** 1 Department of Veterinary Integrative Biosciences, Texas A&M University, College Station, TX 77843, USA; dcosorioh@tamu.edu; 2 Department of Veterinary Pathobiology, Texas A&M University, College Station, TX 77843, USA; myu@cvm.tamu.edu; 3 Department of Statistics, Texas A&M University, College Station, TX 77843, USA; yanzhong@stat.tamu.edu (Y.Z.); victorxun@tamu.edu (G.L.); 4 Department of Electrical and Computer Engineering, Texas A&M University, College Station, TX 77843, USA; eserpedin@tamu.edu; 5 Interdisciplinary Program of Genetics, Texas A&M University, College Station, TX 77843, USA

**Keywords:** single-cell RNA sequencing, scRNA-seq, single-cell expression variability, cell-to-cell variation, lymphoblastoid cell line, airway epithelial cell, dermal fibroblast, induced pluripotent stem cell

## Abstract

As single-cell RNA sequencing (scRNA-seq) data becomes widely available, cell-to-cell variability in gene expression, or *single-cell expression variability* (scEV), has been increasingly appreciated. However, it remains unclear whether this variability is functionally important and, if so, what are its implications for multi-cellular organisms. Here, we analyzed multiple scRNA-seq data sets from lymphoblastoid cell lines (LCLs), lung airway epithelial cells (LAECs), and dermal fibroblasts (DFs) and, for each cell type, selected a group of homogenous cells with highly similar expression profiles. We estimated the scEV levels for genes after correcting the mean-variance dependency in that data and identified 465, 466, and 364 highly variable genes (HVGs) in LCLs, LAECs, and DFs, respectively. Functions of these HVGs were found to be enriched with those biological processes precisely relevant to the corresponding cell type’s function, from which the scRNA-seq data used to identify HVGs were generated—e.g., cytokine signaling pathways were enriched in HVGs identified in LCLs, collagen formation in LAECs, and keratinization in DFs. We repeated the same analysis with scRNA-seq data from induced pluripotent stem cells (iPSCs) and identified only 79 HVGs with no statistically significant enriched functions; the overall scEV in iPSCs was of negligible magnitude. Our results support the “variation is function” hypothesis, arguing that scEV is required for cell type-specific, higher-level system function. Thus, quantifying and characterizing scEV are of importance for our understating of normal and pathological cellular processes.

## 1. Introduction

Cells are fundamental units of cellular function. Cells in multi-cellular organisms can be organized into groups, or cell types, based on shared features that are quantifiable. A multicellular organism is usually composed of cells of many different types—each is a distinct functional entity differing from the other. Within the same cell type, cells are nearly identical and are considered to carry the same cell function or biological processes associated with the cell type that ensures the homeostatic state of the organism where the cell is present.

The recent development of single-cell RNA sequencing (scRNA-seq) technologies has brought the increasingly high-resolution measurements of gene expression in single cells [1]. This power has been widely adopted to refine the categories of known cell types and analyze complex tissues systematically and reproducibly [2]. The power of scRNA-seq has also been harnessed to identify novel cellular states among the same type of cells [3]. 

Cells of the same type and at the same state may still show marked intrinsic cell-to-cell variability in gene expression or single cell expression variability (scEV), even under the same environmental conditions [4,5,6]. The importance of this intrinsic variability is increasingly appreciated [7,8]. Changes in the magnitude of scEV have been associated with development [9,10,11,12], aging [13,14], and pathological processes [15,16].

Dueck and colleagues [17] put forward the so-called “variation is function” hypothesis, saying that scEV per se might be crucial for population-level function. They used the term “single cell variation or variability” to refer to diversity within an ensemble that has been previously defined as being generally homogeneous, rather than diversity of cell types that are clearly distinct and already recognized. The main focus of their question is to ask how the individual cells with different gene expression levels may interact to causally generate higher-level function. If the hypothesis turns out to be true, it means that the intrinsic cell-to-cell variability is an indicator of a diversity of hidden functional capacities, which facilitate the collective behavior of cells. This collective behavior is essential for the function and normal development of cells and tissues [18,19]. The loss of this collective cellular behavior may result in disease. Thus, investigation of the intrinsic cell-to-cell variability may contribute to the understanding of pathological processes associated with disease development.

It is worth noting that the level of intrinsic cell-to-cell variability needs to be measured within a highly homogeneous population of cells. This is because many micro-environmental perturbations and stochastic factors at the cellular level are known to change the scEV. These factors may include local cell density, cell size, shape and rate of proliferation, cell cycle [20,21,22,23,24]. To work on the cell-to-cell variability, these confounding factors have to be controlled.

Exponential scaling of scRNA-seq has made it feasible to study scEV across thousands of cells [25] and quantify scEV based on measures of statistical dispersion such as the coefficient of variation (CV) [26,27]. The sheer number of cells sequenced in a “typical” droplet-based scRNA-seq experiment allows us to filter out for a sizable number of highly homogeneous cells, based on the similarity between their global transcriptional profiles. With these selected core of highly similar cells, we are able to test the “variation is function” hypothesis. Furthermore, using established statistical methods, we are able to control for many sources of technical variation that may confound the measurement of scEV to obtain an unbiased estimate. For instance, single-molecule capture efficiency, 3’ end bias due to single-cell RNA library preparation protocol, and low expression of genes are examples of known sources of technical variation [28], which should be controlled for using statistical means.

The characterization of the impact of scEV on cell function requires the understanding of which genes show greater or less cell-to-cell variability in their expression. These feature genes may carry valuable information that can facilitate the elucidation of underlying regulatory networks [29]. Once these genes are identified, a follow-up question is whether they are tissue- or cell type-specific—i.e., whether the same genes will be identified for different tissues or cell types. Our working hypothesis is in line with the “variation is function” hypothesis, that is, different tissues or cell types have different sets of highly variable genes (HVGs), and these HVGs should be enriched with functions that reflect the biological processes associated with the respective tissues or the cell types. To test this, we analyzed three scRNA-seq data sets generated for three different cell types. Each data set contains thousands of cells. For each cell type, we selected a highly homogenous population of cells, with the help of a newly developed dimensionality reduction method, called potential of heat-diffusion for affinity-based trajectory embedding (PHATE) [30]. We estimated scEV among the selected cells for each of these cell types and further systematically characterized functions of identified HVGs. We show that HVGs are highly specific to cell types, i.e., different cell types have different sets of HVGs; functions of HVGs precisely mirror the biological processes of the corresponding cell types.

## 2. Materials and Methods

### 2.1. LCL Cell Culture and scRNA-seq Experiment

The lymphoblastoid cell line (LCL) GM12878 was purchased from the Coriell Institute for Medical Research. They were cultured in the RPMI-1640 medium supplied with 2 mM L-glutamine and 20% of non-inactivated fetal bovine serum, incubated at 37 °C under 5% CO_2_ atmosphere. For maintenance, cells were subcultured every three days by adding fresh medium. For single-cell sequencing, each cell line was subcultured with 200,000 viable cells/mL. Cells were harvested for single-cell sample preparation and sequencing on day four (stationary phase) following the sample preparation demonstrated protocol and Single Cell 3’ Reagent Kits v2 user guide provided by 10× Genomics. Briefly, cells were mixed well in each flask, and 1 mL of cell suspensions from each cell line were taken out. The cells were washed three times by centrifuging, suspending, and resuspending in 1× PBS with 0.04% BSA. Viable cells were then counted using an automated cell counter (Thermo Fisher Scientific, Carlsbad, CA, USA). Cells (~5000 per cell line) were then pelleted and resuspended in the nuclease-free water based on the cell suspension volume calculator table, followed by GEM (gel bead-in-emulsions) generation and barcoding, the post-GEM-RT cleanup, cDNA amplification, and library construction and sequencing. The experiments were conducted at the Texas A&M Institute for Genome Sciences and Society. The sequencing was conducted in the North Texas Genome Center facilities using a Novaseq 6000 sequencer (Illumina, San Diego, CA, USA). Raw reads for each cell were analyzed using Cell Ranger (v2.0.0, 10× Genomics, Pleasanton, CA, USA) and the outputs were aligned to the human reference genome (GRCh38) to obtain the counts [31].

### 2.2. Non-LCL scRNA-seq Data Sets

The scRNA-seq data for lung airway epithelial cells (LAECs) was downloaded from the GEO database using accession number GSE115982. The original data was generated in the study of [32] for CCR10^−^ and CCR10^+^ LAECs. We used the data generated from the CCR10^−^ cells with the sample identifier GSM3204305. The scRNA-seq data for primary dermal fibroblasts (DFs) was generated in the study of [33]. We downloaded the data for unstimulated DFs from the ArrayExpress database using accession number E-MTAB-5988. To support our findings, we processed additional samples and compared the results obtained for the same cell type. We selected the transcriptomic profile of fibroblasts (7052 and 6503 cells) from samples extracted from two different lung regions (GEO accessions: GSM2894834 and GSM2894835); and for the iPSC sample, we used another iPSC culture (9146 cells) generated in the study of [34], and downloaded from the ArrayExpress database using accession number E-MTAB-6268. All of these data sets were produced using 10× Genomics scRNA-seq solutions.

### 2.3. Selection of Highly Homogeneous Populations of Cells

We used a supervised data analysis method to select highly homogeneous cells based on the scRNA-seq expression profile of each cell. The procedure is summarized in a flowchart (Supplementary Figure S1). The main steps are as follows. We used Seurat (v2.2.0) [35] to assign each cell into a cell cycle phase and excluded cells that were not considered to be in G1-phase. We removed genes encoded in the mitochondrial genome from the analysis. We then selected and retained cells with a library size between 50 and 95 percentiles. We used PHATE [30] to generate a embedding plot of all remaining cells and inspected the distributions of cells in the three-dimensional plot and manually picked one “core” cell. Finally, an additional 999 cells that were closest to the core cell, according to the Euclidean distances between cells, were selected to form the final 1000-cell population. This selection procedure was applied to each of the three cell types independently, as well as for the additional samples used to support our findings.

### 2.4. Identification of HVGs

Identification of highly variable genes (HVGs) was based on the assumption that high expression variability of these genes across cells relative to their mean expression is caused by biological effects rather than merely technical noise. We used the method proposed in [36], which is implemented in function sc_hvg of the scGEAToolbox package (https://github.com/jamesjcai/scGEAToolbox) [37]. This method starts by adjusting the library size and assumes that the observed mean expression (${\hat{\mu }}_{i}$) and the observed CV^2^ (${\hat{w}}_{i}$) of gene i among cells have the following relationship:

(1) $$E\left({\hat{w}}_{i}\right)\approx a_{1}/{\hat{\mu }}_{i}+a_{0}$$

And

(2) $$\frac{{\hat{w}}_{i}}{a_{1}/{\hat{\mu }}_{i}+a_{0}}∼\chi_{m-1}^{2}/\left(m-1\right)$$

where m is the number of cells. The values of $a_{0}$ and $a_{1}$ are estimated by generalized linear regression (GLM). The residual term ${\hat{w}}_{i}/\left({\hat{a}}_{1}/{\hat{\mu }}_{i}+{\hat{a}}_{0}\right)$ for each gene is used to test if the observed CV^2^ is significantly larger than the expected CV^2^ via a chi-squared test. Multiple testing *p*-value adjustments were performed by controlling FDR [38].

### 2.5. Function Enrichment Analyses

To identify the overrepresented biological functions of HVGs in different cell types, we performed the GO enrichment analysis using Enrichr [39,40] and GOrilla [41]. Enrichr was conducted for HVGs (FDR <0.01) against the rest of the expressed genes with respect to pathways collected in the Reactome pathway knowledgebase [42]. GOrilla was performed with the list of genes sorted in descending order of their residual variability.

### 2.6. Analyses of co-Expression Network and Regulatory Regions of HVGs

MAGIC [43] was used to impute the expression matrix. The co-expression networks were constructed using 1-correlation as a distance measure, using SBEToolbox [44]. The motif analysis of the regulatory regions associated with the HVGs was performed using the GREAT [45]. Genomic coordinates for the HVG genes from the Human Reference Genome (hg19) were downloaded from the Ensembl Biomart [46] and converted into bed format using an in-house script. Identified motifs were searched against the JASPAR database [47] to match the binding sites of corresponding TFs.

### 2.7. Data Availability

The data sets used in this study and computer code are available.

1. LCLs GM12878 scRNA-seq data in the GEO database with accession number GSE126321: https://www.ncbi.nlm.nih.gov/geo/query/acc.cgi?acc=GSE126321
2. LAEC scRNA-seq data in the GEO database with accession number GSE115982: https://www.ncbi.nlm.nih.gov/geo/query/acc.cgi?acc=GSE115982
3. DF scRNA-seq data in ArrayExpress database with accession number E-MTAB-5988: https://www.ebi.ac.uk/arrayexpress/experiments/E-MTAB-5988
4. Human iPSC scRNA-seq data in ArrayExpress database with accession number E-MTAB-6687: https://www.ebi.ac.uk/arrayexpress/experiments/E-MTAB-6687
5. Fibroblasts scRNA-seq data in GEO database with accession number GSM2894834: https://www.ncbi.nlm.nih.gov/geo/query/acc.cgi?acc=GSM2894834
6. Fibroblasts scRNA-seq data in GEO database with accession number GSM2894835: https://www.ncbi.nlm.nih.gov/geo/query/acc.cgi?acc=GSM2894835
7. Human iPSC scRNA-seq data in ArrayExpress database with accession number E-MTAB-6268: https://www.ebi.ac.uk/arrayexpress/experiments/E-MTAB-6268/
8. Computer codes used to analyze data: https://github.com/cailab-tamu/HVG

## 3. Results

### 3.1. Single-Cell RNA Sequencing and Selection of Highly Homogenous Cells

In this study, we experimented with the transcriptomic profiles of three different human cell types, namely, lymphoblastoid cell line (LCL), lung airway epithelial cell (LAEC), and dermal fibroblast (DF). We estimated single-cell expression variability (scEV) for each of these cell types, individually.

To obtain the scRNA-seq data for LCL, we cultured GM12878, an LCL strain widely used in genomic research, prepared cells using a 10× Genomics Chromium Controller, and sequenced a total of 7045 cells [31]. This data has been deposited in the NCBI Gene Expression Omnibus (GEO) database (accession number GSE126321). For the other two cell types, LAEC and DF, we obtained the scRNA-seq data for 3863 and 2553 cells from the studies of [32] and [33], respectively. In addition, we also processed two more samples for fibroblasts and one for iPSC cells (see Section 2.7. for data availability) to cross-validate our findings. All scRNA-seq data sets of the three cell types and the additional samples used were produced using 10× Genomics droplet-based solution and made use of unique molecular identifiers (UMIs) [48].

**Figure 1:**
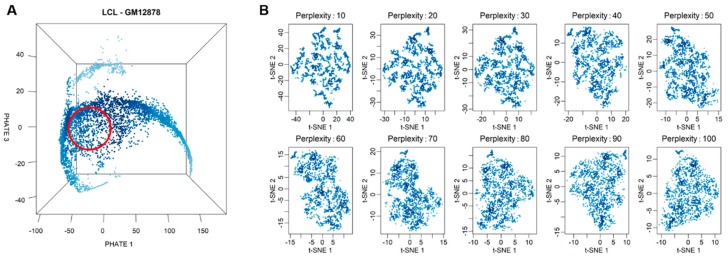
Selection of a highly homogenous cell population for variability analysis. (**A**) Three-dimensional PHATE embedding plot for G1-phase cells of GM12878. Each point represents a single cell in the three-dimensional space. The red circle indicates the approximate positions of 1000 selected cells. (**B**) Embedding plots generated for the 1000 selected cells with a t-SNE algorithm with a series of perplexity values.

For each cell type, we employed a data analysis procedure, a filter pipeline on scRNA-seq data, to select highly similar populations of cells (see Section 2 for materials and methods). These selected cells are a representative population of each the cell type. The main steps of the filter pipeline are depicted in Supplementary Figure S1. Briefly, we first excluded mitochondrial DNA-encoded genes from the analysis. We then excluded cells in the S- or G2/M phases and only retained G1-phase cells. We also excluded cells with library size <55 percentile or >99 percentile. Finally, we used PHATE to produce the low-dimensionality representation of the remaining cells to inspect between-cell structure driven by heterogeneity in gene expression. PHATE is a visualization method that captures both local and global nonlinear structure in data by an information-geometry distance between data points [30]. As seen from the PHATE projection (Figure 1A), several “arms” of cells show the structure of the cell-to-cell relationship. Based on the observation, we manually picked one “core” cell at the root of the arms of cells in the middle of the cell cloud (red circle in Figure 1A). The core cell and 999 nearest cells around it were then selected using the *k*-nearest neighbors algorithm to form the final population of 1000 cells, which was used for subsequent data analyses. 

To examine the homogeneity of selected cells, we used t-distributed stochastic neighbor embedding (t-SNE) [49] to position all 1000 selected cells in the two-dimensional t-SNE space. Compared to PHATE, t-SNE is a more commonly used nonlinear visualization algorithm for revealing structures in high-dimensional data, emphasizing local neighborhood structure within the data. When running t-SNE, we experimented with a series of perplexity values to produce multiple plots for the same population of selected cells. t-SNE is known to be sensitive to hyperparameters [50]. In general, when different parameter values are given, t-SNE tends to produce different cell clustering plots. However, for our selected cells, no structure is observed in any of these t-SNE embedding plots (Figure 1B). The same results were obtained for the other two cell types as well as using the uniform manifold approximation and projection (UMAP) as an alternative embedding algorithm [50] (Supplementary Figure S2). Thus, we confirm that cells selected with our filter pipeline are highly homogenous populations of representative cells for each cell type.

### 3.2. Identification of Highly Variable Genes

Highly variable genes (HVGs) are expressed variably across homogeneous cells of the same type. For each cell type, we used the method of [36] to identify HVGs from scRNA-seq data of the homogeneous population of selected cells. In this method, the relationship between the squared coefficient of variation (CV^2^) of genes and their average expression (μ) is considered. The relationship between log-transformed CV^2^ and log-transformed μ is fitted with a generalized linear model (GLM) by using a gamma distribution, and the expected CV^2^ for a given μ is calculated with the fitted curve. The log-transformed ratio between observed CV^2^ and expected CV^2^ [=log(observed CV^2^) − log(expected CV^2^], called “residual variability”, is used as the measurement of scEV. Since the expected CV^2^ captures the variability originated from technical noise, the residual variability is considered to be an unbiased measure of biological variability. Indeed, after the correction, the μ-CV^2^ correlation disappeared (Supplementary Figure S3). We repeated the procedure for correcting μ-CV dependency using another method [51] and obtained the qualitatively similar results in terms of identified HVGs and enriched functions (Supplementary Figure S4). Here, we only report the results obtained using the method of [36].

**Figure 2:**
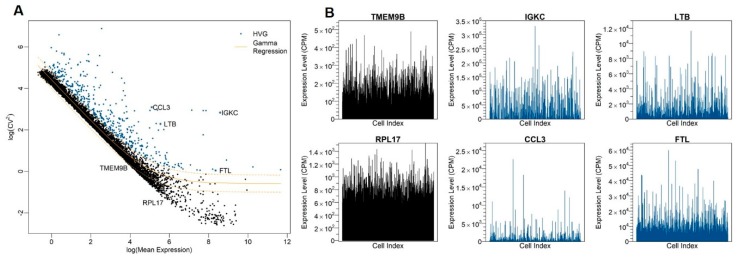
Identification of highly variable genes (HVGs). (**A**) The relationship between CV^2^ and mean expression of genes in LCL GM12878. The orange line shows the trend for the gamma GLM fit curve between CV^2^ and mean expression and used to identify HVGs. For each gene, the residual variability is calculated as the difference between observed CV^2^ and expected CV^2^ from the fitted curve. (**B**) Expression profiles of selected HVGs and lowly variable genes across cells. Cells are unsorted and remain a random order. Each vertical line is a cell, and the height of line indicates the level of gene expression in counts per million (CPM) in a cell.

After the μ-CV^2^ dependency correction, we identified 465, 466, and 364 HVGs at a false-discovery rate (FDR) of 0.01 for LCL, LAEC, and DF, respectively (Supplementary Tables S1–3). To visualize the expression variability of genes, we plot CV^2^ against μ, both on the logarithmic scale, for LCL (Figure 2A). Each dot represents a gene; all genes together give a characteristic cloud showing the μ and CV^2^ of gene expression. Genes above the GLM fitting curve, e.g., *IGKC*, *CCL3*, *LTB*, and *FTL*, are more variable than expectation, whereas genes below the curve, e.g., *TMEM9B* and *RPL17*, are less variable (Figure 2B).

### 3.3. Cell-Type Origin Determines the Function of Highly Variable Genes

To assess the biological functions of HVGs in different cell types, we performed enrichment analyses. We found that enriched gene ontology (GO) terms are largely distinct and reflect respective cell functions of each of the three cell types (Table 1). For example, LCL HVGs (e.g., *CCL22* and *IFI27*) are more likely to be involved in cytokine- or interferon-signaling pathways, and also, more generally, the innate immune system; LAEC HVGs (e.g., *COL1A1*, *MMP1*, and *IL17C*) collagen formation and extracellular matrix organization; DF HVGs (e.g., *KRT14*, *ACAN*, and *FLG*) keratinization and regulation of cell proliferation. DF HVGs also include *SFRP2*, *DPP4*, and *LSP1*, which are marker genes defining major fibroblast subpopulations in human skin [52]. Taken together, these results show that different cell types have different sets of HVGs with substantial scEV, associated with cell-type-specific functions.

If two cell types have shared function, then we expect to see the overlap in their HVG-associated functions. This, indeed, is the case. There are some overlaps between enriched functions between the three cell types we examined here. For example, the cytokine signaling pathway is enriched for both LCL and LAEC, and extracellular structure organization is enriched for both LAEC and DF. Meanwhile, across all three cell types, there are 14 shared HVGs genes (*CDC20*, *CLEC2B*, *CLIC3*, *CTSC*, *CYP1B1*, *DUSP2*, *HES1*, *MT1E*, *NPW*, *SOX4*, *STMN1*, *TK1*, *TRIB3*, and *UCHL1*; Supplementary Figure S5), with diverse cellular and molecular functions.

### 3.4. Functions Associated to HVGs are Conserved Across Tissues and Subpopulations of Cells

To determine how stable the functions associated with the HVGs identified in a cell type are, we used additional samples and random selection of the core cell. We analyzed two other fibroblast samples obtained from different body tissues (see Section 2.7. for data availability), as well as the initially included dermal sample. For each sample, after quality control, we randomly selected a core cell and their 999 more similar cells; then, we identified the set of HVGs (FDR <0.01 and fold-change >1.5) present in each subpopulation as described before (see Section 2. for materials and methods). Under these thresholds, we identified 221, 226, and 228 HVGs for dermal, lung distal, and lung proximal fibroblasts, respectively. Among the identified HVGs from the three samples, we found 36 genes that statistically enrich (FDR <0.05) for specific biological processes historically associated with fibroblasts (Table 2). The small overlap found, as well as the functional enrichment for the extracellular matrix, were previously described in [53,54], where it is shown that fibroblasts are a remarkably plastic cell type differing between human tissues where they develop unique morphologies and physiologic functions but still have a commonly associated role, the extracellular matrix organization, and maintenance.

### 3.5. HVGs as Part of the Regulatory Network with High Cell-Type Specificity

**Figure 3:**
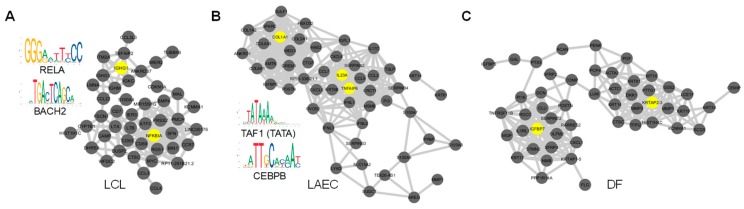
Co-expression networks of top HVGs. (**A**) Co-expression network between most-variable HVGs of LCL and two enriched binding motifs identified in these HVGs. (**B**) and (**C**) are for LAEC and DF, respectively. Genes labeled in yellow are the ones acting as a “hub” with high betweenness centrality and closely relevant to the cell-type function.

Next, we set out to test whether HVGs are co-expressed and thus tend to form co-expression networks [55]. We first imputed the expression matrix and then constructed the co-expressed network using the top 50 HVGs for each cell type. For LCLs, the network contains two main modules centered on *NFKBIA* and *IGHG1*, respectively (Figure 3A). *NFKBIA* encodes the NF-κB inhibitor that interacts with REL dimers to inhibit NF-κB/Rel complexes [56,57]. For LAECs, two modules are centered on *IL23A*/*TNFAIP6* and *COL1A1* (Figure 3B); for DF, *KRTAP2-3* and *IGFBP7* (Figure 3C). Thus, functions of “hub” genes in HVG co-expression networks are closely relevant to the function of corresponding cell type. These results are another line of evidence that scEV implies cell function. The transcription of multiple HVGs may be involved in the same underlying regulatory activities, giving rise to the co-expression network, as we observed. Thus, we wondered whether scEV in several different HVGs is driven by activities of one or few common TFs. To address this question, we searched for upstream regulators of the HVGs defined by our analysis (see Section 2 for materials and methods). We identified significant enriched TF binding motifs upstream of HVGs, four for LCL, and five for LAEC (Supplementary Table S4). No significantly enriched motif was identified for DF. The known motifs of LCL HVGs include that of the NF-κB subunit gene, *RELA*, and that of *BACH2* (Figure 3A). The known motifs of LAEC HVGs include the TATA box and that of *CEBPB* (Figure 3B).

**Figure 4:**
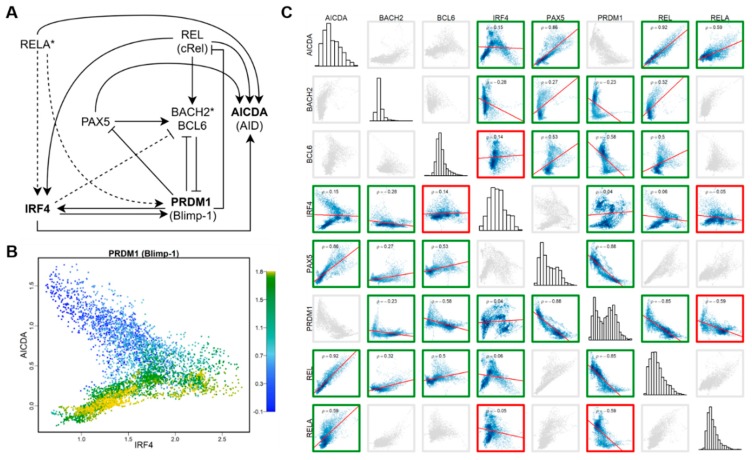
Gene regulatory network and correlation matrix of LCL HVGs. (**A**) An NF-κB regulatory network model for activated B cell (ABC)-antibody secreting cell (ASC) differentiation, modified from [60]. Bold font indicates HVGs; asterisk indicates the upstream TFs targeting HVGs; solid line dashed line indicates the regulatory relationship supported by the correlation between two corresponding genes, and the dashed line indicates regulatory relationship not supported by the expression correlation between genes. (**B**) Scatter plot of cells, showing the correlation between expression levels of three HVGs: *IRF4*, *AICDA* (AID), and *PRDM1* (Blimp-1). The color bar indicates the expression level of *PRDM1* (Blimp-1). (**C**) Spearman correlation matrix between expression levels of eight genes involved in the model. Green boxes indicate that the sign of the correlation between two genes is consistent with the effect (induction/repression) of the relationship between the two in the regulatory model. Red boxes indicate inconsistency, while gray boxes indicate no direct relationship according to the model.

To further explore the involvement of HVGs in the cell type-specific regulatory network, we focused on LCL HVGs in a well-studied gene regulatory network that orchestrates B cell fate dynamics [58,59,60]. This known regulatory network involves eight genes, including three LCL HVGs—*PRDM1* (or Blimp-1), *AICDA* (or AID), *IRF4*, two key regulatory genes with binding motifs enriched in targeting LCL HVGs (see above)—*RELA* and *BACH2*, and three other key regulators—*BCL6*, *PAX5*, and *REL* (cRel) (Figure 4A). 

We examined the inter-relationship between across-cell expressions of three LCL HVGs (Figure 4B). The scatter plot shows that the directionality of the correlation between *AICDA* and *IRF4* depends on the expression level of *PRDM1*. Among cells with relatively low expression of *PRDM1*, expressions of *AICDA* and *IRF4* are negatively correlated. Whereas, among cells in which *PRDM1* is highly expressed, expressions of *AICDA* and *IRF4* are positively correlated. This nonlinear relationship between expressions of HVGs suggests they are embedded in a tightly regulated expression network. Thus, we examined the all-by-all Spearman correlation between expressions of all eight genes in this regulatory network using the imputed data of the homogenous LCLs (Figure 4C). By comparing the sign of correlation coefficient of each pair of genes with the regulatory effect of the gene pair in the model network, we found that the correlation matrix can be used to correctly recover 15 out of 18 direct regulatory relationships. The result suggests that, even in this highly homogenous population of LCLs, cells retain gene regulatory network activities that orchestrate cell fate dynamics as in their original B cells.

### 3.6. Single-Cell Expression Variability in LCLs is Positively Correlated with between-Individual Expression Variability

Next, we examined the relationship between scEV and inter-individual expression variability. We distinguish between the two different types of variabilities at different organizational levels. Specifically, the former is cell-to-cell variability in a population of cells, and the latter is inter-individual variability at the human population level. We again focused on LCLs, for which population-scale gene expression data are available from the Geuvadis RNA-seq project of 1000 Genomes samples. The bulk RNA-seq data was downloaded as a normalized expression matrix of FPKM values. We retained data for all LCLs of European ancestry (CEU) [61]. With the residual variability estimated from scRNA-seq of GM12878 and that estimated from the CEU population, we tested the correlation between the two estimates across genes. When the test was conducted with all genes (*n* = 8424), we obtained a significant but weak positive correlation (SCC, *r* = 0.19, *p* = 1.2 × 10^−9^). We wondered whether this positive correlation was driven by subsets of genes. To identify these gene sets, we conducted the correlation tests for the GO-defined gene sets one by one. Across all gene sets tested, the average SCC for gene sets defined by GO biological process (BP) and molecular function (MF) terms are on average *r* = 0.28 and *r* = 0.23, respectively. Strikingly, we found a small number of gene sets that produced SCC much higher than averages. The functions of these gene sets include B-cell activation involved in immune response (GO:0002322), cytokine receptor activity (GO:0004896), cellular response to drug (GO:0035690), and regulation of tyrosine phosphorylation of stat protein (GO: 0042509; Figure 5), as well as leukocyte chemotaxis (GO: 0030595) and phospholipase activity (GO:0004620; for more examples, see Supplementary Figure S6). Thus, for these gene sets, scEV may contribute to the establishment of between-individual expression variability.

### 3.7. No Enriched Functions Associated with HVGs Identified in Human Induced Pluripotent Stem Cells (iPSCs)

**Figure 6:**
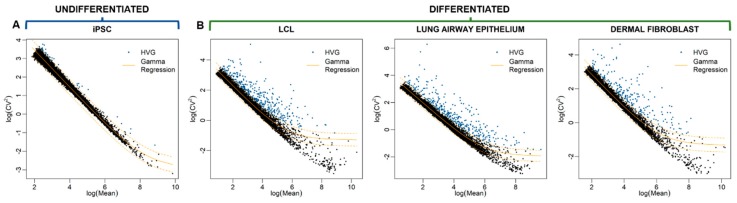
Comparison of the magnitude of single-cell expression variability (scEV) among genes between (**A**) undifferentiated induced pluripotent stem cells (iPSCs) and (**B**) three differentiated cell types: Lymphoblastoid cell line (LCL), lung airway epithelial cell (LAEC), and dermal fibroblast (DF). For each cell type, the relationship between coefficient of variation squared (CV^2^) and mean expression of genes is shown.

Finally, we argued, if scEV is the indicator of cell type-specific function, then scEV in undifferentiated cells should not be associated with any cellular functions. To test this, we obtained the scRNA-seq data from the study of [62] (see Section 2.7. for data availability). The data was generated from human iPSCs [63]. Same as other cell types examined in this study, these iPSCs were also prepared using the 10× Genomics Chromium controller. The released data contains five samples. We used the first batch (Sample 4) of the data to perform the HVG detection and function enrichment tests, using the same procedure applied to other cell types. When plotting the relationship between log-transformed CV^2^ and log-transformed average expression (μ), we found almost no genes showing large CV^2^ deviated from the regression curve (Figure 6A)—a pattern differs substantially from those of the other three cell types (Figure 6B). This pattern suggests that, for the majority of genes in iPSCs, scEV can be explained by technical noise or sampling stochasticity. In other words, iPSCs lack biological variability in their single-cell expression. Nevertheless, we still identified 79 iPSC HVGs (Supplementary Table S5) but could not associate any significant (FDR <0.05) enriched function with them. To further validate our findings, we performed the same analysis using another iPSC sample (see Section 2.7 for data availability) recovering the same pattern, a low number of HVGs (4) that are not significative enriched for a specific function (*ID3*, *LEFTY1*, *MALAT1*, *TAGLN*). These negative results are consistent with our prediction given by the “variation is function” hypothesis: undifferentiated iPSCs are not expected to be associated with any cell-type-specific function.

## 4. Discussion

Single-cell expression variability (or scEV) is sometimes called gene expression noise, emphasizing the stochastic nature of transcriptional activities in cells [64,65]. Interrogating scEV data has provided insights into gene regulatory architecture [66,67]; manipulating the magnitude of scEV, through using noise enhancers or scEV-modulating chemicals, has been an approach to achieve drug synergies [68]. Understanding the origin and functional implications of scEV has long been appreciated [4,5,6,69].

In this study, we focused on scEV in human cells. More specifically, we characterized different genes’ expression variability levels within a highly homogeneous population of genetically identical (or nearly isogenic) cells under the same environmental condition. We quantified scEV in highly homogeneous populations of a sizable number of viable cells. Working with cells of the same type, for example, LCL, we started by preprocessing data from thousands of cells. We found that, even though we had firstly preprocessed the data and retained only cells with similar library size and in the same cell cycle phase, it was not enough. There were still marked substructures, shown as branches of cells, in the embedding cloud of cells (Figure 1A), as revealed by the new embedding algorithm [30]. Retrospectively, we applied the trajectory analysis and found out that one of the longest branches contained cells with elevated expression of immunoglobulin genes (Supplementary Figure S7).

Similarly, marked substructures were observed in the embedding plots of the other two cell types, LAEC and DF. Genes that were differentially expressed and drove the formation of branches of LAECs and DFs were different from those in LCL cells. Thus, there is no single or a small set of marker genes that can be used to capture cellular heterogeneity across different cell types, making the definition of populations of homogenous cells a tedious task. Our work represents the first study focused on comparing scEV in highly homogeneous cell populations across genes in different cell types.

We showed that scEV estimated from homogeneous populations of cells for different cell types carries information on cell type-specific function. Information on molecular functions of cells and biological processes of a given cell type can be extracted from a set of highly variable genes (HVGs), bearing significant biological meaning (see also [70]). HVGs detected in different cell types do not overlap and can reveal the subtle differences in cellar functions between cell types. These conclusions are reached based on our investigation of three cell types and their corresponding HVGs. 

First, LCLs are usually established by in vitro infection of human peripheral blood lymphocytes by the Epstein–Barr virus. The viral infection selectively immortalizes resting B cells, giving rise to an actively proliferating B cell population [71]. B cells genetically diversity by rearranging the immunoglobulin locus to produce diverse antibody repertories that allow the immune system to recognize foreign molecules and initiate differential immune responses [20,72,73]. LCLs are produced through the rapid proliferation of few EBV-driven B cells from the blood cell population [74]. Thus, scRNA-seq data sets of LCLs offer a “snapshot” of highly diverse immunoglobulin rearrangement profiles in a much larger population of polyclonal B cells established in donors of these cell lines. Therefore, it is not unexpected to see quite a few immunoglobulin genes in the top list of HVGs identified in LCLs. In addition to these immunoglobulin genes, a number of other immune genes, especially C-C motif chemokine ligands (CCLs) and C-C motif chemokine receptors (CCRs), are in the list of HVGs of LCL. These genes play important roles in allowing the coordination of the activity of individual cells through intercellular communication, essential for the immune system maintains robustness [75]. The HVG co-expression network analysis revealed the key role of the NF-κB pathway in facilitating communications between immune cells [18,20]. More strikingly, we were able to reconstruct nearly the entire NF-κB regulatory network, underlying a differentiation of activated B cells and antibody-secreting cells, by using the correlation and anti-correlation relationships between expressions of HVGs and their regulatory genes.

Second, LAEC is a key cell type playing important roles in lung tissue remodeling, and pulmonary inflammatory and immune responses [76]. The airway epithelium, playing a critical role in conducting air to and from the alveoli, is a dynamic tissue that normally undergoes slow but constant turnover. In the event of mild to moderate injury, the airway epithelium responds vigorously to re-establish an epithelial sheet with normal structure and function. HVGs identified in LAECs, which are enriched with genes involved in collagen formation, regulation of cell proliferation, and extracellular matrix organization, accurately elucidate this aspect of functions of the airway epithelium. LAECs are also central to the defense of the lung against pathogens and particulates that are inhaled from the environment. This aspect of functions is also reflected in the enriched functionality of LAEC HVGs.

Third, DFs are responsible for generating connective tissue and play a critical role in normal wound healing [53]. DFs are also commonly used in immunological studies [33,77,78]. HVGs identified in DFs again accurately reflect these primary aspects of DF functions, including extracellular matrix organization, keratinization, and regulation of signaling receptor activity. DF HVGs do have several categories of enriched functions overlap with those of LAEC, which is not unexpected, given that DF and LAEC have functional overlaps [79].

Our results provide evidence supporting the “variation is function” hypothesis, proposed by [17], suggesting that the aggregate cellular function may depend on scEV. Dueck and colleagues also laid down several scenarios, including bet hedging, response distribution, fate plasticity, and so on, in which the establishment of the relationship between scEV and cell function could be attained. Our analytical framework using scRNA-seq data may be utilized in appropriate systems to test the plausibility of these different scenarios. If scEV is an accountable and credible surrogate of cell function, as we have shown in this study, then quantifying and characterizing scEV may become a first-line approach for understanding the function of cell types and tissues. Indeed, when we applied this framework to scRNA-seq data from human iPSCs, we observed no enriched gene functions and no regulatory pathways/networks associated with HVGs in iPSCs. This anti-example, showing no variation no function, further validates the “variation is function” hypothesis.

Furthermore, we have shown that, across certain sets of genes, scEV is positively correlated with population-level expression variability. This correlation provides a new possibility to design single-cell assays with one sample to approximate the population variability of certain genes’ expression. This new method may be used to study disease-causing expression dysregulation because it has been a number of cases that increased population-level expression variability has been linked with diseases [80,81,82,83,84].

Pelkmans [8] pointed out in a visionary perspective article that: “Embracing this cell-to-cell variability as a fact in our scientific understanding requires a paradigm shift, but it will be necessary.” Indeed, scRNA-seq technologies have brought revolution to gene expression analysis. The technical development gives us a new approach beyond the capacity of traditional methods that rely on experimental measurements of population-average behavior of cells to conceive regulatory network models and signal processing pathways. More importantly, for traditional methods, by averaging information across many cells, differences among cells, which may be important in explaining mechanisms, can be lost. Given the large degree of cell-to-cell expression variability even between genetically homogeneous cells, conclusions reached as for such with traditional average-based methods may be of low-resolution, incomplete, and sometimes misleading [3,18,29,85].

## 5. Conclusions

We have shown that scEV in highly homogeneous populations of human cells is widespread in differentiated cell types and is likely to imply cell type-specific function. We conclude that single-cell variability and the information it contains are the key to a deepened understanding of cells and their functions. Careful assessment and characterization of cell-to-cell expression variability in relevant cell types will facilitate the study of normal cell functions as well as pathological cell processes.

## Figures and Tables

**Figure 5:**
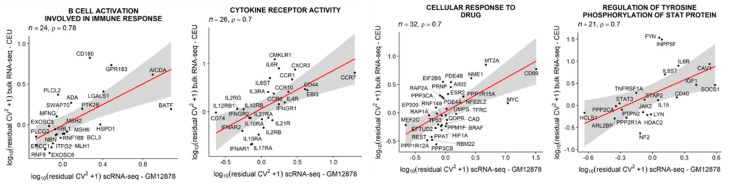
Correlation between scEV (i.e., residual variability estimated from LCL GM12878) and the population-level expression variability (measured in LCLs derived from unrelated individuals of European ancestry, CEU) between genes of selected gene sets. More examples can be found in Supplementary Figure S6.

**Table 1:** Representative HVGs identified in the three cell types: LCL, LAEC, and DF, and the results of functional enrichment analyses. Genes are sorted by residual variability. The top 50 genes with the highest residual variability values are selected as representative HVGs.

Cell Type	Highly Variable Genes (HVGs), Top 50	Enriched GO Terms, Top 10	Enriched Reactome Pathways, Top 10
Lymphoblastoid Cell line (LCL)	*ANKRD37 ATF3 BIN1 BMP4 CAMP CCL22 CCL3 CCL3L3 CCL4 CCL4L2 CCR7 CD69 CD7 CD83 CDKN1A CTSC CYP1B1 DHRS9 DUSP2 FSCN1 HIST1H1C IER3 IFI27 IGHG1 IGHG3 IGHM IGKC ITM2A KCNMA1 LINC00176 LINC01588 LMNA LTA LTB MAL MIER2 MIR155HG MYC NFKBIA PMCH PRSS2 RGS1 RGS16 RGS2 RP11-291B21.2 S100A4 SFN TNFAIP2 TUBB4B WFDC2*	Signal transduction (GO:0007165) Response to stimulus (GO:0050896) Immune response (GO:0006955) Response to biotic stimulus (GO:0009607) Immune system process (GO:0002376) Response to external biotic stimulus (GO:0043207) Response to external stimulus (GO:0009605) Cytokine-mediated signaling pathway (GO:0019221) Defense response (GO:0006952) Response to chemical (GO:0042221)	Immune system (R-HSA-168256) Chemokine receptors bind chemokines (R-HSA-380108) Interferon alpha/beta signaling (R-HSA-909733) Cytokine signaling in immune system (R-HSA-1280215) Interferon signaling (R-HSA-913531) Peptide ligand-binding receptors (R-HSA-375276) G alpha (i) signaling events (R-HSA-418594) Innate Immune System (R-HSA-168249) Interferon gamma signaling (R-HSA-877300) Cell cycle (R-HSA-1640170)
Lung airway epithelial cell (LAEC)	*AMTN ANKRD1 AREG CCL2 CCL5 CCL7 COL1A1 COL1A2 COL3A1 COL6A1 COL6A3 CRCT1 CTGF CXCL5 CXCL6 FBXO32 GREM1 HAS2 IFNL1 IFNL2 IFNL3 IGFBP5 IGFL1 IL17C IL23A KRT14 KRT6B KRT81 LY6D MEG3 MMP1 MSMB OVOS2 PI3 POSTN PPBP RP11-338I21.1 S100A7 S100A8 S100A9 SERPINB2 SERPINB3 SERPINB4 SLC15A2 SPARC SUGCT SULF1 TEX26-AS1 TNFAIP6 TSLP*	Regulation of multicellular organismal process (GO:0051239) Regulation of signaling receptor activity (GO:0010469) Response to stimulus (GO:0050896) Regulation of cell proliferation (GO:0042127) Developmental process (GO:0032502) Extracellular matrix organization (GO:0030198) Response to chemical (GO:0042221) Response to organic substance (GO:0010033) Regulation of developmental process (GO:0050793) Regulation of response to stimulus (GO:0048583)	Extracellular matrix organization (R-HSA-1474244) Assembly of collagen fibrils and other multimeric structures (R-HSA-2022090) Cytokine signaling in immune system (R-HSA-1280215) Collagen formation (R-HSA-1474290) Signaling by interleukins (R-HSA-449147) Chemokine receptors bind chemokines (R-HSA-380108) Peptide ligand-binding receptors (R-HSA-375276) Collagen biosynthesis and modifying enzymes (R-HSA-1650814) Integrin cell surface interactions (R-HSA-216083) Class A/1 (rhodopsin-like receptors) (R-HSA-373076)
Dermal fibroblast (DF)	*ACAN ACTA2 ACTC1 CEMIP CLU COMP CTSC CXCL1 DCN DKK1 FLG G0S2 GAL HIST1H4C IGFBP5 IGFBP7 IL1RL1 KCNMA1 KRT14 KRT17 KRT19 KRT34 KRT81 KRTAP1-5 KRTAP2-3 LCE1F LUM MGP MMP1 MMP3 MT1X NMB OLFM2 PCP4 PENK PGF PI16 POSTN PPP1R14A PTTG1 PTX3 RARRES2 RGCC SCG5 SERPINE2 SFRP2 SFRP4 STMN2 TFPI2 TNFRSF11B*	Regulation of signaling receptor activity (GO:0010469) Developmental process (GO:0032502) Keratinization (GO:0031424) Anatomical structure development (GO:0048856) Regulation of cell proliferation (GO:0042127) Regulation of multicellular organismal process (GO:0051239) Extracellular matrix organization (GO:0030198) Extracellular structure organization (GO:0043062) Response to oxygen-containing compound (GO:1901700) Multicellular organismal process (GO:0032501)	Extracellular matrix organization (R-HSA-1474244) Regulation of insulin-like growth factor (IGF) transport and uptake by insulin-like growth factor binding proteins (IGFBPs) (R-HSA-381426) ECM proteoglycans (R-HSA-3000178) Hemostasis (R-HSA-109582) Platelet degranulation (R-HSA-114608) Dissolution of fibrin clot (R-HSA-75205 Response to elevated platelet cytosolic Ca2+ (R-HSA-76005) Negative regulation of TCF-dependent signaling by WNT ligand antagonists (R-HSA-3772470) GPCR ligand binding (R-HSA-500792) Peptide ligand-binding receptors (R-HSA-375276)

**Table 2:** Shared HVGs identified in the three fibroblast samples: dermal, lung distal, and lung proximal, and the results of functional enrichment analysis.

Shared Highly Variable Genes (HVGs)	Enriched GO Terms, Top 5	Enriched Reactome Pathways
*ID3 MARCKSL1 DPT ID2 CYP1B1 IGFBP2 IGFBP5 APOD IGFBP7 SFRP2 PLK2 SOX4 PI16 CTGF SGK1 CITED2 IGFBP3 SERPINE1 TIMP1 OSR2 HAS2 MYC B4GALT1 PTGDS CRYAB BAMBI MFAP5 CSRP2 LUM PGF THBS1 FGF7 MT2A MT1X WISP2 ADAMTS1*	Collagen-containing extracellular matrix (GO:0062023) Extracellular matrix (GO:0031012) Epithelial cell proliferation (GO:0050673) Negative regulation of cell migration (GO:0030336) Extracellular matrix organization (GO:0030198)	Extracellular matrix organization (R-HSA-1474244) Regulation of insulin-like growth factor (IGF) transport and uptake by insulin-like growth factor binding proteins (IGFBPs) (R-HSA-381426)

## Supplementary Materials

The following are available online at https://www.mdpi.com/2073-4409/9/1/14/s1, Figure S1: Flowchart of selection of a highly homogeneous population of cells; Figure S2: UMAP embedding plots for the 1000 selected cells of each of three cell types. Figure S3: Scatter plots between mean and residual CV2. Figure S4: Comparison between two methods: HVG (Brennecke et al. 2013) and VEG (Chen et al. 2016), for correcting mean-variance dependency. Figure S5: Venn diagram showing overlap between HVGs identified in three cell types. Figure S6: Correlation between scEV and population-level expression variability across genes of functional sets. Figure S7: PHATE 3-D embedding plot for cells colored according to *IGLC2* expression level in cells. Table S1: HVGs of lymphoblastoid cell line (LCL) cells (FDR <0.01). Table S2: HVGs of lung airway epithelial cells (LAECs) (FDR <0.01). Table S3: HVGs of dermal fibroblasts (DFs) (FDR <0.01). Table S4: Significantly enriched motifs found in HVGs. Table S5: HVGs of induced pluripotent stem cell (iPSCs) (FDR <0.01).

## References

[1] Zhang X., Li T., Liu F., Chen Y., Yao J., Li Z., Huang Y., Wang J. (2019). Comparative Analysis of Droplet-Based Ultra-High-Throughput Single-Cell RNA-Seq Systems. Mol. Cell.

[2] Buettner F., Natarajan K.N., Casale F.P., Proserpio V., Scialdone A., Theis F.J., Teichmann S.A., Marioni J.C., Stegle O. (2015). Computational analysis of cell-to-cell heterogeneity in single-cell RNA-sequencing data reveals hidden subpopulations of cells. Nat. Biotechnol..

[3] Trapnell C. (2015). Defining cell types and states with single-cell genomics. Genome Res..

[4] Ko M.S. (1992). Induction mechanism of a single gene molecule: Stochastic or deterministic?. Bioessays.

[5] Fiering S., Whitelaw E., Martin D.I. (2000). To be or not to be active: The stochastic nature of enhancer action. Bioessays.

[6] Raj A., van Oudenaarden A. (2008). Nature, nurture, or chance: Stochastic gene expression and its consequences. Cell.

[7] Eldar A., Elowitz M.B. (2010). Functional roles for noise in genetic circuits. Nature.

[8] Pelkmans L. (2012). Cell Biology. Using cell-to-cell variability--a new era in molecular biology. Science.

[9] Kumar P., Tan Y., Cahan P. (2017). Understanding development and stem cells using single cell-based analyses of gene expression. Development.

[10] Wernet M.F., Mazzoni E.O., Celik A., Duncan D.M., Duncan I., Desplan C. (2006). Stochastic spineless expression creates the retinal mosaic for colour vision. Nature.

[11] Chang H.H., Hemberg M., Barahona M., Ingber D.E., Huang S. (2008). Transcriptome-wide noise controls lineage choice in mammalian progenitor cells. Nature.

[12] Faure A.J., Schmiedel J.M., Lehner B. (2017). Systematic Analysis of the Determinants of Gene Expression Noise in Embryonic Stem Cells. Cell Syst..

[13] Martinez-Jimenez C.P., Eling N., Chen H.C., Vallejos C.A., Kolodziejczyk A.A., Connor F., Stojic L., Rayner T.F., Stubbington M.J.T., Teichmann S.A. (2017). Aging increases cell-to-cell transcriptional variability upon immune stimulation. Science.

[14] Wiley C.D., Flynn J.M., Morrissey C., Lebofsky R., Shuga J., Dong X., Unger M.A., Vijg J., Melov S., Campisi J. (2017). Analysis of individual cells identifies cell-to-cell variability following induction of cellular senescence. Aging Cell.

[15] Azizi E., Carr A.J., Plitas G., Cornish A.E., Konopacki C., Prabhakaran S., Nainys J., Wu K., Kiseliovas V., Setty M. (2018). Single-Cell Map of Diverse Immune Phenotypes in the Breast Tumor Microenvironment. Cell.

[16] Segerstolpe A., Palasantza A., Eliasson P., Andersson E.M., Andreasson A.C., Sun X., Picelli S., Sabirsh A., Clausen M., Bjursell M.K. (2016). Single-Cell Transcriptome Profiling of Human Pancreatic Islets in Health and Type 2 Diabetes. Cell Metab..

[17] Dueck H., Eberwine J., Kim J. (2016). Variation is function: Are single cell differences functionally important?: Testing the hypothesis that single cell variation is required for aggregate function. Bioessays.

[18] Tay S., Hughey J.J., Lee T.K., Lipniacki T., Quake S.R., Covert M.W. (2010). Single-cell NF-kappaB dynamics reveal digital activation and analogue information processing. Nature.

[19] Raj A., Rifkin S.A., Andersen E., van Oudenaarden A. (2010). Variability in gene expression underlies incomplete penetrance. Nature.

[20] Mitchell S., Roy K., Zangle T.A., Hoffmann A. (2018). Nongenetic origins of cell-to-cell variability in B lymphocyte proliferation. Proc. Natl. Acad. Sci. USA.

[21] Snijder B., Sacher R., Ramo P., Damm E.M., Liberali P., Pelkmans L. (2009). Population context determines cell-to-cell variability in endocytosis and virus infection. Nature.

[22] Kernfeld E.M., Genga R.M.J., Neherin K., Magaletta M.E., Xu P., Maehr R. (2018). A Single-Cell Transcriptomic Atlas of Thymus Organogenesis Resolves Cell Types and Developmental Maturation. Immunity.

[23] Miragaia R.J., Zhang X., Gomes T., Svensson V., Ilicic T., Henriksson J., Kar G., Lonnberg T. (2018). Single-cell RNA-sequencing resolves self-antigen expression during mTEC development. Sci. Rep..

[24] McDavid A., Dennis L., Danaher P., Finak G., Krouse M., Wang A., Webster P., Beechem J., Gottardo R. (2014). Modeling bi-modality improves characterization of cell cycle on gene expression in single cells. PLoS Comput. Biol..

[25] Svensson V., Vento-Tormo R., Teichmann S.A. (2018). Exponential scaling of single-cell RNA-seq in the past decade. Nat. Protoc..

[26] Geiler-Samerotte K.A., Bauer C.R., Li S., Ziv N., Gresham D., Siegal M.L. (2013). The details in the distributions: Why and how to study phenotypic variability. Curr. Opin. Biotechnol..

[27] Mar J.C. (2019). The rise of the distributions: Why non-normality is important for understanding the transcriptome and beyond. Biophys. Rev..

[28] Marinov G.K., Williams B.A., McCue K., Schroth G.P., Gertz J., Myers R.M., Wold B.J. (2014). From single-cell to cell-pool transcriptomes: Stochasticity in gene expression and RNA splicing. Genome Res..

[29] Li B., You L. (2013). Predictive power of cell-to-cell variability. Quant. Biol..

[30] Moon K.R., van Dijk D., Wang Z., Gigante S., Burkhardt D., Chen W., Yim K., Elzen A.V.D., Hirn M.J., Coifman R.R. (2019). Visualizing structure and transitions in high-dimensional biological data. Nat Biotechnol..

[31] Osorio D., Yu X., Yu P., Serpedin E., Cai J.J. (2019). Single-cell RNA sequencing of a European and an African lymphoblastoid cell line. Sci. Data.

[32] Habiel D.M., Espindola M.S., Jones I.C., Coelho A.L., Stripp B., Hogaboam C.M. (2018). CCR10+ epithelial cells from idiopathic pulmonary fibrosis lungs drive remodeling. Jci Insight.

[33] Hagai T., Chen X., Miragaia R.J., Rostom R., Gomes T., Kunowska N., Henriksson J., Park J.E., Proserpio V., Donati G. (2018). Gene expression variability across cells and species shapes innate immunity. Nature.

[34] Friedman C.E., Nguyen Q., Lukowski S.W., Helfer A., Chiu H.S., Miklas J., Levy S., Suo S., Han J.-D.J., Osteil P. (2018). Single-cell transcriptomic analysis of cardiac differentiation from human PSCs reveals HOPX-dependent cardiomyocyte maturation. Cell Stem Cell.

[35] Butler A., Hoffman P., Smibert P., Papalexi E., Satija R. (2018). Integrating single-cell transcriptomic data across different conditions, technologies, and species. Nat. Biotechnol..

[36] Brennecke P., Anders S., Kim J.K., Kolodziejczyk A.A., Zhang X., Proserpio V., Baying B., Benes V., Teichmann S.A., Marioni J.C. (2013). Accounting for technical noise in single-cell RNA-seq experiments. Nat. Methods.

[37] Cai J.J. (2019). scGEAToolbox: A Matlab toolbox for single-cell RNA sequencing data analysis. Bioinformatics.

[38] Benjamini Y., Hochberg Y. (1995). Controlling the false discovery rate: A practical and powerful approach to multiple testing. J. Roy. Stat. Soc. Ser. B.

[39] Kuleshov M.V., Jones M.R., Rouillard A.D., Fernandez N.F., Duan Q., Wang Z., Koplev S., Jenkins S.L., Jagodnik K.M., Lachmann A. (2016). Enrichr: A comprehensive gene set enrichment analysis web server 2016 update. Nucleic Acids Res..

[40] Chen E.Y., Tan C.M., Kou Y., Duan Q., Wang Z., Meirelles G.V., Clark N.R., Ma’ayan A. (2013). Enrichr: Interactive and collaborative HTML5 gene list enrichment analysis tool. Bmc Bioinform..

[41] Eden E., Navon R., Steinfeld I., Lipson D., Yakhini Z. (2009). GOrilla: A tool for discovery and visualization of enriched GO terms in ranked gene lists. Bmc Bioinform..

[42] Fabregat A., Jupe S., Matthews L., Sidiropoulos K., Gillespie M., Garapati P., Haw R., Jassal B., Korninger F., May B. (2018). The Reactome Pathway Knowledgebase. Nucleic Acids Res..

[43] van Dijk D., Sharma R., Nainys J., Yim K., Kathail P., Carr A.J., Burdziak C., Moon K.R., Chaffer C.L., Pattabiraman D. (2018). Recovering Gene Interactions from Single-Cell Data Using Data Diffusion. Cell.

[44] Konganti K., Wang G., Yang E., Cai J.J. (2013). SBEToolbox: A Matlab toolbox for biological network analysis. Evol. Bioinform. Online.

[45] McLean C.Y., Bristor D., Hiller M., Clarke S.L., Schaar B.T., Lowe C.B., Wenger A.M., Bejerano G. (2010). GREAT improves functional interpretation of cis-regulatory regions. Nat. Biotechnol..

[46] Smedley D., Haider S., Ballester B., Holland R., London D., Thorisson G., Kasprzyk A. (2009). BioMart--biological queries made easy. Bmc Genom..

[47] Khan A., Fornes O., Stigliani A., Gheorghe M., Castro-Mondragon J.A., van der Lee R., Bessy A., Cheneby J., Kulkarni S.R., Tan G. (2018). JASPAR 2018: Update of the open-access database of transcription factor binding profiles and its web framework. Nucleic Acids Res..

[48] Kivioja T., Vaharautio A., Karlsson K., Bonke M., Enge M., Linnarsson S., Taipale J. (2011). Counting absolute numbers of molecules using unique molecular identifiers. Nat. Methods.

[49] van der Maaten L., Hinton G. (2008). Visualizing Data using t-SNE. J. Mach. Learn. Res..

[50] Becht E., McInnes L., Healy J., Dutertre C.A., Kwok I.W.H., Ng L.G., Ginhoux F., Newell E.W. (2018). Dimensionality reduction for visualizing single-cell data using UMAP. Nat. Biotechnol..

[51] Chen H.I., Jin Y., Huang Y., Chen Y. (2016). Detection of high variability in gene expression from single-cell RNA-seq profiling. Bmc Genom..

[52] Tabib T., Morse C., Wang T., Chen W., Lafyatis R. (2018). SFRP2/DPP4 and FMO1/LSP1 Define Major Fibroblast Populations in Human Skin. J. Invest. Derm..

[53] Tracy L.E., Minasian R.A., Caterson E.J. (2016). Extracellular Matrix and Dermal Fibroblast Function in the Healing Wound. Adv. Wound Care.

[54] Singhal P.K., Sassi S., Lan L., Au P., Halvorsen S.C., Fukumura D., Jain R.K., Seed B. (2016). Mouse embryonic fibroblasts exhibit extensive developmental and phenotypic diversity. Proc. Natl. Acad. Sci. USA.

[55] Mantsoki A., Devailly G., Joshi A. (2016). Gene expression variability in mammalian embryonic stem cells using single cell RNA-seq data. Comput. Biol. Chem..

[56] Courtois G., Smahi A., Reichenbach J., Doffinger R., Cancrini C., Bonnet M., Puel A., Chable-Bessia C., Yamaoka S., Feinberg J. (2003). A hypermorphic IkappaBalpha mutation is associated with autosomal dominant anhidrotic ectodermal dysplasia and T cell immunodeficiency. J. Clin. Invest..

[57] Lopez-Granados E., Keenan J.E., Kinney M.C., Leo H., Jain N., Ma C.A., Quinones R., Gelfand E.W., Jain A. (2008). A novel mutation in NFKBIA/IKBA results in a degradation-resistant N-truncated protein and is associated with ectodermal dysplasia with immunodeficiency. Hum. Mutat..

[58] Nutt S.L., Hodgkin P.D., Tarlinton D.M., Corcoran L.M. (2015). The generation of antibody-secreting plasma cells. Nat. Rev. Immunol..

[59] Sciammas R., Li Y., Warmflash A., Song Y., Dinner A.R., Singh H. (2011). An incoherent regulatory network architecture that orchestrates B cell diversification in response to antigen signaling. Mol. Syst. Biol..

[60] Roy K., Mitchell S., Liu Y., Ohta S., Lin Y.S., Metzig M.O., Nutt S.L., Hoffmann A. (2019). A Regulatory Circuit Controlling the Dynamics of NFkappaB cRel Transitions B Cells from Proliferation to Plasma Cell Differentiation. Immunity.

[61] Lappalainen T., Sammeth M., Friedlander M.R., AC’t Hoen P.A., Monlong J., Rivas M.A., Gonzalez-Porta M., Kurbatova N., Griebel T., Ferreira P.G. (2013). Transcriptome and genome sequencing uncovers functional variation in humans. Nature.

[62] Nguyen Q.H., Lukowski S.W., Chiu H.S., Senabouth A., Bruxner T.J.C., Christ A.N., Palpant N.J., Powell J.E. (2018). Single-cell RNA-seq of human induced pluripotent stem cells reveals cellular heterogeneity and cell state transitions between subpopulations. Genome Res..

[63] Mandegar M.A., Huebsch N., Frolov E.B., Shin E., Truong A., Olvera M.P., Chan A.H., Miyaoka Y., Holmes K., Spencer C.I. (2016). CRISPR Interference Efficiently Induces Specific and Reversible Gene Silencing in Human iPSCs. Cell Stem Cell.

[64] Kaern M., Elston T.C., Blake W.J., Collins J.J. (2005). Stochasticity in gene expression: From theories to phenotypes. Nat. Rev. Genet..

[65] Raser J.M., O’Shea E.K. (2005). Noise in gene expression: Origins, consequences, and control. Science.

[66] Chalancon G., Ravarani C.N., Balaji S., Martinez-Arias A., Aravind L., Jothi R., Babu M.M. (2012). Interplay between gene expression noise and regulatory network architecture. Trends Genet..

[67] Mar J.C., Matigian N.A., Mackay-Sim A., Mellick G.D., Sue C.M., Silburn P.A., McGrath J.J., Quackenbush J., Wells C.A. (2011). Variance of gene expression identifies altered network constraints in neurological disease. PLoS Genet..

[68] Dar R.D., Hosmane N.N., Arkin M.R., Siliciano R.F., Weinberger L.S. (2014). Screening for noise in gene expression identifies drug synergies. Science.

[69] Ecker S., Pancaldi V., Valencia A., Beck S., Paul D.S. (2018). Epigenetic and Transcriptional Variability Shape Phenotypic Plasticity. Bioessays.

[70] Dueck H., Khaladkar M., Kim T.K., Spaethling J.M., Francis C., Suresh S., Fisher S.A., Seale P., Beck S.G., Bartfai T. (2015). Deep sequencing reveals cell-type-specific patterns of single-cell transcriptome variation. Genome Biol..

[71] Neitzel H. (1986). A routine method for the establishment of permanent growing lymphoblastoid cell lines. Hum. Genet..

[72] Tonegawa S. (1983). Somatic generation of antibody diversity. Nature.

[73] Papavasiliou F., Casellas R., Suh H., Qin X.F., Besmer E., Pelanda R., Nemazee D., Rajewsky K., Nussenzweig M.C. (1997). V(D)J recombination in mature B cells: A mechanism for altering antibody responses. Science.

[74] Ryan J.L., Kaufmann W.K., Raab-Traub N., Oglesbee S.E., Carey L.A., Gulley M.L. (2006). Clonal evolution of lymphoblastoid cell lines. Lab. Invest..

[75] Altan-Bonnet G., Mukherjee R. (2019). Cytokine-mediated communication: A quantitative appraisal of immune complexity. Nat. Rev. Immunol..

[76] Hiemstra P.S., McCray P.B., Bals R. (2015). The innate immune function of airway epithelial cells in inflammatory lung disease. Eur Respir J..

[77] Zhao M., Zhang J., Phatnani H., Scheu S., Maniatis T. (2012). Stochastic expression of the interferon-beta gene. PLoS Biol..

[78] Sacco O., Silvestri M., Sabatini F., Sale R., Defilippi A.C., Rossi G.A. (2004). Epithelial cells and fibroblasts: Structural repair and remodelling in the airways. Paediatr Respir Rev..

[79] Huang G., Osorio D., Guan J., Ji G., Cai J.J. (2018). Overdispersed gene expression characterizes schizophrenic brains. bioRxiv.

[80] Guan J., Yang E., Yang J., Zeng Y., Ji G., Cai J.J. (2016). Exploiting aberrant mRNA expression in autism for gene discovery and diagnosis. Hum. Genet..

[81] Ecker S., Pancaldi V., Rico D., Valencia A. (2015). Higher gene expression variability in the more aggressive subtype of chronic lymphocytic leukemia. Genome Med..

[82] Li J., Liu Y., Kim T., Min R., Zhang Z. (2010). Gene expression variability within and between human populations and implications toward disease susceptibility. PLoS Comput. Biol..

[83] Spreizer S., Aertsen A., Kumar A. (2019). From space to time: Spatial inhomogeneities lead to the emergence of spatiotemporal sequences in spiking neuronal networks. PLoS Computat. Biol..

[84] Ho J.W., Stefani M., dos Remedios C.G., Charleston M.A. (2008). Differential variability analysis of gene expression and its application to human diseases. Bioinformatics.

[85] Bendall S.C., Nolan G.P. (2012). From single cells to deep phenotypes in cancer. Nat. Biotechnol..

