# Supplementary material for: Single-Cell Expression Variability Implies Cell Function
Source: Cells. 2019 Dec 19;9(1):14. doi: 10.3390/cells9010014 (PMC7017299; doi:10.3390/cells9010014)

Single-cell expression variability implies cell function

Daniel Osorio[1,#], Xue Yu[2,#], Yan Zhong[3,#], Guanxun Li[3], Peng Yu[4], Erchin Serpedin[4], Jianhua Huang[3,*], James J. Cai[1,4,5,*]

[1]Department of Veterinary Integrative Biosciences; [2]Department of Veterinary Pathobiology; [3]Department of Statistics; [4]Department of Electrical and Computer Engineering; [5]Interdisciplinary Program of Genetics; Texas A&M University, College Station, TX 77843, USA.

[#]These authors contributed equally to this work.

[*]Corresponding authors: JH (jianhua@stat.tamu.edu) and JJC (jcai@tamu.edu)

Supplementary Figures

Supplementary Fig. S1. Flowchart of selection of a highly homogeneous population of cells........ 2

Supplementary Fig. S2. UMAP embedding plots for the 1,000 selected cells of each of three cell types........ 3

Supplementary Fig. S3. Scatter plots between mean and residual CV^2. 4

Supplementary Fig. S4. Comparison between two methods: HVG (Brennecke et al. 2013) and VEG (Chen et al. 2016), for correcting mean-variance dependency. 5

Supplementary Fig. S5. Venn diagram showing overlap between HVGs identified in three cell types........ 6

Supplementary Fig. S6. Correlation between scEV and population-level expression variability across genes of functional sets. 7

Supplementary Fig. S7. The relationship between CV2 and mean expression of genes in human iPSCs. 9

Supplementary Fig. S8. PHATE 3-D embedding plot for cells colored according to IGLC2 expression level in cells. 10

Supplementary Fig. S1. Flowchart of selection of a highly homogeneous population of cells.

Supplementary Fig. S1. Flowchart of selection of a highly homogeneous population of cells.

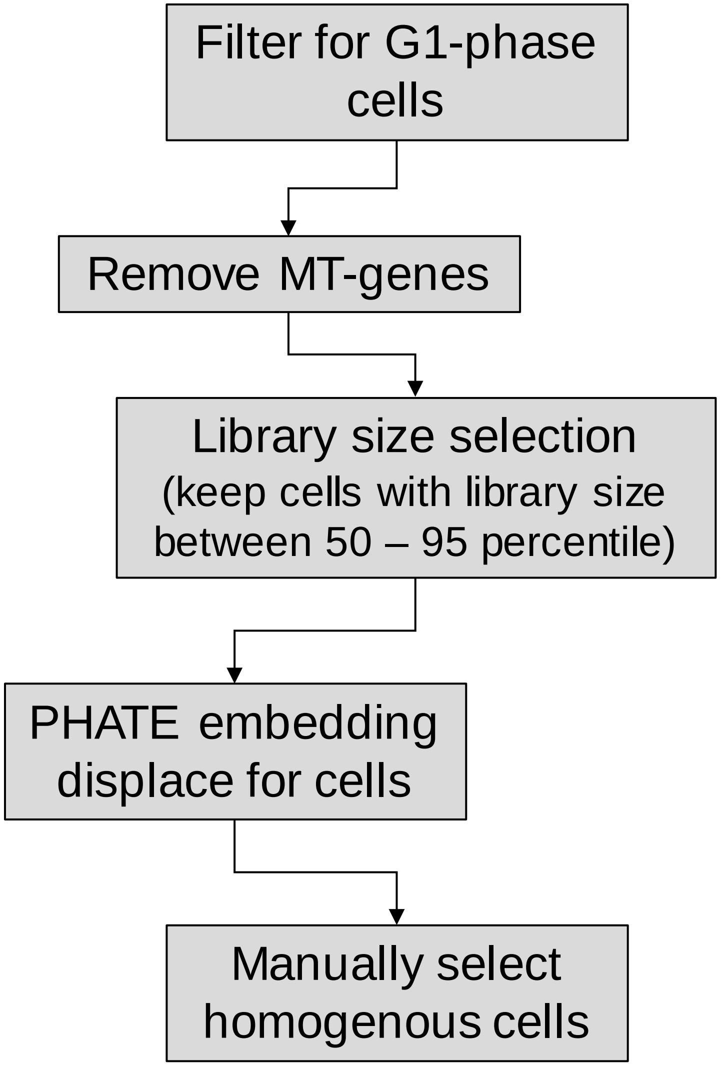

Supplementary Fig. S2. UMAP embedding plots for the 1,000 selected cells of each of three cell types.

Supplementary Fig. S2. UMAP embedding plots for the 1,000 selected cells of each of three cell types.

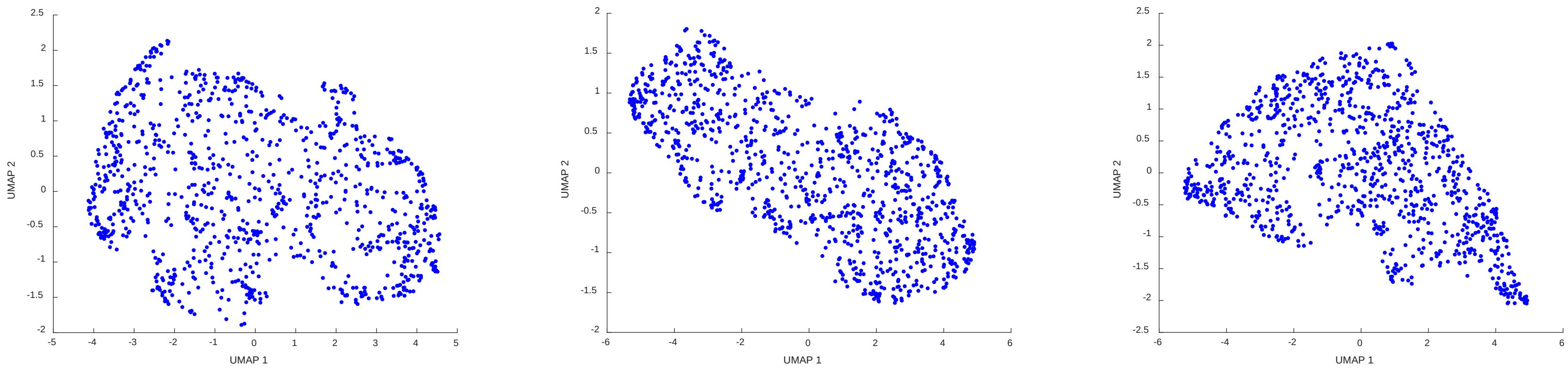

Supplementary Fig. S3. Scatter plots between mean and residual CV2.

Supplementary Fig. S3. Scatter plots between mean and residual CV^2.

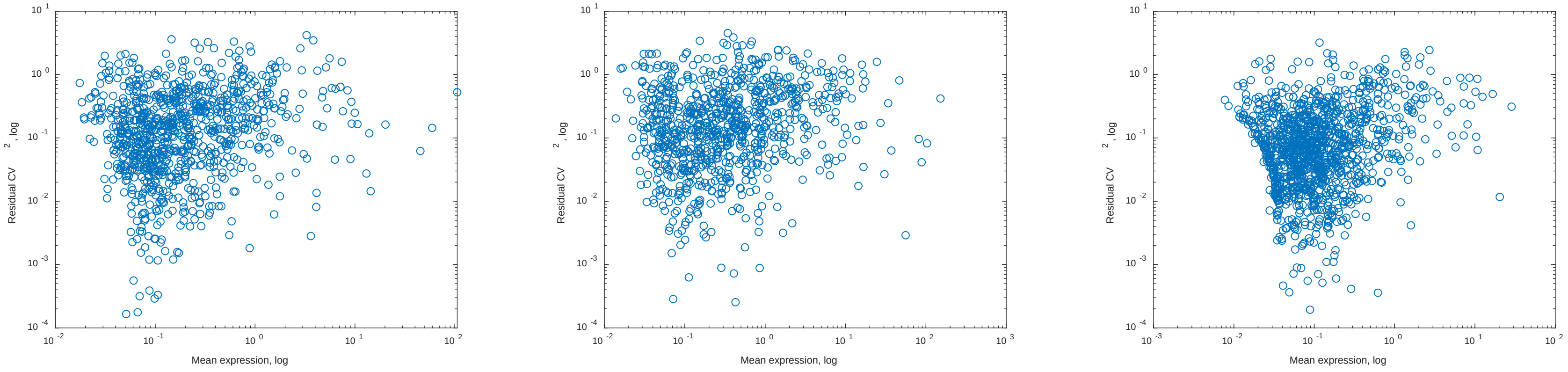

Supplementary Fig. S4. Comparison between two methods: HVG (Brennecke et al. 2013) and VEG (Chen et al. 2016), for correcting mean-variance dependency.

Supplementary Fig. S4. Comparison between two methods: HVG (Brennecke et al. 2013) and VEG (Chen et al. 2016), for correcting mean-variance dependency.

For each cell type, the numbers in parentheses are numbers of HVGs identified using the two methods and the number of overlap genes.

	LCL	LAEC	DF
HVG	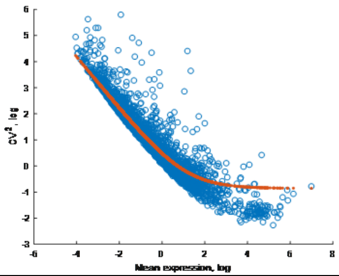	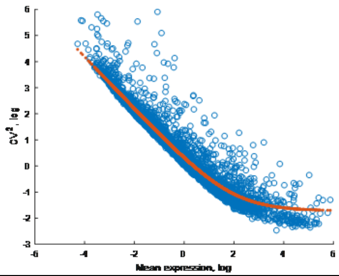	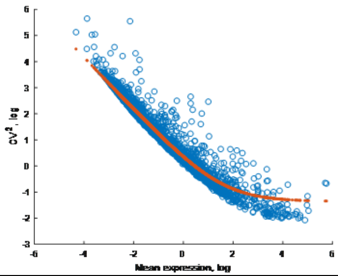
VEG	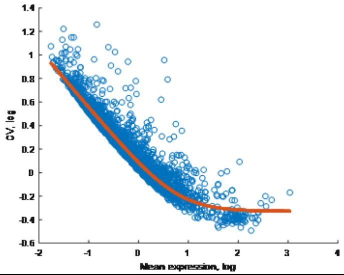	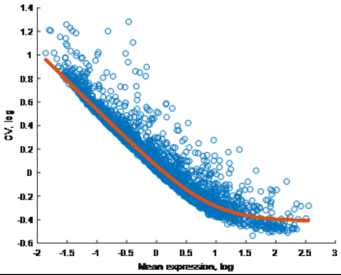	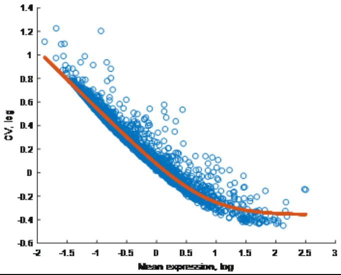

Supplementary Fig. S5. Venn diagram showing overlap between HVGs identified in three cell types.

Supplementary Fig. S5. Venn diagram showing overlap between HVGs identified in three cell types.

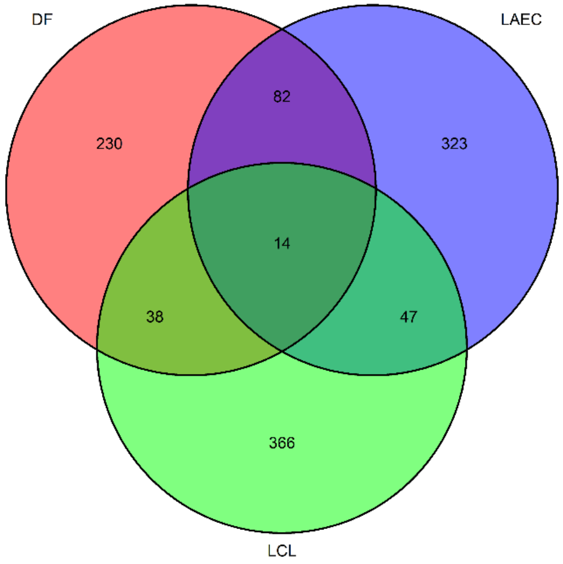

Supplementary Fig. S6. Correlation between scEV and population-level expression variability across genes of functional sets.

1-8 gene sets are defined by GO terms for biological process (BP); 9-16 molecular function (MF). Four subplots highlighted with an orange box are those also shown in the figure in the main text.

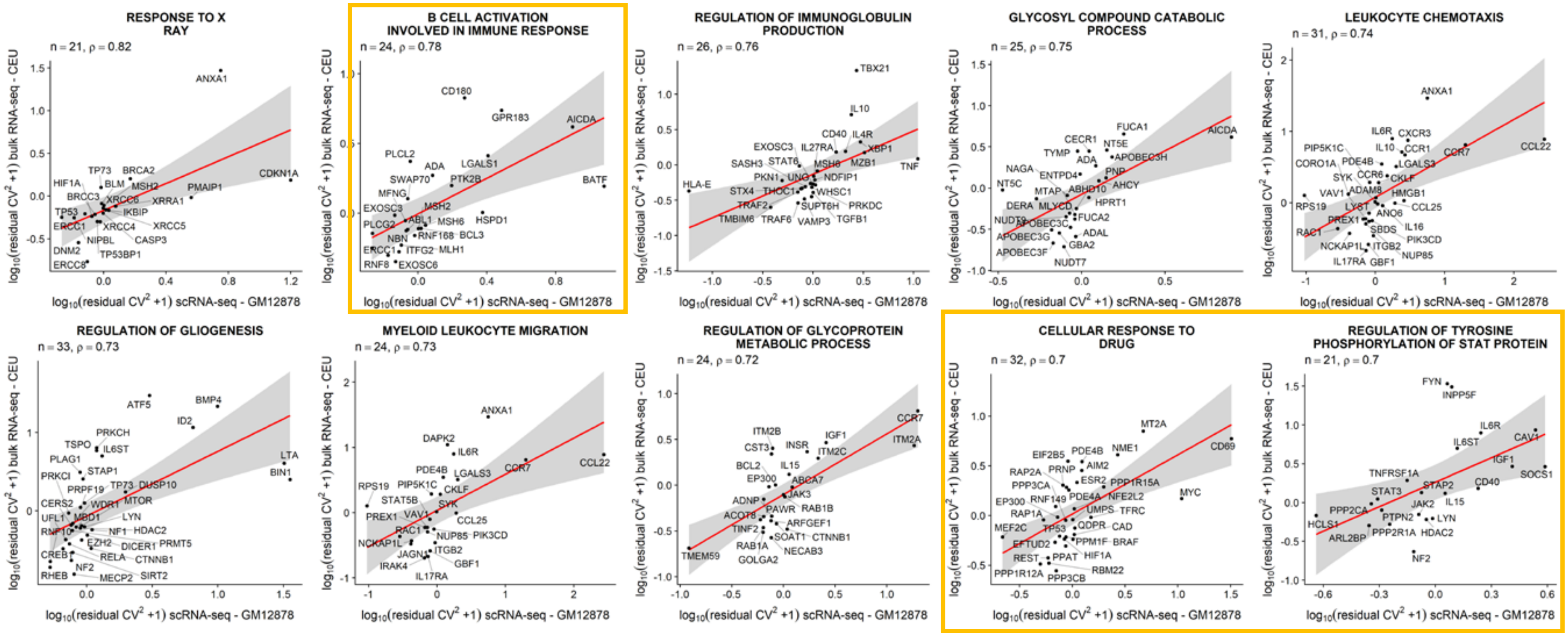

Supplementary Fig. S6. Correlation between scEV and population-level expression variability across genes of functional sets.

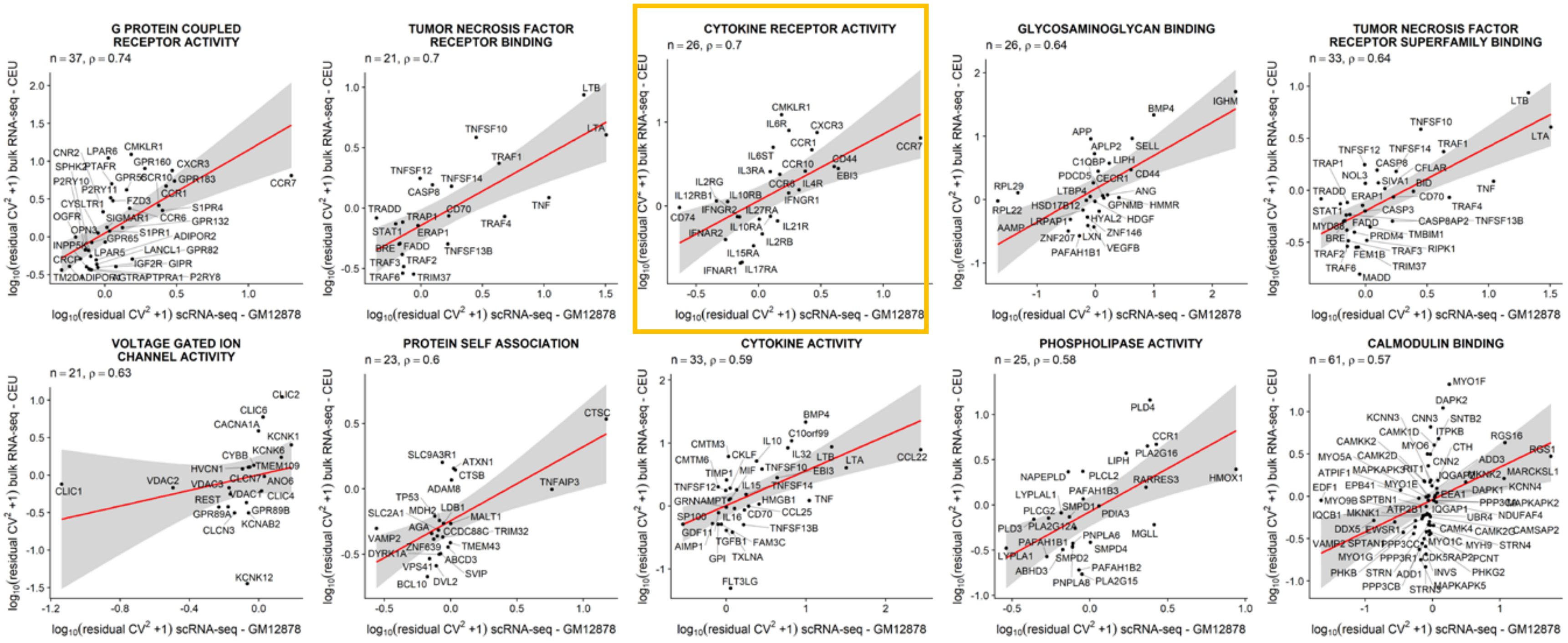

Supplementary Fig. S7. The relationship between CV2 and mean expression of genes in human iPSCs.

Supplementary Fig. S7. The relationship between CV2 and mean expression of genes in human iPSCs.

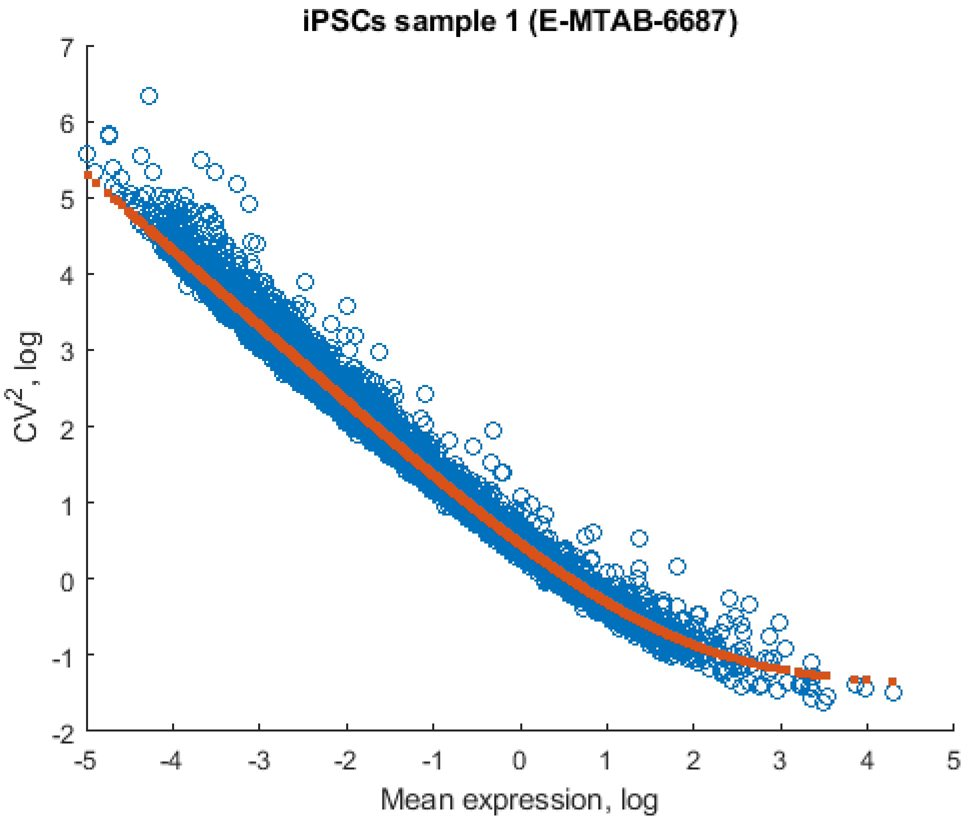

Supplementary Fig. S8. PHATE 3-D embedding plot for cells colored according to IGLC2 expression level in cells.

Supplementary Fig. S8. PHATE 3-D embedding plot for cells colored according to IGLC2 expression level in cells.

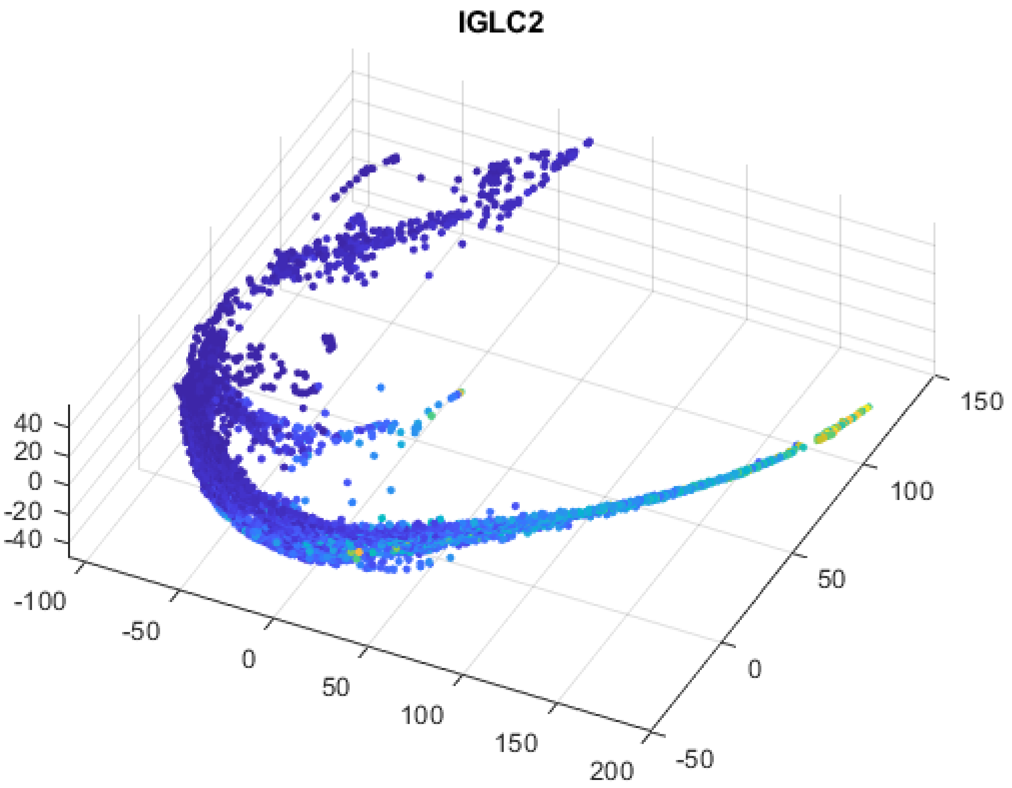

Supplement: Supplementary file 1 [file cells-09-00014-s001.zip › supplMaterial/suppl_figs.pdf]
