# Supplementary material for: Single-Cell Expression Variability Implies Cell Function
Source: Cells. 2019 Dec 19;9(1):14. doi: 10.3390/cells9010014 (PMC7017299; doi:10.3390/cells9010014)
Supplement: Supplementary file 1 [file cells-09-00014-s001.zip › supplMaterial/suppl_tables.pdf]

Single-cell expression variability implies cell function

Daniel Osorio[1,#], Xue Yu[2,#], Yan Zhong[3,#], Guanxun Li[3], Peng Yu[4], Erchin Serpedin[4], Jianhua Huang[3,*], James J. Cai[1,4,5,*]

[1]Department of Veterinary Integrative Biosciences; [2]Department of Veterinary Pathobiology; [3]Department of Statistics; [4]Department of Electrical and Computer Engineering; [5]Interdisciplinary Program of Genetics; Texas A&M University, College Station, TX 77843, USA.

[#]These authors contributed equally to this work.

[*]Corresponding authors: JH (jianhua@stat.tamu.edu) and JJC (jcai@tamu.edu)

Supplementary Tables

Supplementary Table S1. HVGs of lymphoblastoid cell line (LCL) cells (FDR<0.01). 2

Supplementary Table S2. HVGs of lung airway epithelial cells (LAECs) (FDR<0.01). 13

Supplementary Table S3. HVGs of dermal fibroblasts (DFs) (FDR<0.01). 24

Supplementary Table S4. Significantly enriched motifs found in HVGs. 33

Supplementary Table S5. HVGs of induced pluripotent stem cell (iPSCs) (FDR<0.01) 34

Supplementary Table S1. HVGs of lymphoblastoid cell line (LCL) cells (FDR<0.01).

Supplementary Table S1. HVGs of lymphoblastoid cell line (LCL) cells (FDR<0.01).

id	genes	u	cv2	residualcv2	fitratio	pval	fdr
1	IGKC	3.279132	81.83502	4.185526	65.72806	0	0
2	WFDC2	0.145432	328.9569	3.611274	37.01318	0	0
3	IGHM	3.83006	38.27298	3.467405	32.05346	0	0
4	CCL4	0.613502	76.75264	3.315936	27.54818	0	0
5	CCL22	0.336603	112.033	3.249625	25.78067	0	0
6	CCL4L2	0.248003	135.3981	3.189026	24.26478	0	0
7	IGHG1	0.879858	35.99707	2.789436	16.27184	0	0
8	RGS1	0.386765	52.73496	2.604946	13.5305	0	0
9	CCL3	2.841433	17.42626	2.595852	13.40801	0	0
10	CCL3L3	0.279217	65.97229	2.568705	13.04892	0	0
11	BIN1	0.696029	27.70819	2.381194	10.81781	0	0
12	LINC00176	0.90831	21.68926	2.30171	9.991251	0	0
13	CD69	0.548075	27.29328	2.203893	9.060216	0	0
14	CYP1B1	0.128323	83.99067	2.13275	8.438037	0	0
15	KCNMA1	0.050237	198.4927	2.110967	8.256225	0	0
16	PRSS2	0.044945	198.6256	2.004232	7.420392	0	0
17	CD7	0.031185	277.0247	1.981667	7.254825	0	0
18	IGHG3	0.637732	18.46284	1.9173	6.802569	0	0
19	ATF3	0.060945	124.9399	1.833405	6.255147	0	0
20	HIST1H1C	5.572301	6.692024	1.806432	6.088682	0	0
21	ITM2A	0.688372	14.19716	1.705278	5.502913	0	0
22	CAMP	0.198917	36.10813	1.678864	5.359464	0	0
23	DHRS9	0.18633	37.35663	1.655887	5.237722	0	0
24	CCR7	1.774538	7.845156	1.624366	5.075202	0	0
25	LTA	0.267946	26.48256	1.621888	5.062638	0	0
26	LTB	7.381157	5.143045	1.590816	4.907754	0	0
27	LINC01588	0.058874	96.39689	1.540991	4.669217	0	0
28	TNFAIP2	0.055105	102.0179	1.534266	4.637921	0	0
29	CD83	0.46288	15.67753	1.52757	4.606966	0	0
30	RGS2	0.02954	181.5317	1.506009	4.5087	0	0
31	SFN	0.042664	124.2037	1.484344	4.412069	0	0
32	MAL	0.139676	40.37007	1.477027	4.379904	0	0
33	DUSP2	1.468837	7.071851	1.436027	4.203959	0	0
34	PMCH	0.034682	137.8805	1.387602	4.005235	0	0
35	LMNA	0.693606	10.00946	1.36072	3.898999	0	0
36	ANKRD37	1.588458	6.187685	1.338556	3.813533	6.91e-323	1.55e-320
37	BMP4	0.145733	32.63714	1.30266	3.67907	1.73E-301	3.80E-299
38	CTSC	5.127163	4.096594	1.299318	3.666797	1.51E-299	3.22E-297
39	NFKBIA	2.071455	5.313563	1.297433	3.659889	1.86E-298	3.86E-296
40	CDKN1A	1.573795	5.831791	1.275124	3.579144	9.08E-286	1.84E-283

Supplementary Table S1. HVGs of lymphoblastoid cell line (LCL) cells (FDR<0.01).

41	RGS16	0.322842	16.02215	1.271473	3.5661	9.96E-284	1.96E-281
42	MYC	0.662088	9.439371	1.271459	3.566053	1.01E-283	1.96E-281
43	IER3	0.190097	24.94214	1.269428	3.558817	1.37E-282	2.58E-280
44	FSCN1	0.345521	14.96179	1.257065	3.515091	8.85E-276	1.63E-273
45	IFI27	0.069111	61.65962	1.246983	3.479827	2.60E-270	4.70E-268
46	S100A4	0.77263	8.282655	1.240495	3.457324	7.83E-267	1.38E-264
47	MIR155HG	1.409503	5.804966	1.218997	3.383793	1.57E-255	2.70E-253
48	RP11-291B21.2	0.06744	61.24653	1.217002	3.377048	1.68E-254	2.85E-252
49	MIER2	0.107068	38.38238	1.183488	3.265745	1.32E-237	2.19E-235
50	TUBB4B	0.758929	7.812086	1.170724	3.224327	2.22E-231	3.60E-229
51	HSPB1	2.934978	4.132356	1.16681	3.211732	1.70E-229	2.71E-227
52	TUBA1B	4.204364	3.690298	1.151269	3.162204	4.11E-222	6.41E-220
53	ENTPD2	0.03457	109.0478	1.149859	3.157747	1.89E-221	2.89E-219
54	RSPH1	0.050121	74.62172	1.130425	3.096971	1.80E-212	2.71E-210
55	DUSP4	0.186136	21.86224	1.119224	3.062478	2.07E-207	3.06E-205
56	TIMD4	0.727041	7.534822	1.107223	3.025944	4.43E-202	6.41E-200
57	FAM83D	0.04374	82.9143	1.104333	3.01721	8.23E-201	1.17E-198
58	IL4I1	0.302539	13.97623	1.082447	2.951895	2.24E-191	3.14E-189
59	RARRES2	0.032028	107.9551	1.065316	2.901757	3.33E-184	4.58E-182
60	PLK3	0.099306	36.12493	1.053114	2.866564	3.30E-179	4.46E-177
61	MARCKSL1	1.653733	4.523364	1.043223	2.838351	3.16E-175	4.20E-173
62	MACROD2	0.37391	11.24497	1.033409	2.81063	2.46E-171	3.21E-169
63	ATP1B1	0.067846	49.24275	1.004572	2.730739	3.06E-160	3.94E-158
64	SGK1	0.083907	40.25531	1.003899	2.728902	5.47E-160	6.94E-158
65	TNF	0.232107	16.02188	0.9988	2.715022	4.43E-158	5.53E-156
66	C10orf99	0.100097	33.59288	0.987821	2.685377	5.05E-154	6.21E-152
67	LINC01480	1.180651	5.030214	0.986607	2.682118	1.40E-153	1.70E-151
68	IGLV6-57	0.364215	10.85912	0.978009	2.659158	1.86E-150	2.22E-148
69	HMOX1	0.652307	7.096395	0.976255	2.654497	7.96E-150	9.36E-148
70	IL32	0.128028	26.16786	0.964484	2.623433	1.24E-145	1.44E-143
71	BATF	0.915177	5.665865	0.963788	2.621608	2.19E-145	2.50E-143
72	STEAP1	0.028884	106.7261	0.952881	2.59317	1.41E-141	1.59E-139
73	INSIG1	0.747742	6.33211	0.951267	2.588988	5.11E-141	5.68E-139
74	EGR2	0.103258	31.40006	0.949158	2.583534	2.73E-140	2.99E-138
75	AICDA	0.760627	6.219602	0.944171	2.570682	1.40E-138	1.51E-136
76	HMGB2	1.294716	4.568666	0.937745	2.554216	2.13E-136	2.27E-134
77	S100A10	0.217619	15.88443	0.935194	2.547709	1.54E-135	1.63E-133
78	LGALS14	0.458972	8.518501	0.911315	2.487591	1.17E-127	1.21E-125
79	NPIPB15	0.082273	36.99746	0.901016	2.462103	2.34E-124	2.40E-122
80	PRKCDBP	0.034718	83.96069	0.892571	2.441398	1.08E-121	1.10E-119
81	ANXA1	0.121821	25.18031	0.880649	2.412465	5.42E-118	5.43E-116
82	CLC	0.041854	69.08249	0.879142	2.408831	1.57E-117	1.55E-115
83	RP5-887A10.1	0.867276	5.284174	0.862083	2.368089	2.22E-112	2.17E-110

Supplementary Table S1. HVGs of lymphoblastoid cell line (LCL) cells (FDR<0.01).

84	BCL2A1	0.630299	6.444295	0.856842	2.35571	7.92E-111	7.65E-109
85	TNFRSF18	0.059105	47.90335	0.845448	2.329021	1.68E-107	1.60E-105
86	GPR34	0.070685	39.89794	0.833053	2.300332	5.88E-104	5.55E-102
87	ID1	0.034407	79.10255	0.824199	2.280053	1.80E-101	1.68E-99
88	STAG3	0.586888	6.543555	0.82344	2.278323	2.92E-101	2.69E-99
89	HES1	0.064485	42.71778	0.814068	2.25707	1.12E-98	1.02E-96
90	ZFP36L1	1.507996	3.737536	0.81063	2.249324	9.72E-98	8.76E-96
91	HEY1	0.054765	49.16239	0.798318	2.221801	1.98E-94	1.77E-92
92	NAALADL2-AS2	0.173094	16.67251	0.784356	2.190995	9.16E-91	8.08E-89
93	B4GALT1	0.985705	4.522424	0.78136	2.184442	5.45E-90	4.75E-88
94	CXCR4	0.055106	46.7748	0.754478	2.1265	3.13E-83	2.70E-81
95	STMN1	1.487363	3.543647	0.750941	2.118993	2.29E-82	1.96E-80
96	PHGDH	0.207348	13.74494	0.748943	2.114763	7.01E-82	5.92E-80
97	ID2	0.398587	8.000331	0.7423	2.100761	2.80E-80	2.34E-78
98	JUNB	1.379893	3.631655	0.739704	2.095315	1.17E-79	9.68E-78
99	T	0.017515	140.4125	0.735502	2.086529	1.16E-78	9.53E-77
100	EGR3	0.028054	86.98154	0.719775	2.05397	5.37E-75	4.36E-73
101	BIRC3	0.763761	4.931221	0.714648	2.043468	7.95E-74	6.39E-72
102	ICAM1	0.158202	16.76304	0.709931	2.03385	9.28E-73	7.38E-71
103	TCL1A	0.919032	4.37611	0.707958	2.029842	2.58E-72	2.03E-70
104	CHAC1	0.088846	28.31453	0.705677	2.025217	8.34E-72	6.51E-70
105	SOCS3	0.068952	35.86295	0.702881	2.019562	3.50E-71	2.70E-69
106	SOX4	0.364761	8.227901	0.701705	2.017189	6.38E-71	4.88E-69
107	GZMH	0.064235	38.22001	0.699103	2.011946	2.40E-70	1.82E-68
108	FRMD4A	0.388751	7.756317	0.692124	1.997954	8.07E-69	6.06E-67
109	ABCB9	0.14963	17.29215	0.691171	1.996052	1.30E-68	9.67E-67
110	CEBPD	0.083832	29.30821	0.685677	1.985115	1.99E-67	1.47E-65
111	DUSP11	0.588895	5.684222	0.685008	1.983787	2.77E-67	2.03E-65
112	NFKBID	0.131666	19.17556	0.679074	1.972052	5.09E-66	3.69E-64
113	SELL	0.612654	5.493063	0.677893	1.969724	9.04E-66	6.49E-64
114	JUN	0.274586	10.08388	0.676611	1.9672	1.69E-65	1.20E-63
115	TRAF1	0.195168	13.28034	0.662095	1.93885	1.74E-62	1.23E-60
116	CYS1	0.048282	48.38157	0.661089	1.9369	2.79E-62	1.95E-60
117	PSAT1	0.300337	9.211261	0.659581	1.933982	5.67E-62	3.93E-60
118	CHI3L2	1.043576	3.877033	0.659498	1.933822	5.89E-62	4.05E-60
119	COCH	0.276512	9.750708	0.648785	1.913214	8.44E-60	5.75E-58
120	NAALADL2	0.066898	34.9208	0.647502	1.910762	1.52E-59	1.03E-57
121	C1orf162	0.05587	41.27384	0.642583	1.901385	1.42E-58	9.53E-57
122	H2AFZ	7.158372	1.997749	0.640523	1.897474	3.60E-58	2.39E-56
123	TUBB2A	0.076712	30.10846	0.628996	1.875726	6.08E-56	4.01E-54
124	AQP3	0.178457	13.84027	0.625075	1.868386	3.38E-55	2.21E-53
125	MT2A	0.819732	4.262495	0.612959	1.845885	6.21E-53	4.03E-51
126	HERPUD1	5.862162	2.006135	0.611159	1.842567	1.33E-52	8.58E-51

Supplementary Table S1. HVGs of lymphoblastoid cell line (LCL) cells (FDR<0.01).

127	PTTG1	1.32013	3.263185	0.610943	1.842168	1.46E-52	9.33E-51
128	CCL5	0.271863	9.502606	0.608996	1.838585	3.32E-52	2.10E-50
129	CD24	0.182669	13.31199	0.606653	1.834282	8.88E-52	5.59E-50
130	TNFAIP3	0.197931	12.34135	0.600985	1.823914	9.40E-51	5.87E-49
131	VIM	6.387519	1.949397	0.59757	1.817697	3.84E-50	2.38E-48
132	GADD45A	0.25067	10.02283	0.594656	1.812408	1.27E-49	7.78E-48
133	IFIT1	0.489237	5.921364	0.594499	1.812123	1.35E-49	8.24E-48
134	VPREB3	0.216376	11.29908	0.589665	1.803384	9.60E-49	5.81E-47
135	EBI3	0.609183	4.978926	0.57575	1.778464	2.42E-46	1.45E-44
136	PHLDA3	0.258031	9.557647	0.571356	1.770667	1.34E-45	7.97E-44
137	HSPA5	8.422	1.806379	0.563253	1.756376	2.99E-44	1.77E-42
138	STXBP6	0.054501	38.78829	0.556666	1.744846	3.59E-43	2.11E-41
139	HAVCR2	0.060402	35.10514	0.555374	1.742592	5.82E-43	3.40E-41
140	MS4A7	0.164012	13.90695	0.555249	1.742375	6.09E-43	3.53E-41
141	NCALD	0.160436	14.12009	0.550849	1.734724	3.12E-42	1.80E-40
142	CDC20	0.172901	13.1933	0.549319	1.732073	5.49E-42	3.14E-40
143	PLAC8	4.792886	1.962381	0.549259	1.73197	5.61E-42	3.18E-40
144	IFIT3	0.686568	4.458841	0.545405	1.725306	2.30E-41	1.30E-39
145	LAMP5	0.312625	7.93308	0.542675	1.720604	6.21E-41	3.47E-39
146	GADD45B	0.724297	4.284333	0.540222	1.716388	1.51E-40	8.37E-39
147	ALOX5AP	0.612989	4.780059	0.539232	1.714689	2.15E-40	1.19E-38
148	TK1	0.344248	7.296137	0.53599	1.70914	6.86E-40	3.76E-38
149	C1orf54	0.065172	32.00274	0.535365	1.708072	8.58E-40	4.67E-38
150	ACY3	0.175732	12.79252	0.5328	1.703696	2.13E-39	1.15E-37
151	WDR91	0.116351	18.54178	0.532515	1.70321	2.36E-39	1.27E-37
152	IL2RA	0.024912	80.65077	0.527782	1.695169	1.25E-38	6.65E-37
153	CAV1	0.216287	10.62081	0.527405	1.694529	1.42E-38	7.54E-37
154	DERL3	1.309743	3.009852	0.526194	1.692478	2.17E-38	1.14E-36
155	FTL	105.8097	1.525237	0.525995	1.692142	2.32E-38	1.22E-36
156	PIM3	0.337883	7.316136	0.523931	1.688653	4.76E-38	2.47E-36
157	DDIT4	1.142262	3.200818	0.517052	1.677076	5.05E-37	2.61E-35
158	PRKAR1B	0.20494	10.98053	0.514306	1.672477	1.28E-36	6.59E-35
159	TRAF4	0.670467	4.379074	0.511735	1.668183	3.06E-36	1.56E-34
160	SOCS1	0.657208	4.432823	0.510691	1.666442	4.34E-36	2.20E-34
161	SLC25A4	1.979342	2.458911	0.509052	1.663713	7.51E-36	3.79E-34
162	BIRC5	0.381354	6.552188	0.508578	1.662925	8.80E-36	4.41E-34
163	ASB2	0.149661	14.35829	0.505427	1.657693	2.51E-35	1.25E-33
164	LINC00158	0.076616	26.60064	0.503946	1.65524	4.09E-35	2.02E-33
165	RP11-598F7.3	0.025176	77.81272	0.502301	1.652519	7.03E-35	3.46E-33
166	FABP5	3.002173	2.105804	0.499577	1.648024	1.71E-34	8.38E-33
167	CDKN2C	1.41632	2.791193	0.48907	1.630799	5.05E-33	2.45E-31
168	CCNB1	0.153231	13.80474	0.487236	1.627811	9.02E-33	4.36E-31
169	C16orf74	0.253173	8.923557	0.486813	1.627123	1.03E-32	4.95E-31

Supplementary Table S1. HVGs of lymphoblastoid cell line (LCL) cells (FDR<0.01).

170	RP11-166B2.1	0.106839	19.12912	0.485115	1.624362	1.76E-32	8.40E-31
171	MGST1	0.523172	5.047482	0.48316	1.62119	3.25E-32	1.54E-30
172	NINJ1	0.184756	11.64312	0.482659	1.620377	3.80E-32	1.79E-30
173	PIM1	0.096864	20.8155	0.478676	1.613936	1.31E-31	6.15E-30
174	CCR1	0.121371	16.89937	0.478481	1.613621	1.39E-31	6.50E-30
175	S100A6	1.204257	2.988829	0.476375	1.610227	2.67E-31	1.24E-29
176	UBE2S	1.144377	3.050675	0.469995	1.599986	1.86E-30	8.60E-29
177	CLIC3	0.102744	19.51465	0.468895	1.598227	2.60E-30	1.19E-28
178	LARP6	0.030293	62.67941	0.467226	1.595561	4.29E-30	1.96E-28
179	RHOB	0.037854	50.16877	0.46175	1.586849	2.19E-29	9.95E-28
180	ENDOD1	0.061682	31.27341	0.459815	1.58378	3.89E-29	1.75E-27
181	NCF2	0.171829	12.10841	0.45802	1.580941	6.58E-29	2.95E-27
182	DNASE1L3	0.110007	18.02134	0.452447	1.572155	3.32E-28	1.48E-26
183	TNFSF10	0.270406	8.142165	0.450035	1.568366	6.65E-28	2.95E-26
184	IGF1	0.239267	9.012927	0.449243	1.567126	8.35E-28	3.68E-26
185	HRASLS2	0.494781	5.025736	0.438689	1.550673	1.64E-26	7.17E-25
186	RHOV	0.022894	80.15174	0.438618	1.550564	1.67E-26	7.28E-25
187	CENPF	0.101166	19.16418	0.436409	1.547141	3.08E-26	1.33E-24
188	ETV7	0.161138	12.53508	0.43567	1.545999	3.77E-26	1.63E-24
189	LDHA	4.687084	1.760075	0.435634	1.545944	3.81E-26	1.63E-24
190	LMO2	0.073233	25.90068	0.434568	1.544296	5.11E-26	2.18E-24
191	RASGRP2	0.456649	5.29038	0.431206	1.539113	1.28E-25	5.44E-24
192	CLEC4C	0.077406	24.39077	0.426895	1.532492	4.11E-25	1.74E-23
193	GBP2	0.155324	12.82726	0.425933	1.531018	5.32E-25	2.24E-23
194	SPARC	0.119677	16.22031	0.424603	1.528984	7.59E-25	3.17E-23
195	TRAC	1.003201	3.13008	0.42336	1.527084	1.06E-24	4.40E-23
196	HHEX	0.231107	9.041972	0.423054	1.526616	1.15E-24	4.75E-23
197	EGR1	0.047577	38.59595	0.420914	1.523354	2.02E-24	8.33E-23
198	NPW	0.021841	82.43198	0.420402	1.522573	2.32E-24	9.49E-23
199	IFI30	0.112906	17.01675	0.419054	1.520522	3.30E-24	1.35E-22
200	MSL3	0.621533	4.1966	0.418454	1.51961	3.87E-24	1.57E-22
201	NLRP7	0.081491	22.99596	0.41649	1.516629	6.46E-24	2.61E-22
202	LGMN	0.183227	10.97084	0.415903	1.515739	7.53E-24	3.03E-22
203	PRCAT47	0.064778	28.55266	0.415514	1.515149	8.34E-24	3.33E-22
204	TBX21	0.128008	15.1052	0.414845	1.514136	9.92E-24	3.94E-22
205	FEZ1	0.111439	17.14611	0.414581	1.513736	1.06E-23	4.20E-22
206	P2RX5	0.689984	3.896822	0.413928	1.512748	1.26E-23	4.96E-22
207	MID1IP1	0.143308	13.59738	0.411973	1.509794	2.08E-23	8.17E-22
208	OASL	0.076402	24.23655	0.408233	1.504157	5.43E-23	2.12E-21
209	KIAA0101	1.959446	2.23137	0.40792	1.503687	5.88E-23	2.28E-21
210	GGH	0.375685	5.988059	0.406935	1.502207	7.56E-23	2.92E-21
211	ING1	0.131184	14.65497	0.40688	1.502124	7.66E-23	2.95E-21
212	NAPSA	0.824178	3.447388	0.404041	1.497865	1.57E-22	6.01E-21

Supplementary Table S1. HVGs of lymphoblastoid cell line (LCL) cells (FDR<0.01).

213	C16orf54	0.519249	4.669217	0.399882	1.491649	4.44E-22	1.69E-20
214	SSPN	0.086291	21.41606	0.399092	1.490471	5.41E-22	2.05E-20
215	ZFP36L2	1.909533	2.228711	0.396327	1.486355	1.07E-21	4.04E-20
216	MKI67	0.220156	9.101972	0.388271	1.474429	7.58E-21	2.85E-19
217	WNT10A	0.135055	14.00791	0.388119	1.474205	7.86E-21	2.94E-19
218	NETO2	0.173001	11.21829	0.387666	1.473537	8.76E-21	3.26E-19
219	H1FX	0.475053	4.916984	0.387137	1.472758	9.94E-21	3.68E-19
220	AC074289.1	0.60414	4.141616	0.385945	1.471004	1.32E-20	4.87E-19
221	MT1E	0.161488	11.89233	0.384964	1.469562	1.67E-20	6.13E-19
222	SULF2	0.45236	5.081989	0.384019	1.468173	2.09E-20	7.63E-19
223	HMX2	0.064445	27.74095	0.381772	1.464877	3.55E-20	1.29E-18
224	RRM2	0.297716	7.018564	0.38058	1.463133	4.70E-20	1.70E-18
225	CTA-384D8.34	0.765568	3.521795	0.379523	1.461587	6.02E-20	2.17E-18
226	CCDC180	0.069647	25.63462	0.376643	1.457384	1.18E-19	4.22E-18
227	CENPW	0.371408	5.849752	0.374662	1.4545	1.86E-19	6.64E-18
228	STAC3	0.182702	10.55001	0.374272	1.453932	2.03E-19	7.23E-18
229	RP11-47L3.1	0.048145	36.3838	0.373353	1.452597	2.51E-19	8.89E-18
230	H1F0	0.028551	60.4163	0.372528	1.451399	3.03E-19	1.07E-17
231	ID3	9.208026	1.473999	0.371436	1.449815	3.89E-19	1.37E-17
232	ORC6	0.283242	7.199161	0.365213	1.440821	1.58E-18	5.54E-17
233	SEPP1	0.018414	92.28358	0.365179	1.440773	1.59E-18	5.55E-17
234	S100A11	1.326346	2.540749	0.363032	1.437682	2.57E-18	8.92E-17
235	CITED2	0.286484	7.114511	0.362729	1.437247	2.75E-18	9.50E-17
236	EFNA5	0.051838	33.48108	0.361395	1.43533	3.69E-18	1.27E-16
237	P3H2	0.155521	11.99528	0.360007	1.43334	5.01E-18	1.72E-16
238	IL10	0.113198	15.98726	0.359025	1.431933	6.22E-18	2.12E-16
239	IL4R	0.138878	13.25822	0.358376	1.431003	7.17E-18	2.43E-16
240	ATF5	0.893849	3.133712	0.357613	1.429912	8.47E-18	2.86E-16
241	TPRG1	0.162031	11.53163	0.357153	1.429254	9.36E-18	3.15E-16
242	CMTM7	0.06435	27.01145	0.35371	1.424342	1.97E-17	6.61E-16
243	TXNIP	1.161736	2.689571	0.352011	1.421924	2.84E-17	9.48E-16
244	RGCC	0.297523	6.788475	0.34672	1.414421	8.73E-17	2.90E-15
245	CRIP1	1.520919	2.339655	0.346158	1.413626	9.82E-17	3.25E-15
246	CDKN3	0.244854	7.973275	0.34615	1.413615	9.84E-17	3.25E-15
247	MYL9	0.042361	39.84131	0.340424	1.405543	3.24E-16	1.06E-14
248	MAT2A	0.331387	6.162673	0.336904	1.400605	6.65E-16	2.18E-14
249	CCND2	0.465026	4.738286	0.33443	1.397144	1.10E-15	3.58E-14
250	CXCR3	1.389463	2.409114	0.332629	1.39463	1.58E-15	5.12E-14
251	SNHG15	0.35679	5.784948	0.332119	1.393919	1.75E-15	5.65E-14
252	SERPINB1	0.120226	14.70177	0.330498	1.391661	2.42E-15	7.78E-14
253	MYBL2	0.527942	4.29147	0.327373	1.387319	4.48E-15	1.44E-13
254	TROAP	0.143708	12.45624	0.326833	1.38657	4.99E-15	1.59E-13
255	SERPINB9	0.172158	10.58543	0.325285	1.384425	6.76E-15	2.15E-13

Supplementary Table S1. HVGs of lymphoblastoid cell line (LCL) cells (FDR<0.01).

256	LRRK2	0.057138	29.4105	0.325206	1.384316	6.86E-15	2.17E-13
257	PLD4	0.255021	7.527558	0.322776	1.380956	1.10E-14	3.48E-13
258	TYW3	0.937556	2.941808	0.322488	1.380558	1.16E-14	3.66E-13
259	DDIT3	0.531173	4.240014	0.319651	1.376648	2.01E-14	6.30E-13
260	GMNN	0.260644	7.36133	0.318663	1.375287	2.43E-14	7.58E-13
261	HIST1H2BJ	0.304336	6.476414	0.318061	1.374461	2.72E-14	8.47E-13
262	PLA2G16	0.188759	9.687871	0.317574	1.373791	2.99E-14	9.26E-13
263	CCDC74A	0.27521	7.01014	0.314905	1.370129	4.96E-14	1.53E-12
264	RARRES3	0.744102	3.360024	0.314475	1.36954	5.37E-14	1.65E-12
265	CLEC2B	0.103312	16.58268	0.311196	1.365056	9.92E-14	3.04E-12
266	ADAM19	0.339408	5.889239	0.310551	1.364176	1.12E-13	3.41E-12
267	CD44	1.874008	2.060936	0.310393	1.363961	1.15E-13	3.50E-12
268	TYMS	0.440555	4.798071	0.306822	1.359099	2.22E-13	6.73E-12
269	PTCRA	0.029281	54.95829	0.302529	1.353277	4.85E-13	1.46E-11
270	S1PR2	0.27225	6.984789	0.302343	1.353025	5.01E-13	1.51E-11
271	BNIP3	0.815303	3.131733	0.301359	1.351694	5.98E-13	1.79E-11
272	ZC3H12A	0.123546	13.90203	0.299475	1.34915	8.38E-13	2.50E-11
273	CD22	0.538987	4.101396	0.296772	1.345508	1.35E-12	4.02E-11
274	TUBB2B	0.025851	61.67063	0.295761	1.344149	1.62E-12	4.79E-11
275	RP3-428L16.2	0.069451	23.69647	0.295351	1.343597	1.74E-12	5.13E-11
276	ISYNA1	0.036379	44.09157	0.293997	1.341779	2.20E-12	6.47E-11
277	LINC01531	0.026813	59.3933	0.293948	1.341714	2.22E-12	6.50E-11
278	GPR183	1.552441	2.199494	0.293808	1.341526	2.28E-12	6.64E-11
279	CRELD2	5.273557	1.488956	0.29289	1.340295	2.67E-12	7.76E-11
280	NR6A1	0.07672	21.50155	0.29241	1.339652	2.90E-12	8.41E-11
281	PIM2	1.575018	2.181354	0.292098	1.339234	3.06E-12	8.85E-11
282	ABCA6	0.065909	24.75805	0.289397	1.335622	4.88E-12	1.40E-10
283	SRM	2.786531	1.743174	0.287396	1.332952	6.87E-12	1.97E-10
284	GIMAP7	0.176153	9.98532	0.287164	1.332643	7.14E-12	2.04E-10
285	SETP10	0.261747	7.103375	0.286511	1.331772	7.98E-12	2.27E-10
286	PCID2	0.417661	4.890591	0.285749	1.330758	9.08E-12	2.58E-10
287	SLC2A5	0.283454	6.644428	0.285643	1.330617	9.25E-12	2.61E-10
288	MIR3142HG	0.047236	33.90387	0.284354	1.328904	1.15E-11	3.24E-10
289	ITGB2-AS1	0.047324	33.786	0.282686	1.326689	1.52E-11	4.27E-10
290	SELM	0.604004	3.731001	0.281382	1.324959	1.89E-11	5.28E-10
291	WARS	1.051316	2.644119	0.280867	1.324278	2.06E-11	5.74E-10
292	LMTK2	0.096063	17.1918	0.279679	1.322706	2.50E-11	6.96E-10
293	RP3-395M20.12	0.066651	24.20264	0.277371	1.319655	3.66E-11	1.01E-09
294	DMKN	0.047765	33.27008	0.276236	1.318159	4.40E-11	1.22E-09
295	MKNK2	0.759758	3.183846	0.273832	1.314994	6.50E-11	1.79E-09
296	RASSF6	0.041744	37.77582	0.272963	1.313852	7.48E-11	2.05E-09
297	RASSF4	0.035527	44.17664	0.272855	1.31371	7.61E-11	2.08E-09
298	PRDM1	0.644431	3.522823	0.267813	1.307102	1.70E-10	4.62E-09

Supplementary Table S1. HVGs of lymphoblastoid cell line (LCL) cells (FDR<0.01).

299	SAMSN1	1.224011	2.404877	0.267425	1.306596	1.80E-10	4.90E-09
300	LDLRAD4	0.234995	7.62901	0.267292	1.306422	1.84E-10	4.98E-09
301	DUSP10	0.290515	6.390739	0.266887	1.305893	1.96E-10	5.29E-09
302	TRIB3	0.319711	5.90341	0.26521	1.303705	2.55E-10	6.86E-09
303	ZBTB32	0.317389	5.936587	0.264954	1.303371	2.66E-10	7.11E-09
304	LINC00926	0.254169	7.123154	0.264758	1.303115	2.74E-10	7.31E-09
305	HMGN2	7.576107	1.359058	0.263839	1.301919	3.16E-10	8.40E-09
306	PMAIP1	1.807479	1.99637	0.263558	1.301553	3.30E-10	8.75E-09
307	HNRNPH1	1.21034	2.408447	0.263094	1.300949	3.54E-10	9.36E-09
308	CD180	0.277111	6.617096	0.262887	1.30068	3.66E-10	9.64E-09
309	ZWINT	0.266005	6.842029	0.262451	1.300112	3.91E-10	1.03E-08
310	SPSB1	0.059494	26.54162	0.261247	1.298549	4.71E-10	1.23E-08
311	HMMR	0.083075	19.32586	0.260726	1.297872	5.10E-10	1.33E-08
312	MDM2	0.425232	4.698544	0.25927	1.295984	6.37E-10	1.66E-08
313	TMEM51	0.0585	26.91249	0.259011	1.295649	6.62E-10	1.72E-08
314	NKG7	0.283712	6.46387	0.258839	1.295425	6.80E-10	1.76E-08
315	PAQR7	0.052783	29.67548	0.258091	1.294457	7.61E-10	1.96E-08
316	BBC3	0.150664	11.13071	0.2568	1.292786	9.25E-10	2.38E-08
317	C1orf186	0.464827	4.374272	0.254178	1.289402	1.37E-09	3.50E-08
318	APOL1	0.350096	5.423372	0.252671	1.28746	1.71E-09	4.37E-08
319	ERN1	0.31412	5.910346	0.252187	1.286836	1.84E-09	4.68E-08
320	RP11-138A9.2	0.197457	8.722265	0.251825	1.286371	1.94E-09	4.92E-08
321	PHF19	0.452447	4.451851	0.251778	1.286311	1.95E-09	4.94E-08
322	SDF2L1	9.953629	1.292634	0.249488	1.283369	2.73E-09	6.89E-08
323	TRBC2	0.52028	4.007111	0.248381	1.281948	3.21E-09	8.06E-08
324	TBC1D30	0.02749	55.31664	0.247284	1.280543	3.76E-09	9.42E-08
325	TLCD1	0.048234	32.01589	0.24724	1.280487	3.79E-09	9.45E-08
326	GNG3	0.082051	19.28027	0.246705	1.279801	4.09E-09	1.02E-07
327	OSR2	0.07772	20.26367	0.245353	1.278073	4.97E-09	1.23E-07
328	BCAS1	0.034081	44.69735	0.244087	1.276455	5.95E-09	1.47E-07
329	FCRL5	0.188663	9.005044	0.244039	1.276394	5.99E-09	1.48E-07
330	RAB37	0.074813	20.93907	0.242121	1.273948	7.87E-09	1.93E-07
331	AC104024.1	0.07801	20.09804	0.240658	1.272086	9.67E-09	2.37E-07
332	SAMD10	0.225556	7.67493	0.23844	1.269267	1.32E-08	3.22E-07
333	CAPN10-AS1	0.180558	9.298014	0.23758	1.268177	1.48E-08	3.62E-07
334	RP6-159A1.4	0.090128	17.47114	0.236263	1.266507	1.78E-08	4.33E-07
335	IRF4	2.116037	1.822407	0.235509	1.265553	1.97E-08	4.78E-07
336	CCNB2	0.168205	9.873941	0.235156	1.265107	2.07E-08	5.01E-07
337	UNC13C	0.027744	54.12548	0.234504	1.264281	2.27E-08	5.46E-07
338	TOX2	0.047796	31.86032	0.23356	1.263089	2.58E-08	6.19E-07
339	CLDN14	0.040711	37.00644	0.228111	1.256224	5.36E-08	1.28E-06
340	RP11-511B23.1	0.022967	64.63706	0.226614	1.254346	6.53E-08	1.56E-06
341	PTP4A3	0.184372	8.988018	0.222016	1.248592	1.19E-07	2.83E-06

Supplementary Table S1. HVGs of lymphoblastoid cell line (LCL) cells (FDR<0.01).

342	LBH	0.354835	5.201236	0.221438	1.24787	1.28E-07	3.04E-06
343	C1orf228	0.076184	20.15095	0.220918	1.247221	1.37E-07	3.24E-06
344	TSC22D3	0.202342	8.261061	0.218697	1.244455	1.82E-07	4.28E-06
345	IFIT2	0.173193	9.458911	0.218059	1.243661	1.97E-07	4.63E-06
346	OAS3	0.073362	20.81269	0.217532	1.243005	2.11E-07	4.94E-06
347	A4GALT	0.129111	12.30088	0.217289	1.242704	2.17E-07	5.08E-06
348	LMO7	0.086689	17.75294	0.215826	1.240887	2.61E-07	6.08E-06
349	S1PR4	2.037246	1.81232	0.215305	1.24024	2.78E-07	6.47E-06
350	CASP1	0.076087	20.03963	0.214177	1.238842	3.20E-07	7.43E-06
351	UCHL1	0.514124	3.90098	0.213012	1.237399	3.70E-07	8.55E-06
352	RGS3	0.142748	11.18005	0.212704	1.237018	3.84E-07	8.86E-06
353	SPIB	0.862988	2.761272	0.210077	1.233774	5.30E-07	1.22E-05
354	MYH10	0.022644	64.46674	0.210037	1.233724	5.33E-07	1.22E-05
355	TNFSF4	0.019291	75.46696	0.209855	1.233499	5.45E-07	1.25E-05
356	MNDA	0.744246	3.018694	0.207475	1.230567	7.26E-07	1.66E-05
357	IPCEF1	0.10439	14.80309	0.207288	1.230337	7.43E-07	1.69E-05
358	KCNQ1OT1	0.288871	6.048929	0.207273	1.230318	7.44E-07	1.69E-05
359	RP11-1151B14.4	0.103325	14.93821	0.206872	1.229825	7.81E-07	1.76E-05
360	IER2	1.231438	2.255063	0.206223	1.229027	8.44E-07	1.90E-05
361	ZFP36	2.594001	1.643485	0.205095	1.227642	9.65E-07	2.17E-05
362	LST1	0.074144	20.3479	0.204985	1.227507	9.78E-07	2.19E-05
363	TNFSF14	0.185232	8.798172	0.204745	1.227212	1.01E-06	2.25E-05
364	RAB13	0.940303	2.610178	0.204584	1.227015	1.03E-06	2.29E-05
365	NME8	0.055862	26.63093	0.204278	1.226639	1.06E-06	2.36E-05
366	CD1C	0.02866	50.86385	0.204163	1.226498	1.08E-06	2.39E-05
367	RP4-555D20.2	0.036283	40.39424	0.203839	1.226101	1.12E-06	2.48E-05
368	CBX6	0.809157	2.848714	0.201974	1.223817	1.39E-06	3.07E-05
369	MGLL	0.690025	3.152212	0.20191	1.223738	1.40E-06	3.09E-05
370	SQLE	0.57565	3.55706	0.200513	1.222029	1.65E-06	3.62E-05
371	ARHGAP24	0.044679	32.87635	0.199825	1.221189	1.79E-06	3.91E-05
372	ANP32E	0.550862	3.6608	0.198504	1.219577	2.08E-06	4.54E-05
373	RP11-138I18.2	0.103733	14.75873	0.198437	1.219496	2.10E-06	4.57E-05
374	BCL2L1	0.221488	7.488833	0.198354	1.219394	2.12E-06	4.60E-05
375	QPRT	1.032586	2.456413	0.197231	1.218025	2.41E-06	5.21E-05
376	CCL17	0.019474	73.78878	0.196692	1.217369	2.56E-06	5.53E-05
377	IRF8	1.073633	2.4012	0.196081	1.216626	2.75E-06	5.91E-05
378	NLK	0.135637	11.51444	0.195989	1.216514	2.78E-06	5.96E-05
379	FCGR2A	0.070126	21.2508	0.195599	1.216039	2.90E-06	6.21E-05
380	NAB2	0.10616	14.40449	0.195549	1.215978	2.92E-06	6.22E-05
381	CHPF	0.418929	4.458511	0.195546	1.215975	2.92E-06	6.22E-05
382	NOP16	0.535848	3.718889	0.194731	1.214984	3.20E-06	6.80E-05
383	SH3TC1	0.149007	10.56121	0.194362	1.214536	3.34E-06	7.07E-05
384	GPT2	0.073485	20.29654	0.194003	1.214099	3.47E-06	7.34E-05

Supplementary Table S1. HVGs of lymphoblastoid cell line (LCL) cells (FDR<0.01).

385	LRRC75A	0.148419	10.59186	0.193708	1.213741	3.59E-06	7.56E-05
386	BEX5	0.16353	9.712392	0.193645	1.213666	3.61E-06	7.58E-05
387	PLG	0.071025	20.95442	0.19364	1.213659	3.62E-06	7.58E-05
388	LEUTX	0.042039	34.62616	0.192737	1.212564	4.00E-06	8.37E-05
389	C2CD2L	0.063747	23.20026	0.192637	1.212443	4.04E-06	8.44E-05
390	LIPH	0.192782	8.390735	0.19223	1.211949	4.23E-06	8.81E-05
391	CCL25	0.321597	5.46099	0.192032	1.21171	4.33E-06	8.98E-05
392	KCTD1	0.063944	23.08245	0.190493	1.209846	5.13E-06	0.000106
393	HCK	0.118925	12.90572	0.19023	1.209528	5.28E-06	0.000109
394	TCL1B	0.049975	29.21629	0.189911	1.209142	5.47E-06	0.000113
395	NGFRAP1	0.061395	23.96011	0.188992	1.208031	6.05E-06	0.000124
396	GPER1	0.028028	51.17853	0.188507	1.207446	6.38E-06	0.000131
397	UPP1	0.061379	23.93328	0.187626	1.206382	7.02E-06	0.000143
398	TNFSF11	0.024817	57.55387	0.186612	1.205159	7.83E-06	0.00016
399	BATF3	0.055038	26.52765	0.186146	1.204599	8.23E-06	0.000167
400	CCR10	1.062348	2.38981	0.185513	1.203836	8.81E-06	0.000179
401	MIAT	0.129774	11.85153	0.184733	1.202897	9.58E-06	0.000194
402	CCR6	0.109526	13.81517	0.182609	1.200345	1.20E-05	0.000242
403	RP11-111A22.1	0.086653	17.13928	0.180259	1.197527	1.54E-05	0.000309
404	GPR82	0.077213	19.08673	0.179335	1.196422	1.69E-05	0.000339
405	FAM46C	0.207171	7.779921	0.179077	1.196113	1.74E-05	0.000348
406	RP1-34B20.21	0.099667	14.99524	0.177237	1.193915	2.10E-05	0.000419
407	CLSPN	0.047436	30.32488	0.176873	1.19348	2.18E-05	0.000434
408	LAMP3	0.143473	10.7304	0.176217	1.192697	2.33E-05	0.000463
409	C3orf58	0.147515	10.45248	0.174976	1.191218	2.64E-05	0.000524
410	TRIB1	0.242509	6.761171	0.173142	1.189035	3.18E-05	0.000629
411	HIST1H2BC	0.174421	8.984401	0.172828	1.188661	3.28E-05	0.000647
412	CDCA7	0.152983	10.09441	0.17276	1.188581	3.30E-05	0.00065
413	RP11-693J15.5	0.798823	2.788458	0.172638	1.188436	3.34E-05	0.000656
414	NT5E	0.140953	10.8415	0.170534	1.185938	4.12E-05	0.000807
415	EVI2A	1.459279	2.000117	0.170032	1.185343	4.33E-05	0.000846
416	YPEL2	0.069847	20.76582	0.168725	1.183794	4.92E-05	0.000959
417	MANF	9.270245	1.201482	0.167846	1.182755	5.35E-05	0.001042
418	IGKV4-1	0.134145	11.28164	0.165541	1.180031	6.69E-05	0.001298
419	HSP90B1	10.60661	1.179469	0.165038	1.179438	7.02E-05	0.001359
420	ALDOC	0.21594	7.396479	0.164217	1.17847	7.59E-05	0.001466
421	MZB1	20.20867	1.115055	0.162384	1.176312	9.03E-05	0.001739
422	DPEP2	0.074694	19.36265	0.16234	1.17626	9.06E-05	0.001742
423	C12orf75	4.157763	1.376232	0.162258	1.176164	9.13E-05	0.001752
424	BCL7A	0.112674	13.18525	0.162064	1.175935	9.30E-05	0.00178
425	PSTPIP2	0.049371	28.7344	0.161552	1.175334	9.76E-05	0.001863
426	IL6R	0.084365	17.23815	0.160888	1.174553	0.000104	0.001977
427	EIF4EBP1	0.757814	2.847972	0.160729	1.174366	0.000105	0.002002

428	PFN2	0.093833	15.59545	0.160328	1.173895	0.000109	0.002074
429	C4orf32	0.121531	12.27862	0.160266	1.173823	0.00011	0.002081
430	RP3-325F22.5	0.149707	10.15513	0.159357	1.172757	0.00012	0.002258
431	SESN2	0.188701	8.263364	0.158263	1.171474	0.000132	0.002491
432	KCNK1	0.084263	17.21163	0.158217	1.171421	0.000133	0.002496
433	BIK	0.971004	2.444782	0.157677	1.170788	0.00014	0.002617
434	DNAJC5B	0.093662	15.56696	0.156793	1.169754	0.000151	0.00283
435	ADAMTS12	0.207518	7.592748	0.156171	1.169027	0.00016	0.002987
436	RP11-326C3.12	0.174496	8.830517	0.155932	1.168747	0.000164	0.003045
437	TLR1	0.126137	11.81517	0.15577	1.168557	0.000166	0.003078
438	LILRB4	0.218237	7.267996	0.155764	1.16855	0.000166	0.003078
439	INSR	0.296254	5.624794	0.155194	1.167885	0.000175	0.003233
440	CD3E	0.121496	12.21901	0.15514	1.167822	0.000176	0.003241
441	CD82	0.641958	3.153805	0.154583	1.167171	0.000185	0.0034
442	ANG	0.110251	13.34904	0.154374	1.166928	0.000188	0.003456
443	CENPV	0.377819	4.631045	0.154347	1.166895	0.000189	0.003457
444	IFNG-AS1	0.136641	10.96859	0.154105	1.166614	0.000193	0.003524
445	MYO1F	0.540306	3.547935	0.15354	1.165954	0.000203	0.003699
446	IGHG4	0.137777	10.87999	0.153487	1.165892	0.000204	0.003708
447	AL109761.5	0.110389	13.31301	0.152823	1.165118	0.000216	0.003924
448	LINC00886	0.049703	28.26315	0.151477	1.163551	0.000243	0.00441
449	GSTM3	0.040412	34.51904	0.15137	1.163427	0.000246	0.004441
450	KLHL6	0.088776	16.25661	0.150061	1.161905	0.000276	0.00497
451	XBP1	4.744628	1.318401	0.149345	1.161073	0.000293	0.005278
452	LINC01307	0.137883	10.82238	0.148877	1.16053	0.000305	0.005484
453	RGL4	0.038033	36.51086	0.148564	1.160167	0.000314	0.005622
454	MOXD1	0.044626	31.23002	0.147297	1.158698	0.00035	0.006255
455	PHACTR1	1.043839	2.319355	0.145857	1.157031	0.000396	0.007057
456	AUTS2	0.089533	16.05881	0.145767	1.156926	0.000399	0.007096
457	GLUL	0.148157	10.10625	0.14519	1.156259	0.000419	0.007434
458	KDM2B	0.215781	7.255553	0.144345	1.155282	0.00045	0.007966
459	GCSAM	0.785029	2.739985	0.144258	1.155182	0.000453	0.008007
460	FTH1	59.37972	1.050792	0.143899	1.154768	0.000467	0.008234
461	SORL1	0.400944	4.376459	0.143571	1.154389	0.00048	0.008445
462	HIP1	0.050498	27.60886	0.143337	1.154119	0.000489	0.008592
463	MIB2	0.215455	7.255598	0.143056	1.153794	0.000501	0.008778
464	SMYD3	0.151255	9.897882	0.142925	1.153644	0.000506	0.008854
465	GPR155	0.212498	7.334812	0.14204	1.152623	0.000545	0.009509

Supplementary Table S2. HVGs of lung airway epithelial cells (LAECs) (FDR<0.01).

id	genes	u	cv2	residualcv2	fitratio	pval	fdr
1	IGFBP5	0.3422	366.8569	4.521678	91.98979	0	0
2	COL1A2	0.402753	162.9321	3.852893	47.12919	0	0
3	POSTN	0.15356	253.3906	3.415588	30.43484	0	0
4	COL1A1	0.68134	62.38933	3.331199	27.97186	0	0
5	SPARC	0.30297	106.0357	3.171943	23.85378	0	0
6	TSLP	0.658173	43.62917	2.945928	19.02831	0	0
7	IGFL1	0.334309	75.65533	2.92221	18.5823	0	0
8	IFNL2	0.520793	50.31697	2.896921	18.11827	0	0
9	IFNL3	0.442755	52.73862	2.806557	16.55282	0	0
10	CCL2	1.451734	15.0024	2.454099	11.63595	0	0
11	IL23A	1.439021	14.56089	2.418541	11.22946	0	0
12	SERPINB4	1.837869	12.3673	2.406701	11.09729	0	0
13	S100A7	0.457983	29.3218	2.248435	9.472897	0	0
14	COL3A1	0.10598	111.0061	2.236555	9.361029	0	0
15	COL6A3	0.044814	234.9718	2.148192	8.569349	0	0
16	PI3	3.386293	6.972353	2.147977	8.56751	0	0
17	RP11-338I21.1	0.035711	286.7962	2.123833	8.363136	0	0
18	TEX26-AS1	0.030836	330.5665	2.12091	8.338725	0	0
19	OVOS2	0.1025	101.2763	2.1127	8.270542	0	0
20	GREM1	0.038824	256.9982	2.096538	8.13795	0	0
21	AMTN	0.481061	20.84226	1.948835	7.020506	0	0
22	TNFAIP6	1.47615	8.760052	1.926826	6.867676	0	0
23	S100A8	1.074926	10.35836	1.879684	6.551435	0	0
24	MSMB	1.007618	10.74332	1.869777	6.486847	0	0
25	IL17C	2.239176	6.274484	1.839883	6.295805	0	0
26	LY6D	0.186798	43.30096	1.832953	6.252323	0	0
27	SLC15A2	0.295966	28.34831	1.831725	6.244652	0	0
28	KRT81	0.094953	80.34071	1.807393	6.09454	0	0
29	AREG	9.075432	3.566721	1.799062	6.043975	0	0
30	CXCL6	0.678661	13.03051	1.761965	5.823868	0	0
31	CTGF	0.146548	50.33998	1.755226	5.784755	0	0
32	CRCT1	0.47111	17.12071	1.734429	5.665694	0	0
33	PPBP	0.758688	11.41749	1.717488	5.570517	0	0
34	MMP1	0.698346	11.95643	1.698626	5.466434	0	0
35	COL6A1	0.24869	29.02535	1.6975	5.460278	0	0
36	SERPINB2	2.356929	5.216303	1.682605	5.379553	0	0
37	SUGCT	2.210794	5.232406	1.651336	5.213943	0	0
38	S100A9	24.53591	2.468123	1.584215	4.875464	0	0
39	MEG3	0.030171	190.7004	1.549247	4.707925	0	0
40	IFNL1	0.906759	8.333716	1.538342	4.656862	0	0

Supplementary Table S2. HVGs of lung airway epithelial cells (LAECs) (FDR<0.01).

41	CXCL5	5.059604	3.155575	1.511859	4.535152	0	0
42	FBXO32	0.837573	8.458314	1.49349	4.452609	0	0
43	KRT6B	2.948961	3.709274	1.453863	4.279615	0	0
44	KRT14	0.823119	8.221108	1.451798	4.270787	0	0
45	CCL5	15.97882	2.228143	1.431141	4.183469	0	0
46	HAS2	0.0571	90.29242	1.429499	4.176607	0	0
47	ANKRD1	0.126662	41.07686	1.413192	4.10905	0	0
48	SULF1	0.046107	108.738	1.405627	4.078084	0	0
49	SERPINB3	0.841328	7.692464	1.401977	4.063225	0	0
50	CCL7	0.024243	202.5249	1.39286	4.026348	0	0
51	NEXN	0.469267	12.0642	1.381055	3.979096	0	0
52	LCE3D	0.054741	86.82733	1.34906	3.853802	0	0
53	IL20	0.066254	71.97708	1.34811	3.850142	0	0
54	THBS2	0.073408	64.69675	1.341375	3.8243	0	0
55	SBSN	0.03048	153.0234	1.339206	3.816012	2.96e-323	5.90-321
56	GDF15	2.710583	3.426464	1.333822	3.795521	5.22e-320	1.02e-317
57	COL6A2	0.031812	144.0976	1.321402	3.748673	1.56e-312	3.00-310
58	IL13RA2	0.543652	9.653596	1.281669	3.602647	1.89E-289	3.57E-287
59	LUM	0.016842	259.2768	1.278459	3.5911	1.22E-287	2.27E-285
60	VIM	0.640769	8.331497	1.268731	3.556335	3.34E-282	6.10E-280
61	CGA	0.044896	97.29511	1.26828	3.554734	5.93E-282	1.07E-279
62	DKK1	0.303146	15.37342	1.241329	3.460209	2.81E-267	4.92E-265
63	ADAMTS2	0.015892	264.6734	1.241323	3.46019	2.83E-267	4.92E-265
64	CCL3L3	0.036405	116.2132	1.239485	3.453833	2.71E-266	4.64E-264
65	IFNB1	0.075163	56.06027	1.221074	3.390829	1.31E-256	2.21E-254
66	CSF2	3.525373	2.669806	1.205479	3.338359	1.34E-248	2.22E-246
67	SFTA1P	1.283625	4.638519	1.199123	3.317207	2.19E-245	3.58E-243
68	TIMP1	9.432114	1.940592	1.198977	3.316721	2.59E-245	4.18E-243
69	OLR1	0.427302	10.84251	1.194176	3.300838	6.62E-243	1.05E-240
70	RGS2	0.769233	6.588071	1.17831	3.24888	4.57E-235	7.16E-233
71	ITGA1	0.17072	24.20949	1.167215	3.213031	1.09E-229	1.68E-227
72	FST	7.060735	1.9896	1.15296	3.167554	6.59E-223	1.00E-220
73	DHRS9	0.730404	6.605607	1.140614	3.128688	3.80E-217	5.71E-215
74	CYP24A1	0.510087	8.833089	1.1397	3.12583	1.00E-216	1.49E-214
75	TAGLN	4.963836	2.143319	1.118444	3.060088	4.64E-207	6.78E-205
76	PTX3	0.295949	13.82639	1.113685	3.045559	6.14E-205	8.86E-203
77	MMP13	0.205037	19.29789	1.111514	3.038956	5.64E-204	8.03E-202
78	AQP3	4.433787	2.187756	1.098256	2.998931	3.69E-198	5.18E-196
79	CLNK	0.291392	13.62221	1.08484	2.958968	2.16E-192	3.00E-190
80	IL6	6.026135	1.911218	1.067052	2.906797	6.38E-185	8.74E-183
81	LTB	11.41982	1.609612	1.050832	2.860031	2.77E-178	3.74E-176
82	SRGN	0.380583	10.37734	1.049867	2.857271	6.79E-178	9.07E-176
83	SPP1	0.068844	50.73216	1.03572	2.817133	3.02E-172	3.98E-170

Supplementary Table S2. HVGs of lung airway epithelial cells (LAECs) (FDR<0.01).

84	NUPR1	16.61788	1.452156	1.008497	2.741476	1.01E-161	1.32E-159
85	CYP1B1	0.107756	31.41651	0.990274	2.691971	6.35E-155	8.20E-153
86	IL36G	9.119815	1.584866	0.989014	2.688581	1.84E-154	2.35E-152
87	MMP9	0.212473	16.50988	0.988497	2.687194	2.85E-154	3.59E-152
88	IL1RL1	0.235331	14.97132	0.984882	2.677495	5.99E-153	7.46E-151
89	COL4A1	0.422932	8.838681	0.980983	2.667077	1.56E-151	1.93E-149
90	DUOXA2	3.124207	2.233415	0.973434	2.647019	8.19E-149	9.97E-147
91	CXCL9	0.067621	48.11255	0.965227	2.625383	6.79E-146	8.18E-144
92	KRT23	0.507769	7.444874	0.964902	2.62453	8.85E-146	1.05E-143
93	PLAT	1.240323	3.724778	0.956505	2.602585	7.79E-143	9.18E-141
94	RRAD	0.062065	51.77931	0.954983	2.598627	2.64E-142	3.08E-140
95	SPRR2D	0.108205	29.41605	0.928475	2.530648	2.73E-133	3.15E-131
96	LINC00958	2.04908	2.648251	0.92828	2.530155	3.17E-133	3.62E-131
97	NKX3-1	1.471711	3.226156	0.925982	2.524346	1.84E-132	2.08E-130
98	INHBA	6.242686	1.619937	0.912333	2.490125	5.46E-128	6.10E-126
99	CYR61	1.479118	3.165918	0.910356	2.485206	2.38E-127	2.63E-125
100	CCNA1	1.037619	3.977149	0.897215	2.452762	3.74E-123	4.11E-121
101	CKS2	6.927309	1.547587	0.896489	2.450983	6.35E-123	6.89E-121
102	STC1	1.893739	2.680656	0.895264	2.447983	1.54E-122	1.66E-120
103	BAMBI	0.125974	24.58496	0.894682	2.446557	2.35E-122	2.51E-120
104	CLEC2D	0.634284	5.727787	0.885821	2.424974	1.37E-119	1.45E-117
105	CX3CL1	1.06441	3.850671	0.883138	2.418477	9.28E-119	9.69E-117
106	IL19	0.051476	56.77675	0.863981	2.372587	6.04E-113	6.24E-111
107	SPRR1B	0.552169	6.270775	0.863115	2.370534	1.09E-112	1.12E-110
108	NREP	0.178627	17.03359	0.85813	2.358746	3.30E-111	3.35E-109
109	CLEC2B	0.493746	6.846158	0.857518	2.357302	5.01E-111	5.04E-109
110	KRTAP2-3	0.110306	26.6673	0.848835	2.336924	1.75E-108	1.74E-106
111	CTD-2017D11.1	0.16016	18.31027	0.827838	2.288367	1.73E-102	1.71E-100
112	CH25H	0.063969	44.2392	0.827112	2.286706	2.76E-102	2.70E-100
113	TNF	0.364563	8.612859	0.825805	2.283719	6.41E-102	6.22E-100
114	S100A4	0.937216	3.982929	0.824546	2.280846	1.44E-101	1.38E-99
115	PTHLH	0.720085	4.866944	0.823995	2.279589	2.05E-101	1.95E-99
116	AKAP12	1.428491	2.966611	0.82286	2.277003	4.24E-101	4.00E-99
117	ALDH3A1	0.461551	6.911097	0.809842	2.247553	1.59E-97	1.49E-95
118	CCL20	46.66523	1.083928	0.808579	2.244715	3.50E-97	3.25E-95
119	LY6K	0.453381	7.003775	0.807938	2.243277	5.22E-97	4.81E-95
120	FN1	11.32735	1.24032	0.788674	2.200478	6.89E-92	6.30E-90
121	CCDC71L	2.827852	1.944631	0.788055	2.199115	1.00E-91	9.06E-90
122	FBN1	0.029648	90.62215	0.787948	2.198881	1.07E-91	9.58E-90
123	AKR1C2	0.315185	9.380832	0.782226	2.186333	3.26E-90	2.90E-88
124	KRT80	0.092043	29.52914	0.776431	2.173701	1.00E-88	8.86E-87
125	APOE	1.117765	3.340811	0.77573	2.172178	1.51E-88	1.33E-86
126	CXCL3	17.61087	1.137975	0.772467	2.165101	1.02E-87	8.90E-86

Supplementary Table S2. HVGs of lung airway epithelial cells (LAECs) (FDR<0.01).

127	SDC2	0.055933	47.57607	0.768567	2.156673	9.89E-87	8.53E-85
128	CLIC3	0.029454	88.71605	0.760192	2.138687	1.22E-84	1.05E-82
129	EREG	1.559601	2.614265	0.752548	2.122402	9.28E-83	7.89E-81
130	IL33	7.242763	1.309399	0.741465	2.099008	4.44E-80	3.74E-78
131	C8orf4	0.25284	10.90537	0.733702	2.082776	3.10E-78	2.59E-76
132	FLJ22447	0.059563	43.1313	0.732023	2.079284	7.69E-78	6.38E-76
133	KIAA0101	0.498177	5.96862	0.727869	2.070663	7.21E-77	5.94E-75
134	SLC2A3	0.041903	60.03886	0.717636	2.049583	1.66E-74	1.36E-72
135	PTGS2	1.72444	2.358165	0.711458	2.036959	4.20E-73	3.41E-71
136	ANGPTL4	1.884057	2.196767	0.6932	2.000106	4.71E-69	3.79E-67
137	CLDN11	0.192502	13.31843	0.681992	1.977813	1.22E-66	9.78E-65
138	EDN1	3.563444	1.574349	0.681905	1.977641	1.27E-66	1.01E-64
139	EGR1	1.149755	2.981462	0.68168	1.977197	1.42E-66	1.12E-64
140	CDH11	0.062147	39.06004	0.674382	1.962819	4.96E-65	3.88E-63
141	ADAMTS1	0.855461	3.668829	0.67422	1.962502	5.36E-65	4.17E-63
142	TESC	0.072753	33.2997	0.668483	1.951275	8.42E-64	6.50E-62
143	NR4A3	0.12814	19.27864	0.667812	1.949966	1.16E-63	8.89E-62
144	RP11-357H14.17	0.032716	72.71821	0.665186	1.944852	4.04E-63	3.07E-61
145	CCK	0.317478	8.259602	0.661407	1.937517	2.41E-62	1.82E-60
146	RBP1	1.219065	2.785477	0.654117	1.923443	7.23E-61	5.43E-59
147	SERPINE1	0.54234	5.16081	0.653428	1.922119	9.95E-61	7.42E-59
148	FOLR1	0.437277	6.150304	0.647036	1.909872	1.88E-59	1.39E-57
149	SLC6A14	3.244538	1.577538	0.642914	1.902016	1.22E-58	9.00E-57
150	TNIP3	0.877111	3.467474	0.6366	1.890044	2.09E-57	1.53E-55
151	CTSC	5.947212	1.245266	0.634621	1.886307	5.05E-57	3.67E-55
152	TGM2	6.803	1.196253	0.633951	1.885043	6.81E-57	4.91E-55
153	FGFBP1	0.674075	4.239445	0.633704	1.884578	7.60E-57	5.44E-55
154	COL5A1	0.086037	27.26816	0.631485	1.880401	2.03E-56	1.44E-54
155	MAP1B	0.064541	36.02835	0.630511	1.878571	3.12E-56	2.21E-54
156	CTHRC1	0.049044	46.89721	0.625302	1.868811	3.06E-55	2.15E-53
157	SERPINB7	2.149317	1.901199	0.623492	1.865431	6.74E-55	4.70E-53
158	SLC34A2	0.422726	6.168513	0.620883	1.86057	2.09E-54	1.45E-52
159	MMP7	0.362101	7.057947	0.620746	1.860316	2.21E-54	1.53E-52
160	BPGM	3.264412	1.526346	0.612663	1.845339	7.05E-53	4.83E-51
161	CD55	1.280787	2.581217	0.611498	1.843191	1.15E-52	7.86E-51
162	MT1E	16.43354	0.971858	0.605363	1.831917	1.52E-51	1.03E-49
163	C1orf56	2.449275	1.737199	0.603226	1.828007	3.71E-51	2.50E-49
164	MYL9	2.717297	1.647673	0.602878	1.82737	4.29E-51	2.87E-49
165	AC004791.2	0.051829	43.32719	0.600334	1.822728	1.23E-50	8.18E-49
166	THBS1	1.939852	1.965333	0.598813	1.819956	2.31E-50	1.52E-48
167	KRBOX1	0.038551	57.80122	0.597541	1.817643	3.89E-50	2.55E-48
168	MROH8	0.073895	30.49126	0.595539	1.814009	8.83E-50	5.76E-48
169	ESM1	0.039579	55.92396	0.590442	1.804786	7.01E-49	4.55E-47

Supplementary Table S2. HVGs of lung airway epithelial cells (LAECs) (FDR<0.01).

170	TNFSF15	0.200596	11.69562	0.590394	1.8047	7.15E-49	4.61E-47
171	S100P	2.163384	1.829495	0.58864	1.801537	1.45E-48	9.30E-47
172	RHCG	0.67339	4.0092	0.577053	1.780783	1.45E-46	9.24E-45
173	TNFAIP2	9.340519	1.041381	0.574387	1.776041	4.11E-46	2.61E-44
174	WNT5A	0.051098	42.71092	0.572079	1.771947	1.01E-45	6.36E-44
175	AIM2	0.853244	3.310872	0.569598	1.767557	2.63E-45	1.65E-43
176	CKB	0.787468	3.50894	0.566464	1.762026	8.79E-45	5.47E-43
177	TIMP3	2.067739	1.834166	0.566068	1.761327	1.02E-44	6.33E-43
178	LAYN	0.783748	3.511114	0.563433	1.756694	2.79E-44	1.72E-42
179	ARL4C	1.175102	2.604544	0.561672	1.753602	5.45E-44	3.34E-42
180	NR4A2	0.332347	7.151168	0.558069	1.747295	2.12E-43	1.29E-41
181	TUBA1A	0.788711	3.455106	0.552218	1.737102	1.88E-42	1.14E-40
182	NR2F2	2.114002	1.781414	0.549242	1.73194	5.65E-42	3.40E-40
183	ADAMTS9	0.263164	8.721966	0.546755	1.727638	1.41E-41	8.42E-40
184	AQP5	0.063895	33.43544	0.545999	1.726331	1.85E-41	1.10E-39
185	FAM43A	0.295108	7.855156	0.545716	1.725843	2.06E-41	1.22E-39
186	SLC7A11	0.757805	3.53998	0.545557	1.725569	2.18E-41	1.28E-39
187	ATP2B1	1.462482	2.212451	0.544745	1.724169	2.93E-41	1.72E-39
188	GAL	2.522055	1.612636	0.543916	1.72274	3.96E-41	2.31E-39
189	LGALSL	0.782529	3.421232	0.5363	1.70967	6.15E-40	3.56E-38
190	EPB41L3	0.019131	108.645	0.535203	1.707796	9.08E-40	5.24E-38
191	KCNJ2	0.058154	35.67521	0.518815	1.680035	2.77E-37	1.59E-35
192	COL5A2	0.231167	9.519108	0.515639	1.674709	8.17E-37	4.67E-35
193	NR2F1	0.037995	54.00307	0.515252	1.674059	9.32E-37	5.29E-35
194	UCHL1	0.972363	2.83803	0.512688	1.669773	2.22E-36	1.25E-34
195	NPW	0.225273	9.716505	0.512389	1.669274	2.45E-36	1.38E-34
196	IGF2	0.063136	32.59572	0.508889	1.663442	7.93E-36	4.44E-34
197	HIST1H2AC	1.538272	2.060375	0.50577	1.658262	2.24E-35	1.25E-33
198	DDIT3	0.988941	2.778773	0.503919	1.655196	4.13E-35	2.28E-33
199	MT1X	8.212007	0.998223	0.502192	1.652339	7.28E-35	4.01E-33
200	VCAN	0.29549	7.493307	0.499719	1.648258	1.64E-34	8.97E-33
201	ZNF778	0.139839	14.97458	0.498324	1.645961	2.58E-34	1.41E-32
202	LOX	0.070606	28.91498	0.498134	1.645648	2.74E-34	1.49E-32
203	LIF	2.178442	1.658488	0.494316	1.639376	9.43E-34	5.09E-32
204	NUMBL	0.138755	14.98382	0.491547	1.634843	2.29E-33	1.23E-31
205	ELF3	2.404905	1.564647	0.48908	1.630816	5.03E-33	2.69E-31
206	SERPINB1	10.10383	0.939698	0.48845	1.629789	6.14E-33	3.27E-31
207	ID2	0.272889	7.950052	0.487036	1.627485	9.61E-33	5.09E-31
208	SCD	0.230175	9.28196	0.486457	1.626544	1.15E-32	6.08E-31
209	TFPI	0.752566	3.33136	0.47942	1.615137	1.04E-31	5.46E-30
210	GJB2	5.533565	1.086293	0.475298	1.608493	3.71E-31	1.94E-29
211	TPM2	4.692049	1.149045	0.475088	1.608155	3.96E-31	2.06E-29
212	CYGB	0.343181	6.380953	0.472557	1.604091	8.57E-31	4.43E-29

Supplementary Table S2. HVGs of lung airway epithelial cells (LAECs) (FDR<0.01).

213	LAMA4	0.028931	67.51106	0.469353	1.598959	2.26E-30	1.17E-28
214	GJB6	0.139544	14.5656	0.468626	1.597797	2.82E-30	1.44E-28
215	EMP3	1.770688	1.813282	0.464647	1.591453	9.29E-30	4.73E-28
216	RP11-297N6.4	0.049143	39.71304	0.461021	1.585692	2.72E-29	1.38E-27
217	EFNA1	1.658897	1.870167	0.455969	1.577702	1.20E-28	6.05E-27
218	GATA3	0.048338	39.90209	0.449554	1.567612	7.64E-28	3.84E-26
219	IGFBP2	8.149336	0.948242	0.448954	1.566673	9.06E-28	4.54E-26
220	SLPI	3.186187	1.300741	0.441794	1.555495	6.88E-27	3.43E-25
221	RARRES3	7.609456	0.951232	0.434871	1.544764	4.70E-26	2.33E-24
222	AKR1C1	0.762154	3.150766	0.433526	1.542688	6.79E-26	3.35E-24
223	NCCRP1	0.055646	34.19336	0.433228	1.542228	7.37E-26	3.62E-24
224	IRG1	0.07257	26.3601	0.432336	1.540853	9.41E-26	4.60E-24
225	SERPINB5	1.063493	2.453899	0.431955	1.540265	1.04E-25	5.09E-24
226	SERPINA1	0.475836	4.614346	0.431777	1.539991	1.10E-25	5.32E-24
227	NCF2	1.593752	1.866297	0.429091	1.535861	2.27E-25	1.10E-23
228	BIRC3	6.481638	0.985544	0.426414	1.531755	4.67E-25	2.25E-23
229	CNKSR3	0.178748	11.05336	0.426311	1.531598	4.80E-25	2.30E-23
230	CLMP	0.267812	7.605456	0.425685	1.530638	5.68E-25	2.71E-23
231	POU2F2	2.582179	1.410221	0.421781	1.524675	1.61E-24	7.63E-23
232	ATF3	0.253987	7.948156	0.421518	1.524274	1.72E-24	8.15E-23
233	SYT8	0.173626	11.29342	0.42054	1.522784	2.23E-24	1.05E-22
234	KRT6A	11.94816	0.849444	0.420014	1.521983	2.56E-24	1.20E-22
235	SPRR2A	0.02985	62.28018	0.41962	1.521383	2.85E-24	1.33E-22
236	G0S2	151.6594	0.706781	0.418782	1.520109	3.55E-24	1.65E-22
237	ZFP36	1.36719	2.033134	0.416323	1.516376	6.75E-24	3.12E-22
238	FILIP1L	0.313922	6.51299	0.413768	1.512505	1.31E-23	6.04E-22
239	CDKN2A	2.438334	1.437392	0.411455	1.509012	2.38E-23	1.09E-21
240	C1orf68	0.043623	42.41633	0.409769	1.50647	3.67E-23	1.68E-21
241	CA2	0.388176	5.372408	0.408766	1.50496	4.74E-23	2.16E-21
242	BEX1	0.021307	85.83769	0.406497	1.501548	8.45E-23	3.83E-21
243	SERPINE2	0.46447	4.558695	0.399116	1.490506	5.37E-22	2.42E-20
244	LRRC75A	0.865917	2.760278	0.398844	1.490102	5.75E-22	2.58E-20
245	PNRC1	5.125812	1.031089	0.397746	1.488466	7.54E-22	3.37E-20
246	NDRG4	0.035654	51.12058	0.397658	1.488335	7.71E-22	3.44E-20
247	S100A12	0.071562	25.74158	0.394986	1.484364	1.49E-21	6.60E-20
248	MMP10	0.040301	45.17012	0.394701	1.48394	1.59E-21	7.05E-20
249	LINC01436	0.118083	15.8465	0.393663	1.482402	2.05E-21	9.05E-20
250	PELI1	1.324096	2.030156	0.393632	1.482355	2.07E-21	9.08E-20
251	DUSP1	2.609113	1.358562	0.389698	1.476535	5.38E-21	2.35E-19
252	KCNK5	0.075029	24.45176	0.389616	1.476414	5.48E-21	2.39E-19
253	MT1M	0.048469	37.44835	0.388729	1.475105	6.79E-21	2.94E-19
254	NEDD9	0.297969	6.61253	0.382188	1.465487	3.22E-20	1.39E-18
255	CTC-425F1.4	0.220765	8.682703	0.381254	1.46412	4.01E-20	1.72E-18

256	IGFBP3	7.798741	0.894954	0.380164	1.462524	5.18E-20	2.22E-18
257	NEURL3	1.28723	2.040229	0.379677	1.461813	5.80E-20	2.48E-18
258	ASB2	1.115353	2.250322	0.379071	1.460927	6.69E-20	2.84E-18
259	PRDM8	0.467133	4.43531	0.376537	1.45723	1.20E-19	5.10E-18
260	SLC44A4	0.3689	5.429445	0.374771	1.454658	1.81E-19	7.64E-18
261	HRASLS2	0.341377	5.798069	0.372097	1.450773	3.34E-19	1.40E-17
262	ZFP36L2	1.156332	2.178909	0.372063	1.450724	3.37E-19	1.41E-17
263	TNNT1	0.305202	6.392372	0.369855	1.447525	5.57E-19	2.32E-17
264	BMP2	0.740948	3.01978	0.369092	1.44642	6.62E-19	2.75E-17
265	SNAPC1	2.35789	1.397541	0.365746	1.441589	1.41E-18	5.80E-17
266	TPM1	4.607126	1.036865	0.365736	1.441575	1.41E-18	5.80E-17
267	ADIRF	3.889909	1.106156	0.365357	1.441028	1.53E-18	6.29E-17
268	MAP3K8	0.830245	2.738063	0.358911	1.431769	6.38E-18	2.61E-16
269	CSF1	0.966038	2.443224	0.358121	1.430638	7.58E-18	3.09E-16
270	CHST2	0.353214	5.544193	0.357455	1.429686	8.77E-18	3.56E-16
271	MSC	0.070821	24.98791	0.355123	1.426356	1.45E-17	5.88E-16
272	MRGPRX3	0.28048	6.796186	0.355058	1.426263	1.47E-17	5.94E-16
273	IL20RB	0.263512	7.189783	0.354772	1.425855	1.57E-17	6.30E-16
274	HMGB3	2.201927	1.43393	0.354694	1.425744	1.60E-17	6.38E-16
275	CXCL1	33.92938	0.701017	0.352835	1.423097	2.38E-17	9.49E-16
276	SERPINB13	0.059137	29.68804	0.351496	1.421192	3.17E-17	1.26E-15
277	TAGLN3	0.434703	4.592979	0.349995	1.419061	4.37E-17	1.73E-15
278	SNPH	0.2158	8.58522	0.348953	1.417583	5.45E-17	2.15E-15
279	CHAC1	0.147935	12.22514	0.348841	1.417424	5.58E-17	2.19E-15
280	CITED2	0.94815	2.450936	0.347549	1.415594	7.33E-17	2.87E-15
281	SLC39A8	2.129204	1.438423	0.339357	1.404045	4.03E-16	1.57E-14
282	DSG3	0.191989	9.434106	0.334688	1.397504	1.04E-15	4.05E-14
283	INSIG1	0.046571	36.8735	0.334031	1.396586	1.19E-15	4.61E-14
284	TM4SF1	0.463672	4.271415	0.332562	1.394537	1.60E-15	6.17E-14
285	PNMA2	0.108412	16.17942	0.332506	1.394458	1.62E-15	6.22E-14
286	PLA2G4D	0.071294	24.1807	0.32877	1.389258	3.40E-15	1.30E-13
287	SNCG	0.912039	2.46822	0.32585	1.385207	6.05E-15	2.31E-13
288	KCCAT211	0.778525	2.776122	0.323394	1.38181	9.77E-15	3.72E-13
289	CAMK2N1	2.388932	1.326909	0.320777	1.378198	1.62E-14	6.14E-13
290	C11orf96	0.039416	42.87329	0.320648	1.37802	1.66E-14	6.28E-13
291	GK	0.127195	13.68867	0.318317	1.374812	2.59E-14	9.77E-13
292	ID3	3.980683	1.045515	0.318246	1.374715	2.63E-14	9.87E-13
293	PPP1R14A	0.427204	4.510392	0.316889	1.37285	3.41E-14	1.27E-12
294	ALPL	0.083571	20.39646	0.312952	1.367456	7.15E-14	2.67E-12
295	CD200	0.184276	9.583786	0.312147	1.366356	8.31E-14	3.09E-12
296	TK1	0.1427	12.1853	0.311427	1.365372	9.51E-14	3.52E-12
297	GS1-124K5.4	0.084753	20.07528	0.310693	1.36437	1.09E-13	4.02E-12
298	PTGER4	0.340551	5.45887	0.309669	1.362974	1.32E-13	4.85E-12

Supplementary Table S2. HVGs of lung airway epithelial cells (LAECs) (FDR<0.01).

299	KRT16	0.113131	15.13806	0.306877	1.359174	2.20E-13	8.07E-12
300	EGFLAM	0.049386	33.84237	0.305886	1.357827	2.64E-13	9.64E-12
301	ERRFI1	2.50938	1.272827	0.304705	1.356225	3.27E-13	1.19E-11
302	ALMS1	0.113365	15.06028	0.303709	1.354874	3.92E-13	1.42E-11
303	DUSP4	2.46182	1.283288	0.303027	1.353951	4.43E-13	1.60E-11
304	TLR2	0.198269	8.818481	0.297181	1.346059	1.26E-12	4.54E-11
305	TRAF1	0.15672	10.99093	0.296964	1.345767	1.31E-12	4.70E-11
306	ICAM1	5.886176	0.891101	0.296788	1.34553	1.35E-12	4.83E-11
307	NPPC	0.037736	43.69501	0.296684	1.34539	1.37E-12	4.91E-11
308	CRYAB	0.051442	32.20685	0.296373	1.344972	1.45E-12	5.17E-11
309	PVRL4	0.279204	6.425056	0.294778	1.342828	1.92E-12	6.81E-11
310	CD70	0.038632	42.50302	0.292168	1.339328	3.03E-12	1.07E-10
311	ADAMTS14	0.030981	52.83218	0.29186	1.338915	3.19E-12	1.13E-10
312	GPX3	0.058662	28.1882	0.291769	1.338794	3.24E-12	1.14E-10
313	ST3GAL4-AS1	0.05928	27.7986	0.288104	1.333896	6.09E-12	2.13E-10
314	FOS	0.241338	7.287793	0.288067	1.333846	6.13E-12	2.14E-10
315	KLK6	0.118569	14.18307	0.286688	1.332009	7.75E-12	2.70E-10
316	RRP12	0.483735	3.93246	0.285806	1.330835	8.99E-12	3.12E-10
317	SAA2	5.195694	0.916728	0.284774	1.329461	1.07E-11	3.70E-10
318	MAFB	0.065628	24.942	0.279056	1.321881	2.78E-11	9.57E-10
319	TRIB3	0.363744	4.994575	0.278923	1.321706	2.84E-11	9.75E-10
320	HLA-DPA1	0.069501	23.55107	0.277581	1.319933	3.54E-11	1.21E-09
321	HIST1H2BD	0.858356	2.455664	0.275297	1.316922	5.13E-11	1.75E-09
322	PRKCDBP	0.330595	5.41177	0.274674	1.316102	5.68E-11	1.93E-09
323	STC2	0.629737	3.120475	0.27267	1.313467	7.84E-11	2.66E-09
324	BDNF	0.326558	5.457673	0.272192	1.31284	8.46E-11	2.86E-09
325	WNT5B	0.033403	48.04385	0.271225	1.31157	9.88E-11	3.33E-09
326	ANKRD37	0.380366	4.761479	0.270302	1.31036	1.14E-10	3.85E-09
327	LGALS7B	0.17042	9.885979	0.269934	1.309878	1.21E-10	4.07E-09
328	ITGB8	0.502394	3.748529	0.269805	1.309709	1.24E-10	4.14E-09
329	RARRES1	0.400649	4.546977	0.269475	1.309277	1.31E-10	4.35E-09
330	PRR15	0.363436	4.946073	0.268421	1.307898	1.54E-10	5.12E-09
331	HIST1H4H	0.082653	19.68727	0.266863	1.305861	1.97E-10	6.53E-09
332	AF127936.7	0.464304	3.993267	0.266384	1.305237	2.12E-10	7.01E-09
333	TRBC2	0.116216	14.13743	0.264278	1.30249	2.95E-10	9.71E-09
334	HLA-G	0.983843	2.193368	0.263584	1.301586	3.28E-10	1.08E-08
335	FLRT3	0.161829	10.28549	0.260884	1.298078	4.98E-10	1.63E-08
336	GBP5	0.731093	2.733533	0.259028	1.29567	6.60E-10	2.15E-08
337	ICAM2	0.118063	13.83926	0.258062	1.29442	7.65E-10	2.49E-08
338	CST6	7.528953	0.797232	0.255505	1.291114	1.12E-09	3.64E-08
339	DUOX2	1.624878	1.54823	0.254271	1.289522	1.35E-09	4.37E-08
340	APLN	0.105928	15.29065	0.253736	1.288831	1.46E-09	4.72E-08
341	TGFBI	4.53309	0.931167	0.25228	1.286956	1.81E-09	5.83E-08

Supplementary Table S2. HVGs of lung airway epithelial cells (LAECs) (FDR<0.01).

342	IDO1	2.363911	1.243936	0.25066	1.284873	2.30E-09	7.38E-08
343	SEMA3B	0.252939	6.720662	0.24999	1.284012	2.54E-09	8.12E-08
344	CRABP2	1.385018	1.706046	0.249444	1.283312	2.75E-09	8.76E-08
345	FHL1	0.135608	12.00953	0.248467	1.282058	3.17E-09	1.01E-07
346	NDRG1	1.050598	2.060071	0.248301	1.281846	3.25E-09	1.03E-07
347	SLC7A2	0.116518	13.85619	0.246669	1.279755	4.11E-09	1.30E-07
348	ZNF667-AS1	0.862764	2.376576	0.246425	1.279443	4.26E-09	1.34E-07
349	RP11-384O8.1	0.161237	10.16862	0.246004	1.278905	4.52E-09	1.42E-07
350	HIP1R	0.250703	6.748031	0.245948	1.278833	4.56E-09	1.43E-07
351	FGF2	2.018302	1.348742	0.244999	1.27762	5.23E-09	1.63E-07
352	KIT	0.030184	51.67617	0.24396	1.276293	6.06E-09	1.89E-07
353	C1QTNF1-AS1	0.10998	14.60129	0.243673	1.275927	6.31E-09	1.96E-07
354	KISS1	0.050088	31.29963	0.241629	1.273321	8.43E-09	2.61E-07
355	RP11-95M15.1	0.438258	4.087299	0.240342	1.271684	1.01E-08	3.12E-07
356	FAM110C	0.875345	2.334729	0.239556	1.270685	1.13E-08	3.47E-07
357	CAPN12	0.24435	6.863296	0.239415	1.270505	1.15E-08	3.53E-07
358	AKR1B10	0.133243	12.09926	0.239168	1.270192	1.19E-08	3.65E-07
359	PCDH17	0.13024	12.36324	0.239039	1.270028	1.21E-08	3.70E-07
360	LXN	0.208754	7.928182	0.238607	1.26948	1.29E-08	3.92E-07
361	TREM1	0.147034	10.99752	0.237228	1.26773	1.56E-08	4.73E-07
362	CYP3A5	0.042828	36.34043	0.237073	1.267534	1.59E-08	4.82E-07
363	SOX4	1.445921	1.638446	0.237045	1.267498	1.60E-08	4.83E-07
364	PAG1	0.130057	12.33936	0.235761	1.265872	1.91E-08	5.75E-07
365	PRDM1	0.821944	2.438345	0.235323	1.265317	2.03E-08	6.08E-07
366	EXOC3L4	0.045003	34.48427	0.233365	1.262842	2.65E-08	7.93E-07
367	DKK3	0.604114	3.099196	0.232146	1.261303	3.12E-08	9.32E-07
368	RP11-456O19.2	0.035672	43.29901	0.232099	1.261244	3.14E-08	9.36E-07
369	RP11-402P6.11	0.140747	11.39774	0.231528	1.260525	3.39E-08	1.01E-06
370	S1PR3	0.285436	5.911877	0.231483	1.260468	3.41E-08	1.01E-06
371	PLPP3	0.308894	5.506427	0.231439	1.260412	3.43E-08	1.01E-06
372	LINC00936	0.065044	23.93239	0.229004	1.257347	4.76E-08	1.40E-06
373	PTPRR	0.060492	25.68214	0.228708	1.256976	4.95E-08	1.45E-06
374	DNAJB4	0.298351	5.644892	0.225125	1.252479	7.94E-08	2.33E-06
375	HSPA1A	0.780794	2.50876	0.224375	1.25154	8.75E-08	2.56E-06
376	ALOX15B	0.174924	9.203326	0.222871	1.249659	1.06E-07	3.10E-06
377	TGFB2	0.341724	4.963851	0.217655	1.243158	2.07E-07	6.03E-06
378	NRG1	0.221369	7.348226	0.216904	1.242224	2.28E-07	6.61E-06
379	CEACAM6	0.106941	14.5925	0.216152	1.241291	2.50E-07	7.24E-06
380	PHKG1	0.423745	4.106761	0.216136	1.241271	2.51E-07	7.24E-06
381	ACKR3	1.335012	1.687814	0.214416	1.239138	3.11E-07	8.95E-06
382	TNC	0.464867	3.779681	0.212445	1.236698	3.97E-07	1.14E-05
383	IRS2	0.360933	4.702302	0.211801	1.235902	4.30E-07	1.23E-05
384	ZBED2	0.92081	2.185897	0.211486	1.235513	4.46E-07	1.27E-05

Supplementary Table S2. HVGs of lung airway epithelial cells (LAECs) (FDR<0.01).

385	RP11-538P18.2	0.217347	7.416996	0.209288	1.2328	5.84E-07	1.66E-05
386	ACTA2	0.430981	4.016452	0.208476	1.2318	6.44E-07	1.83E-05
387	GALNT5	0.284717	5.777392	0.206193	1.228991	8.47E-07	2.40E-05
388	UPP1	2.860597	1.080433	0.205892	1.22862	8.78E-07	2.48E-05
389	PTN	0.013831	107.6242	0.203325	1.225471	1.19E-06	3.35E-05
390	PSG5	0.06628	22.87868	0.202349	1.224276	1.33E-06	3.75E-05
391	CSF3	6.526953	0.78594	0.202114	1.223988	1.37E-06	3.84E-05
392	TMPRSS2	0.086917	17.56346	0.201441	1.223164	1.48E-06	4.15E-05
393	NKAIN4	0.039678	37.78772	0.2009	1.222502	1.58E-06	4.40E-05
394	KCNQ4	0.265915	6.113639	0.200885	1.222485	1.58E-06	4.40E-05
395	CDC20	0.148177	10.50203	0.198465	1.219529	2.09E-06	5.81E-05
396	FAM107B	0.548891	3.237891	0.197209	1.217999	2.42E-06	6.69E-05
397	CITED4	0.976057	2.063307	0.196659	1.217329	2.57E-06	7.10E-05
398	SELM	2.020536	1.283855	0.196321	1.216917	2.67E-06	7.36E-05
399	ALDH1A1	0.115046	13.33221	0.195942	1.216457	2.79E-06	7.67E-05
400	CTD-2008A1.3	0.048088	31.07767	0.194506	1.214711	3.28E-06	8.99E-05
401	BBC3	3.01046	1.042614	0.194462	1.214658	3.30E-06	9.01E-05
402	CCAT1	0.047565	31.36429	0.192954	1.212827	3.90E-06	0.000106
403	UNC5B-AS1	0.044147	33.74847	0.192923	1.212789	3.92E-06	0.000107
404	PSAT1	0.493906	3.521049	0.192862	1.212715	3.94E-06	0.000107
405	ALDH1A3	2.339425	1.179703	0.192131	1.211829	4.28E-06	0.000116
406	PGF	1.417993	1.584508	0.190918	1.210361	4.89E-06	0.000132
407	SGCE	0.057693	25.82901	0.18806	1.206906	6.69E-06	0.00018
408	HIST1H2BC	0.427892	3.956009	0.187126	1.20578	7.41E-06	0.000199
409	IL11	0.061827	24.11506	0.187078	1.205721	7.45E-06	0.0002
410	NAV3	0.243016	6.545191	0.186944	1.20556	7.55E-06	0.000202
411	BBOX1-AS1	0.022716	64.64566	0.186463	1.20498	7.96E-06	0.000212
412	PAQR7	0.573716	3.085834	0.185599	1.203939	8.73E-06	0.000232
413	LGALS7	0.385105	4.325182	0.185014	1.203235	9.30E-06	0.000247
414	NDRG2	0.127444	11.93478	0.183077	1.200907	1.14E-05	0.000302
415	C6orf58	0.044903	32.85737	0.182857	1.200642	1.17E-05	0.000309
416	LINC01133	0.43713	3.867352	0.182817	1.200595	1.17E-05	0.000309
417	CAPNS2	1.118822	1.841574	0.180802	1.198178	1.45E-05	0.000381
418	MGAM	0.059328	24.93516	0.180188	1.197442	1.55E-05	0.000406
419	NINJ1	7.43887	0.740216	0.178159	1.195015	1.91E-05	0.000499
420	MRGPRX4	0.03993	36.60537	0.17536	1.191676	2.54E-05	0.000663
421	DUSP2	0.181145	8.487587	0.174653	1.190833	2.73E-05	0.000711
422	TMEM47	0.08298	17.84214	0.172279	1.188009	3.46E-05	0.000899
423	S100A14	27.21845	0.595567	0.17215	1.187856	3.51E-05	0.000909
424	SLCO4A1	0.421254	3.948741	0.171814	1.187457	3.63E-05	0.000938
425	SDPR	0.188655	8.141208	0.170954	1.186437	3.95E-05	0.001019
426	ZNF19	0.029981	48.33648	0.170492	1.185889	4.13E-05	0.001064
427	SSPN	0.415413	3.988089	0.169674	1.184918	4.48E-05	0.00115

Supplementary Table S2. HVGs of lung airway epithelial cells (LAECs) (FDR<0.01).

428	GPX2	1.037469	1.921467	0.169634	1.184871	4.50E-05	0.001152
429	RRM2B	0.766095	2.405969	0.167835	1.182741	5.36E-05	0.00137
430	MFAP2	0.226233	6.844233	0.16589	1.180444	6.47E-05	0.001649
431	SOX7	1.74119	1.355254	0.163403	1.177511	8.20E-05	0.002085
432	ZFP57	0.147321	10.19509	0.163317	1.17741	8.27E-05	0.002096
433	MRAS	0.156751	9.613986	0.163299	1.177388	8.28E-05	0.002096
434	FA2H	0.104581	14.10617	0.160795	1.174444	0.000105	0.002645
435	TMPRSS4	0.51973	3.266204	0.1605	1.174098	0.000108	0.002712
436	LRRC8B	0.342132	4.682916	0.160451	1.17404	0.000108	0.002719
437	HEG1	0.394798	4.128502	0.160138	1.173673	0.000111	0.002792
438	IZUMO4	0.59429	2.922203	0.159974	1.173481	0.000113	0.002829
439	CSTA	16.60954	0.621542	0.159826	1.173307	0.000115	0.002861
440	GPAT3	0.067051	21.66174	0.158973	1.172306	0.000124	0.003089
441	KISS1R	0.092626	15.81792	0.158294	1.171511	0.000132	0.00328
442	KLK10	3.26289	0.969177	0.158269	1.171482	0.000132	0.00328
443	CDKN2D	0.515721	3.274923	0.15669	1.169632	0.000153	0.003781
444	INA	0.204828	7.428829	0.155941	1.168757	0.000164	0.004037
445	SRGAP3	0.143018	10.40815	0.1559	1.168709	0.000164	0.004043
446	IGFBP6	14.4719	0.631035	0.154903	1.167544	0.00018	0.004406
447	B3GNT7	0.177034	8.502417	0.154896	1.167537	0.00018	0.004406
448	GLIPR1	0.502554	3.340048	0.154694	1.167301	0.000183	0.004476
449	GPRC5A	3.332971	0.956042	0.154081	1.166585	0.000193	0.004719
450	PLAC8	0.566019	3.018372	0.152382	1.164605	0.000225	0.005476
451	CORO1A	0.401575	4.03517	0.152065	1.164236	0.000231	0.005619
452	ENC1	0.578869	2.960992	0.151643	1.163745	0.00024	0.005819
453	CXCL14	0.037151	38.38116	0.151611	1.163708	0.000241	0.005823
454	LIMD2	0.521447	3.225531	0.150724	1.162676	0.00026	0.00628
455	LYPD3	0.145256	10.19833	0.150256	1.162132	0.000271	0.006529
456	TGM1	0.341657	4.637465	0.149467	1.161216	0.00029	0.006977
457	RASD1	0.058231	24.62141	0.149272	1.160989	0.000295	0.007081
458	VAMP5	1.002364	1.92891	0.148659	1.160277	0.000311	0.007451
459	EDNRA	0.192267	7.821068	0.148528	1.160125	0.000315	0.007519
460	RGMB	0.731571	2.445817	0.148324	1.159889	0.00032	0.007636
461	ALOX5AP	0.034922	40.65659	0.148158	1.159696	0.000325	0.007729
462	WNT7B	0.968194	1.97704	0.148035	1.159554	0.000328	0.007794
463	KANK3	0.028442	49.77033	0.147615	1.159066	0.000341	0.008064
464	HES1	3.062057	0.985963	0.146505	1.157781	0.000374	0.008847
465	STMN1	1.257285	1.640465	0.145703	1.156853	0.000401	0.009451
466	HILPDA	0.558703	3.030023	0.145507	1.156625	0.000408	0.00959

Supplementary Table S3. HVGs of dermal fibroblasts (DFs) (FDR<0.01).

ID	genes	u	cv2	residualcv2	fitratio	pval	fdr
1	LCE1F	0.11622394	257.1515656	3.195180187	24.41457248	0	0
2	KCNMA1	2.710607015	11.76021911	2.432720858	11.38983007	0	0
3	PI16	1.332664816	14.23769248	2.268882944	9.668594413	0	0
4	ACAN	0.145091485	74.85891421	2.16875446	8.747382037	0	0
5	IGFBP5	0.169774619	58.38787051	2.065371893	7.888230927	0	0
6	COMP	1.333464644	10.98555331	2.009923829	7.46274888	0	0
7	KRTAP2-3	2.089282285	7.488008796	1.865672228	6.460277203	0	0
8	SFRP2	1.443987489	8.6424341	1.816052714	6.147544377	0	0
9	LCE3D	0.028694705	240.2860487	1.772904572	5.887930469	0	0
10	KRT19	0.766784531	12.21755918	1.755518775	5.786448827	0	0
11	KRT81	0.171641985	40.56672553	1.711249443	5.535873915	0	0
12	FLG	0.02066746	283.6378855	1.61478982	5.026831275	0	0
13	KRT14	0.062139907	94.9320355	1.599725874	4.951674857	0	0
14	G0S2	0.08644807	67.12607943	1.570984932	4.811384746	0	0
15	CXCL1	0.149379154	37.53743847	1.505517702	4.506486045	0	0
16	CPB1	0.018432075	272.2695607	1.460580035	4.308457861	0	0
17	MMP3	2.005975452	4.984338995	1.439079766	4.216813575	0	0
18	RGCC	0.308576538	17.40860048	1.387840332	4.006188666	0	0
19	CLU	1.385364019	5.494412163	1.339337132	3.816512822	2.470E-323	1.03E-320
20	KRT17	0.028399879	154.7717589	1.32285108	3.754109399	2.128E-313	8.43E-311
21	STMN2	0.183005172	25.74896686	1.315331216	3.725984885	0.00E+00	2.39E-306
22	PTX3	0.659102206	8.603949805	1.294743942	3.650061226	6.59E-297	2.37E-294
23	MGP	0.053060282	75.21416185	1.213580835	3.365514453	9.73E-253	3.35E-250
24	TFPI2	0.476864436	10.1519237	1.210391444	3.354797609	4.20E-251	1.39E-248
25	MMP1	0.622843624	8.239416434	1.209134578	3.350583729	1.84E-250	5.84E-248
26	CTSC	0.622497506	8.19315101	1.203084922	3.330375039	2.19E-247	6.68E-245
27	PCP4	0.025014179	152.2046013	1.180939767	3.257433994	2.36E-236	6.93E-234
28	MT1X	0.731227567	7.015367912	1.166685808	3.21133201	1.96E-229	5.53E-227
29	ACTC1	2.798823965	3.214096718	1.148621656	3.153842833	7.16E-221	1.96E-218
30	GAL	0.410660847	10.41962177	1.115194016	3.050159901	1.31E-205	3.46E-203
31	ACTA2	0.764502926	6.434854792	1.112249563	3.041192058	2.66E-204	6.81E-202
32	HIST1H4C	0.717467951	6.261606512	1.039264576	2.827137105	1.19E-173	2.95E-171
33	LUM	1.046949605	4.773762192	1.027338929	2.793621907	5.85E-169	1.41E-166
34	PPP1R14A	0.238261486	14.66259438	0.98991847	2.691015065	8.58E-155	2.00E-152
35	RARRES2	0.277495765	12.38108508	0.955049549	2.598799347	2.50E-142	5.66E-140
36	PENK	0.161902316	19.10913395	0.904760877	2.471340897	1.49E-125	3.29E-123
37	DCN	8.536137133	1.808968066	0.891066	2.437726884	3.20E-121	6.86E-119
38	DKK1	6.575192121	1.901119743	0.887862389	2.429929851	3.20E-120	6.66E-118
39	SERPINE2	10.70646564	1.681350762	0.855523153	2.352604831	1.94E-110	3.94E-108
40	SCG5	0.405919234	8.075299376	0.850753251	2.341409858	4.84E-109	9.59E-107

Supplementary Table S3. HVGs of dermal fibroblasts (DFs) (FDR<0.01).

41	CEMIP	1.322792105	3.447067322	0.846136103	2.330624141	1.06E-107	2.05E-105
42	OLFM2	0.472448981	7.092601032	0.844336702	2.326434184	3.52E-107	6.64E-105
43	GRB14	0.016233113	161.8995943	0.814871002	2.258884263	6.77E-99	1.25E-96
44	SFRP4	0.02730878	95.71149818	0.803627141	2.233627937	7.57E-96	1.36E-93
45	KRTAP1-5	0.417855246	7.471794096	0.796898079	2.218648172	4.72E-94	8.32E-92
46	IGFBP7	4.470026615	1.910636948	0.792457203	2.208817275	7.03E-93	1.21E-90
47	IL1RL1	0.120287755	22.49071424	0.790972246	2.20553971	1.73E-92	2.91E-90
48	PTTG1	0.658360469	5.199809153	0.790308279	2.204075791	2.58E-92	4.25E-90
49	NMB	0.469429269	6.723609093	0.785770754	2.194097397	3.93E-91	6.36E-89
50	KRT34	0.162173513	16.88959977	0.782834661	2.187664773	2.27E-90	3.60E-88
51	TNFRSF11B	0.825218271	4.264411315	0.754721724	2.127019542	2.73E-83	4.24E-81
52	POSTN	0.482966559	6.367864914	0.754144423	2.125791967	3.78E-83	5.76E-81
53	PGF	0.727553143	4.613866805	0.744008313	2.10435354	1.09E-80	1.63E-78
54	KRT16	0.012994266	185.9260266	0.732389522	2.080044987	6.30E-78	9.25E-76
55	AKR1C1	0.552787794	5.548831567	0.722624149	2.059831429	1.19E-75	1.71E-73
56	STC1	0.063695679	37.83992291	0.703865414	2.021551758	2.11E-71	2.99E-69
57	PSG5	2.238111629	2.23204384	0.687477379	1.988692483	8.17E-68	1.14E-65
58	MT1E	1.584870766	2.610027187	0.670565572	1.955342896	3.11E-64	4.25E-62
59	ATF5	0.533666087	5.37423043	0.663272376	1.941134073	9.99E-63	1.34E-60
60	LHX9	0.011148444	201.6538261	0.661349865	1.937405806	2.47E-62	3.26E-60
61	ANKRD1	0.038921668	58.56297915	0.660729251	1.936203799	3.31E-62	4.30E-60
62	SMYD3	0.480627874	5.821846958	0.660625243	1.936002429	3.47E-62	4.44E-60
63	KRT7	2.345035438	2.121825765	0.657998569	1.930923853	1.19E-61	1.50E-59
64	CRIP1	1.560540255	2.597098407	0.657133708	1.929254594	1.78E-61	2.20E-59
65	ADH1B	0.013215627	168.2803069	0.649447892	1.914483536	6.23E-60	7.59E-58
66	ADIRF	7.262058066	1.461762773	0.646500972	1.908850011	2.40E-59	2.88E-57
67	FST	0.635122366	4.59495802	0.639833088	1.89616436	4.91E-58	5.81E-56
68	CDKN3	0.401934486	6.588717289	0.639165877	1.894899641	6.63E-58	7.73E-56
69	STMN1	1.019165102	3.281631167	0.635118334	1.887245454	4.05E-57	4.65E-55
70	HSPB7	2.073515128	2.169616807	0.623304043	1.865080178	7.31E-55	8.27E-53
71	SPON2	0.375154584	6.790549753	0.6120862	1.844274914	9.00E-53	1.00E-50
72	NEFM	0.044094929	49.36047491	0.611906657	1.843943817	9.71E-53	1.07E-50
73	DPP4	0.085879601	25.70052041	0.604612088	1.830541981	2.08E-51	2.26E-49
74	UCHL1	0.923323912	3.397685824	0.60448746	1.83031386	2.20E-51	2.35E-49
75	IGF1	0.057211102	38.00022516	0.604030476	1.829477626	2.66E-51	2.81E-49
76	CRLF1	0.494860838	5.360063284	0.601207735	1.824320766	8.57E-51	8.94E-49
77	S100A2	0.30732414	7.875652405	0.591152134	1.806068049	5.26E-49	5.41E-47
78	SERPINB2	0.111187578	19.678769	0.583254799	1.791861097	1.25E-47	1.27E-45
79	BAMBI	0.183084982	12.31089383	0.577818968	1.782147268	1.07E-46	1.08E-44
80	CYP1B1	0.748384797	3.820023235	0.575534097	1.77807994	2.63E-46	2.60E-44
81	ADAMTS1	1.10262529	2.915593158	0.567378951	1.763638405	6.19E-45	6.05E-43
82	IGFBP2	0.240869619	9.485022238	0.564001226	1.757691369	2.25E-44	2.17E-42
83	TGM2	0.205168072	10.86852744	0.556543125	1.744631092	3.76E-43	3.59E-41

Supplementary Table S3. HVGs of dermal fibroblasts (DFs) (FDR<0.01).

84	NQO1	2.975916898	1.711582141	0.54285619	1.720915109	5.81E-41	5.49E-39
85	HIST1H1C	0.440066764	5.549013409	0.541640347	1.718824019	9.03E-41	8.42E-39
86	TNNT1	0.10077774	20.69405889	0.54044742	1.71677481	1.39E-40	1.28E-38
87	LSP1	0.098652524	21.07003547	0.538201366	1.712923167	3.11E-40	2.84E-38
88	TUBA1B	10.13944349	1.229797742	0.534379495	1.706389091	1.22E-39	1.10E-37
89	CYTL1	0.033590266	59.48878519	0.531851928	1.702081523	2.98E-39	2.65E-37
90	PRR15	0.029453971	67.03211603	0.521969031	1.685342877	9.37E-38	8.25E-36
91	CSRP2	0.361682618	6.396842068	0.521724645	1.684931054	1.02E-37	8.88E-36
92	HOXA4	0.039849071	49.53613731	0.516398924	1.675981433	6.31E-37	5.44E-35
93	OSR2	0.363403733	6.329385848	0.515110311	1.673823133	9.78E-37	8.33E-35
94	CCL26	0.367662419	6.228650306	0.508838362	1.663357853	8.07E-36	6.80E-34
95	WNT2	0.104547155	19.19885614	0.500290683	1.649200596	1.36E-34	1.13E-32
96	PSG4	0.618811679	4.070441325	0.499060976	1.647173808	2.03E-34	1.67E-32
97	MT2A	16.61559754	1.108486458	0.49403043	1.638908432	1.03E-33	8.44E-32
98	CFD	0.258608608	8.300132187	0.493369802	1.63782608	1.28E-33	1.03E-31
99	AJAP1	0.014125225	133.7535074	0.485902492	1.625641475	1.37E-32	1.10E-30
100	CLIC3	1.349274391	2.36303721	0.48020053	1.616398506	8.18E-32	6.48E-30
101	COL15A1	0.078980881	24.42838171	0.4735846	1.605739824	6.27E-31	4.92E-29
102	MFGE8	3.492876964	1.500425321	0.470603991	1.600960867	1.55E-30	1.21E-28
103	NDUFA4L2	0.112924347	17.19500578	0.462970008	1.588785691	1.53E-29	1.18E-27
104	UBE2C	0.187698984	10.69548749	0.459833703	1.583810579	3.86E-29	2.94E-27
105	JAG1	0.033597768	55.30934906	0.459225512	1.582847614	4.62E-29	3.49E-27
106	DPT	0.824578047	3.141737393	0.448652301	1.566199996	9.88E-28	7.39E-26
107	LY6K	0.198159568	10.05792016	0.447608963	1.564566772	1.33E-27	9.85E-26
108	CSTA	0.048713136	37.96394784	0.446625785	1.56302928	1.76E-27	1.29E-25
109	TAGLN	12.40049198	1.093100988	0.445755468	1.561669541	2.25E-27	1.64E-25
110	ARRDC2	0.054889959	33.7291751	0.444570229	1.559819686	3.15E-27	2.26E-25
111	APOE	0.020457049	88.90675899	0.444556146	1.55979772	3.16E-27	2.26E-25
112	HAPLN1	0.054832557	33.62758764	0.440536884	1.553541066	9.77E-27	6.92E-25
113	PTGDS	0.11904486	15.9924915	0.440217783	1.553045409	1.07E-26	7.49E-25
114	MEST	0.03227116	56.43257157	0.439730419	1.552288694	1.22E-26	8.51E-25
115	IFI27	0.153254137	12.62644487	0.439693826	1.552231893	1.24E-26	8.52E-25
116	SULF1	1.854047938	1.904169517	0.437730929	1.54918801	2.13E-26	1.46E-24
117	THBD	0.144316325	13.2980345	0.435787589	1.546180334	3.65E-26	2.47E-24
118	FBXO32	0.182174872	10.6788255	0.431050755	1.538873654	1.34E-25	8.97E-24
119	CDKN1A	2.486179164	1.635099304	0.42316904	1.526792364	1.11E-24	7.41E-23
120	EFEMP1	0.355979471	5.837033501	0.416772884	1.517057926	6.00E-24	3.96E-22
121	ADGRG1	0.018716381	94.38888205	0.416369361	1.516445882	6.67E-24	4.37E-22
122	IL12A	0.025974804	68.16579798	0.414842497	1.514132241	9.92E-24	6.45E-22
123	CRABP2	2.265305368	1.68907489	0.414268892	1.513263977	1.15E-23	7.42E-22
124	PSMC3IP	0.074582107	24.25690937	0.411459647	1.509018813	2.38E-23	1.52E-21
125	CLDN11	0.616205537	3.727946477	0.407981568	1.503779444	5.79E-23	3.67E-21
126	ZNF124	0.014165043	122.8583359	0.403729976	1.497399559	1.70E-22	1.07E-20

Supplementary Table S3. HVGs of dermal fibroblasts (DFs) (FDR<0.01).

127	ITPRIP	0.073491961	24.41221733	0.403669305	1.497308712	1.72E-22	1.08E-20
128	CXCL3	0.023914011	72.96919132	0.401344559	1.493831893	3.09E-22	1.91E-20
129	GLRX	1.774388617	1.8772043	0.400996811	1.493312506	3.37E-22	2.07E-20
130	CEBPD	0.457991627	4.66538292	0.400486194	1.49255019	3.82E-22	2.33E-20
131	SOCS2	0.617832703	3.689977571	0.399735276	1.491429828	4.61E-22	2.79E-20
132	BIRC5	0.303887754	6.541910565	0.395918245	1.485747846	1.18E-21	7.05E-20
133	NTF3	0.105145245	17.078456	0.388664029	1.475008908	6.90E-21	4.08E-19
134	EDN1	0.090418325	19.57256716	0.381448395	1.464404089	3.83E-20	2.25E-18
135	PRSS3	0.115032595	15.53674289	0.379009762	1.460837296	6.78E-20	3.95E-18
136	FGF7	3.691962268	1.34082321	0.377325153	1.458378428	1.00E-19	5.81E-18
137	CDC20	0.140219369	12.87009698	0.376283784	1.456860509	1.28E-19	7.34E-18
138	FABP5	0.168516572	10.85056021	0.375650526	1.455938233	1.48E-19	8.43E-18
139	SOX4	0.405714736	5.020968431	0.375150881	1.455210961	1.66E-19	9.39E-18
140	RNLS	0.034951215	48.89055349	0.374661893	1.454499555	1.86E-19	1.04E-17
141	PSG2	0.02020677	83.4908371	0.369525368	1.447047637	6.00E-19	3.35E-17
142	PTPRN2	0.014997826	112.1110554	0.368881328	1.44611598	6.94E-19	3.85E-17
143	CD9	0.129079518	13.79288757	0.36822389	1.445165562	8.05E-19	4.43E-17
144	F2R	0.185104183	9.796868399	0.359397078	1.432465489	5.73E-18	3.13E-16
145	QPRT	0.133140016	13.24544744	0.356698998	1.428605792	1.03E-17	5.61E-16
146	HSPB6	1.595702818	1.898409161	0.35592977	1.427507291	1.22E-17	6.58E-16
147	PARD3	0.109631032	15.8618686	0.354308252	1.425194438	1.73E-17	9.28E-16
148	RSPO3	0.068329609	24.90950414	0.353620453	1.424214528	2.01E-17	1.07E-15
149	HMOX1	0.483981839	4.258819863	0.353546417	1.424109088	2.04E-17	1.08E-15
150	SOD3	0.688933888	3.240008457	0.350778886	1.420173271	3.70E-17	1.94E-15
151	ELN	7.227647442	1.088493017	0.350664938	1.420011454	3.79E-17	1.97E-15
152	CXCL6	0.065535144	25.81263998	0.348898867	1.417505826	5.51E-17	2.85E-15
153	OLFML2B	0.103849539	16.57847121	0.347197773	1.415096565	7.89E-17	4.06E-15
154	MELK	0.069647673	24.24816187	0.345148788	1.412200022	1.21E-16	6.20E-15
155	SFRP1	0.073721145	22.85226913	0.340633567	1.405838001	3.10E-16	1.57E-14
156	DIO2	0.020774291	78.76754897	0.338692677	1.40311207	4.62E-16	2.33E-14
157	CH25H	0.234587879	7.748126239	0.338250288	1.402491486	5.05E-16	2.54E-14
158	CLSPN	0.029854139	55.04411475	0.338219587	1.402448429	5.09E-16	2.54E-14
159	THBS1	2.094028266	1.619964273	0.335853663	1.399134265	8.23E-16	4.08E-14
160	SCUBE3	0.055452678	29.93490535	0.335144115	1.398141864	9.51E-16	4.68E-14
161	CXCL8	0.024267492	67.1870836	0.33327778	1.395534897	1.39E-15	6.75E-14
162	ABCB9	0.03072078	53.25093687	0.333267061	1.395519937	1.39E-15	6.75E-14
163	CENPF	0.165418716	10.54680083	0.330200427	1.391246944	2.56E-15	1.24E-13
164	OXTR	0.429100102	4.58233543	0.329688757	1.390535267	2.84E-15	1.36E-13
165	SERPINE1	8.909273736	1.022112534	0.327837718	1.387963713	4.09E-15	1.95E-13
166	TSTD1	0.121484465	14.01316681	0.327174702	1.387043776	4.66E-15	2.21E-13
167	CDA	0.817749611	2.798419052	0.327124066	1.386973543	4.71E-15	2.22E-13
168	TINAGL1	0.061301498	26.90830029	0.325842564	1.385197272	6.06E-15	2.84E-13
169	PTN	0.190018603	9.245674226	0.32533994	1.384501213	6.68E-15	3.12E-13

Supplementary Table S3. HVGs of dermal fibroblasts (DFs) (FDR<0.01).

170	SLC35E3	0.083911494	19.8522181	0.324224027	1.382957092	8.31E-15	3.85E-13
171	ITGA2	0.130915095	13.0015075	0.322352591	1.380371397	1.19E-14	5.51E-13
172	FABP3	0.03277007	49.41422912	0.322005935	1.379892966	1.28E-14	5.85E-13
173	GADD45B	1.451515666	1.926042797	0.317787674	1.374084476	2.87E-14	1.30E-12
174	PRAC2	0.014171007	112.4397922	0.315533824	1.370990983	4.40E-14	1.98E-12
175	SNCG	0.169367111	10.16076545	0.314591161	1.369699211	5.26E-14	2.35E-12
176	IL32	0.099013054	16.74945294	0.312182631	1.366404217	8.26E-14	3.68E-12
177	IGFBP3	28.51558265	0.883686252	0.311164692	1.36501401	9.98E-14	4.42E-12
178	PEG10	0.041429646	38.79169485	0.309986118	1.363406187	1.24E-13	5.47E-12
179	AKAP12	0.29394755	6.169296568	0.308535735	1.361430159	1.62E-13	7.11E-12
180	TLCD2	0.036029853	44.39536168	0.308050246	1.360769361	1.78E-13	7.73E-12
181	FOXL1	0.03510938	45.52315351	0.307732174	1.360336606	1.88E-13	8.15E-12
182	UBE2S	0.479143241	4.097679221	0.30695836	1.359284366	2.17E-13	9.34E-12
183	HAPLN3	0.269736394	6.616396393	0.30363858	1.354779324	3.97E-13	1.70E-11
184	GLIPR1	1.48971748	1.870787798	0.303326565	1.354356678	4.20E-13	1.79E-11
185	ADRA2A	0.139272843	12.01415456	0.30115339	1.351416619	6.21E-13	2.63E-11
186	COL8A1	1.043164307	2.312169555	0.30005338	1.349930865	7.56E-13	3.19E-11
187	H2AFZ	5.080781173	1.125724167	0.29997078	1.349819365	7.67E-13	3.22E-11
188	APOC1	0.025071802	62.56812627	0.294241122	1.342107477	2.11E-12	8.80E-11
189	AP5B1	0.085960684	18.80933988	0.293357284	1.340921795	2.46E-12	1.02E-10
190	FBN2	0.145242791	11.45646736	0.292672	1.340003197	2.77E-12	1.14E-10
191	CYP26B1	0.031739858	49.483619	0.291999804	1.339102755	3.12E-12	1.28E-10
192	MOK	0.135502087	12.16978042	0.288425989	1.334325591	5.76E-12	2.35E-10
193	ANKRD50	0.064287642	24.71512943	0.286865683	1.332245258	7.52E-12	3.05E-10
194	TM4SF1	0.038382798	40.82326148	0.286214688	1.331378255	8.39E-12	3.39E-10
195	ACP5	0.019565084	79.16361884	0.284368235	1.328922197	1.15E-11	4.61E-10
196	PCOLCE	1.65392343	1.726881177	0.280552289	1.323860764	2.17E-11	8.67E-10
197	FGD6	0.011325748	135.5594504	0.27989376	1.32298925	2.42E-11	9.62E-10
198	TSPAN13	0.203169474	8.31159056	0.279483358	1.322446404	2.59E-11	1.02E-09
199	SUGCT	0.223126028	7.636663094	0.27904865	1.321871651	2.78E-11	1.10E-09
200	F3	0.423599477	4.400846438	0.278738745	1.32146206	2.92E-11	1.15E-09
201	SMC6	0.146483293	11.20680295	0.278537091	1.321195609	3.02E-11	1.18E-09
202	DKK2	0.072207688	21.89806436	0.278005062	1.320492882	3.30E-11	1.28E-09
203	MARCKSL1	0.403745773	4.571636102	0.277388351	1.319678771	3.65E-11	1.41E-09
204	DUSP2	0.020755472	74.14233942	0.277281815	1.319538184	3.71E-11	1.43E-09
205	LNPEP	0.087783657	18.08044178	0.27390267	1.315086799	6.43E-11	2.46E-09
206	HSPA5	1.484076032	1.817724749	0.272423711	1.313143277	8.16E-11	3.11E-09
207	HAS2	0.401293782	4.551979648	0.268052377	1.307415617	1.63E-10	6.20E-09
208	HES1	0.189347505	8.746556689	0.266627286	1.305553758	2.04E-10	7.72E-09
209	LMCD1	0.356363711	5.017831962	0.266456694	1.30533106	2.10E-10	7.89E-09
210	SPEN	0.131445399	12.2476135	0.266399959	1.305257004	2.12E-10	7.92E-09
211	TACSTD2	0.025985733	58.69119103	0.265604213	1.304218764	2.40E-10	8.93E-09
212	DIRAS3	0.052260382	29.53453446	0.264020935	1.302155457	3.07E-10	1.14E-08

Supplementary Table S3. HVGs of dermal fibroblasts (DFs) (FDR<0.01).

213	NREP	1.646107481	1.696230686	0.260108143	1.297070349	5.60E-10	2.07E-08
214	PRSS23	4.534675476	1.11570063	0.258765683	1.295330251	6.87E-10	2.52E-08
215	CKB	0.497295992	3.790121077	0.258522744	1.295015603	7.13E-10	2.60E-08
216	DDX10	0.081904394	19.02104356	0.25825607	1.294670303	7.43E-10	2.70E-08
217	MEDAG	0.158478357	10.2077192	0.258024887	1.294371032	7.69E-10	2.78E-08
218	ASH2L	0.087908285	17.75341149	0.257005618	1.29305239	8.97E-10	3.23E-08
219	BMF	0.028431746	53.2174937	0.256391683	1.292258785	9.84E-10	3.53E-08
220	CCL2	0.066539194	23.1833027	0.256160991	1.291960705	1.02E-09	3.63E-08
221	BCL2L12	0.194782542	8.434370814	0.255985087	1.291733465	1.05E-09	3.72E-08
222	CXCL14	0.030339532	49.58742819	0.249698658	1.283638544	2.65E-09	9.38E-08
223	ISG20	0.041612923	36.29904658	0.247891127	1.281320423	3.45E-09	1.21E-07
224	CLEC2B	0.313376227	5.493591649	0.24772267	1.281104595	3.53E-09	1.24E-07
225	STC2	1.731878476	1.629708012	0.246999184	1.280178069	3.92E-09	1.37E-07
226	CD70	0.027096986	55.21040263	0.245764184	1.278598024	4.68E-09	1.63E-07
227	CENPM	0.173555527	9.264518618	0.244657023	1.277183193	5.49E-09	1.90E-07
228	LYPD3	0.012051306	122.7351416	0.242227074	1.274083472	7.75E-09	2.67E-07
229	MLLT11	0.217742493	7.511447094	0.240630421	1.272050825	9.70E-09	3.33E-07
230	ZNF232	0.031530738	47.30896563	0.240555784	1.271955887	9.81E-09	3.35E-07
231	APBB1IP	0.185495077	8.670037336	0.239129533	1.270143051	1.20E-08	4.07E-07
232	ID4	0.257786317	6.446371372	0.237811895	1.268470564	1.44E-08	4.87E-07
233	TRIB3	0.449487861	4.024217765	0.237516507	1.268095929	1.50E-08	5.05E-07
234	C11orf96	0.075896401	20.00946705	0.23576717	1.265879542	1.91E-08	6.40E-07
235	PTGER1	0.081132684	18.73808675	0.234190877	1.263885716	2.36E-08	7.88E-07
236	INHBA	0.49794337	3.695180398	0.23418518	1.263878515	2.37E-08	7.88E-07
237	FAM43A	0.148655669	10.57435335	0.234108402	1.263781481	2.39E-08	7.93E-07
238	TNNC1	0.013207848	111.0189044	0.232932601	1.262296399	2.81E-08	9.27E-07
239	PALM	0.126713098	12.25597891	0.232748048	1.26206346	2.88E-08	9.46E-07
240	RASSF2	0.013067793	111.9916993	0.231069458	1.25994675	3.61E-08	1.18E-06
241	S1PR3	0.057154622	26.18896307	0.230817592	1.259629453	3.73E-08	1.22E-06
242	NFATC2	0.013347371	109.6315276	0.230792429	1.259597756	3.75E-08	1.22E-06
243	TACC3	0.053867568	27.73477726	0.230615245	1.259374596	3.84E-08	1.24E-06
244	MFAP4	0.691311004	2.863999595	0.229949043	1.258535878	4.19E-08	1.35E-06
245	SERPINF1	0.315328771	5.352253022	0.226987881	1.25481466	6.21E-08	1.99E-06
246	FMN1	0.074682843	20.1322357	0.226379215	1.25405113	6.73E-08	2.15E-06
247	TROAP	0.089270004	16.96159264	0.226066421	1.253658932	7.02E-08	2.23E-06
248	TPX2	0.124926654	12.328757	0.225351506	1.252762993	7.70E-08	2.44E-06
249	KLHL24	0.072160306	20.76258085	0.224126785	1.251229647	9.04E-08	2.85E-06
250	MDFI	0.017366235	83.79994883	0.223210787	1.250084047	1.02E-07	3.20E-06
251	CADM1	0.041970266	35.10254873	0.222740066	1.249495745	1.08E-07	3.39E-06
252	CCDC152	0.022294408	65.40760292	0.222658973	1.249394424	1.09E-07	3.41E-06
253	FOLR3	0.071019551	21.03422973	0.22176899	1.248282979	1.23E-07	3.81E-06
254	DNAJC18	0.09327751	16.18489579	0.221095053	1.247441998	1.34E-07	4.14E-06
255	PRC1	0.097386429	15.51240307	0.219705847	1.245710248	1.60E-07	4.93E-06

Supplementary Table S3. HVGs of dermal fibroblasts (DFs) (FDR<0.01).

256	SYNPO2	0.282964671	5.829257492	0.2187781	1.244555079	1.80E-07	5.52E-06
257	CCDC8	0.079526316	18.78657713	0.217588292	1.243075179	2.09E-07	6.40E-06
258	IER3	0.656830485	2.937427543	0.217491971	1.24295545	2.12E-07	6.45E-06
259	C15orf48	0.011706787	123.0175543	0.215701298	1.240731714	2.65E-07	8.03E-06
260	NPHP4	0.036557074	39.90539886	0.215681541	1.240707201	2.66E-07	8.03E-06
261	PHACTR3	0.172064959	9.051694429	0.21351042	1.238016398	3.48E-07	1.05E-05
262	HMGCS1	0.06395427	23.04620773	0.211921881	1.236051322	4.23E-07	1.27E-05
263	NSG1	0.011743967	122.1535355	0.211804455	1.235906186	4.29E-07	1.28E-05
264	BAALC	1.149545545	1.989411415	0.211315454	1.235301974	4.56E-07	1.36E-05
265	PLXNA2	0.07954308	18.65816669	0.210931905	1.234828267	4.78E-07	1.42E-05
266	SRGN	0.056385716	26.00677597	0.210685042	1.23452347	4.92E-07	1.46E-05
267	CARD9	0.078816404	18.81542282	0.210514271	1.234312669	5.03E-07	1.48E-05
268	DDIT4	0.552915277	3.314827548	0.207621007	1.230746636	7.14E-07	2.09E-05
269	ACKR4	0.030581923	47.14009294	0.206917196	1.229880729	7.77E-07	2.27E-05
270	PAPPA	0.447888789	3.91246973	0.206471513	1.229332714	8.19E-07	2.39E-05
271	GTSE1	0.046361089	31.31503429	0.205806135	1.228515015	8.87E-07	2.57E-05
272	CHRNE	0.025111775	57.17506192	0.205675281	1.228354269	9.01E-07	2.61E-05
273	KRT8	0.012969203	110.0025082	0.205626263	1.22829406	9.06E-07	2.61E-05
274	CHN1	0.291270692	5.606029773	0.204865813	1.227360358	9.92E-07	2.85E-05
275	VTA1	0.133812303	11.32232535	0.204527844	1.226945618	1.03E-06	2.95E-05
276	TMEM233	0.021969195	65.15712111	0.204296574	1.226661896	1.06E-06	3.02E-05
277	PDE1A	0.045656177	31.71028621	0.203389438	1.225549651	1.18E-06	3.35E-05
278	TUBB4B	1.054216628	2.083041949	0.202488386	1.224445864	1.31E-06	3.71E-05
279	YLPM1	0.134378388	11.25271291	0.202303944	1.224220046	1.34E-06	3.78E-05
280	ANGPT1	0.043695444	33.04394144	0.201699368	1.223480136	1.44E-06	4.04E-05
281	TUBA1A	2.198893396	1.38431163	0.201605235	1.223364972	1.46E-06	4.07E-05
282	IL7R	0.152825259	9.974873398	0.201376106	1.223084695	1.49E-06	4.17E-05
283	SYNGR2	0.049873962	29.02356588	0.201051975	1.222688319	1.55E-06	4.32E-05
284	LBH	0.859254652	2.383239138	0.200886837	1.222486424	1.58E-06	4.38E-05
285	MSX1	0.380201522	4.447079835	0.199950126	1.221341843	1.76E-06	4.87E-05
286	KCTD5	0.109512236	13.60341401	0.199685918	1.221019198	1.82E-06	5.00E-05
287	SCARA3	0.436497229	3.964406694	0.198758062	1.219886793	2.02E-06	5.55E-05
288	NGFR	0.040999591	35.05988692	0.198624433	1.219723791	2.05E-06	5.62E-05
289	RIPK4	0.014361838	98.68651244	0.198340948	1.219378067	2.12E-06	5.78E-05
290	IL33	0.01290407	109.4117776	0.195240935	1.215603833	3.02E-06	8.20E-05
291	TK1	0.445749207	3.880580784	0.194410444	1.214594704	3.32E-06	8.97E-05
292	CYGB	0.263815263	6.040335014	0.193082875	1.212983315	3.85E-06	0.000103747
293	CYYR1	0.01539492	91.61694224	0.192932617	1.212801068	3.91E-06	0.000105144
294	SBSN	0.311883665	5.219805791	0.192500724	1.212277381	4.11E-06	0.000109954
295	MAFB	0.099833845	14.71393232	0.190457048	1.209802409	5.15E-06	0.000137402
296	PTGER3	0.020606366	68.43541039	0.190053193	1.209313923	5.38E-06	0.00014316
297	CXCL2	0.023234296	60.74358111	0.189486843	1.208629221	5.73E-06	0.000151826
298	WNT16	0.055352563	25.8994856	0.188586153	1.207541112	6.32E-06	0.00016697

Supplementary Table S3. HVGs of dermal fibroblasts (DFs) (FDR<0.01).

299	PAN3	0.04238924	33.57316549	0.18791094	1.206726039	6.80E-06	0.000179102
300	TRIP13	0.061989209	23.18804811	0.187850333	1.206652905	6.85E-06	0.000179687
301	TNFAIP6	0.084149421	17.27285442	0.187756009	1.206539094	6.92E-06	0.000180938
302	C11orf87	0.016502345	85.02835315	0.187188315	1.205854344	7.36E-06	0.000191784
303	TOP2A	0.093663657	15.57931746	0.186898416	1.205504819	7.59E-06	0.000197241
304	SLC20A1	0.17756043	8.550126439	0.185295741	1.203574333	9.02E-06	0.000233583
305	WNT5B	0.733158978	2.623953122	0.185168942	1.203421731	9.14E-06	0.000236004
306	CD163L1	0.015319722	91.32340984	0.184866251	1.203057522	9.44E-06	0.000242976
307	TNFRSF19	0.082046317	17.64143841	0.18462045	1.202761845	9.69E-06	0.00024863
308	DCUN1D1	0.108266795	13.53138378	0.183558796	1.201485606	1.09E-05	0.000277464
309	CCNB1	0.099293247	14.68250186	0.183159227	1.201005626	1.13E-05	0.000288535
310	TSPAN2	0.152215598	9.806270861	0.180628576	1.197970142	1.48E-05	0.000375271
311	NAT10	0.064374487	22.18658788	0.180243826	1.197509312	1.54E-05	0.000389387
312	CST6	0.095561053	15.17851192	0.179939116	1.197144474	1.59E-05	0.000400655
313	MKI67	0.115241743	12.70582231	0.179575131	1.196708811	1.65E-05	0.000414776
314	SMIM3	0.431650312	3.921690811	0.178834443	1.195822751	1.78E-05	0.000446433
315	SLC7A11	0.37105965	4.441123399	0.178287204	1.19516853	1.88E-05	0.000470884
316	FECH	0.106148586	13.69941803	0.177196708	1.193865913	2.11E-05	0.000525054
317	BDKRB1	0.099025557	14.61648052	0.176086745	1.192541502	2.36E-05	0.000586223
318	CNN1	0.184611899	8.167998092	0.175131142	1.191402449	2.60E-05	0.000642396
319	PTHLH	0.021746746	63.923706	0.175123822	1.191393728	2.60E-05	0.000642396
320	MARCH4	0.229771208	6.702821376	0.174839833	1.191055433	2.68E-05	0.000659065
321	LYPD6B	0.116530975	12.51222947	0.174707101	1.190897353	2.71E-05	0.000665896
322	LMOD1	0.040578147	34.53418484	0.173401122	1.189343081	3.10E-05	0.000757078
323	SYT1	0.026920686	51.64137731	0.172499976	1.188271792	3.39E-05	0.000825924
324	GXYLT1	0.047761233	29.40948397	0.172059991	1.187749086	3.54E-05	0.000860284
325	NRG1	0.392909775	4.207784755	0.171961338	1.187631916	3.57E-05	0.00086611
326	LURAP1	0.06848831	20.72392659	0.171899229	1.187558156	3.60E-05	0.000868818
327	SLC4A7	0.09412737	15.259883	0.170887688	1.186357499	3.98E-05	0.000957525
328	IL6	0.016567355	83.29648005	0.17050765	1.185906725	4.13E-05	0.000991119
329	GALE	0.089054176	16.08010328	0.170385408	1.185761765	4.18E-05	0.001000105
330	PHF7	0.038953125	35.80736009	0.16957165	1.184797235	4.53E-05	0.001080142
331	ANG	0.104960323	13.71318388	0.16753585	1.182387678	5.52E-05	0.001313363
332	ALDH1A3	0.295098172	5.339018349	0.167375316	1.18219788	5.61E-05	0.001329956
333	LAPTM5	0.052374006	26.7526322	0.167207224	1.181999179	5.70E-05	0.001347737
334	FAH	0.281768355	5.55323444	0.166580239	1.181258314	6.05E-05	0.001427599
335	FGF9	0.055088853	25.41140076	0.164920218	1.179299028	7.10E-05	0.001669057
336	WDYHV1	0.161161971	9.145516999	0.163633968	1.17778313	8.02E-05	0.001880639
337	COL3A1	8.066576234	0.88304751	0.163387754	1.177493179	8.21E-05	0.001919309
338	WFDC1	0.015015876	91.14783033	0.163067387	1.177116009	8.46E-05	0.001972489
339	NPW	0.133394711	10.89161623	0.162824376	1.176829992	8.66E-05	0.002012316
340	ADM	3.420797448	1.110667214	0.162418201	1.17635209	9.00E-05	0.00208466
341	PIP5KL1	0.138973328	10.4742569	0.161982361	1.175839501	9.37E-05	0.002165441

Supplementary Table S3. HVGs of dermal fibroblasts (DFs) (FDR<0.01).

342	RASGRP2	0.034555599	39.96585822	0.161924006	1.175770886	9.42E-05	0.002170995
343	HEXIM2	0.068622644	20.43899049	0.15994598	1.173447479	0.000113318	0.002603027
344	FLNC	1.266956234	1.780526176	0.15986691	1.173354698	0.000114151	0.002614587
345	RCAN1	0.269148124	5.719308284	0.156023358	1.168853505	0.000162293	0.003706551
346	TBC1D1	0.123779248	11.59631391	0.155443805	1.168176288	0.000171015	0.003894517
347	TNXB	0.157796417	9.248031777	0.155310304	1.168020346	0.000173085	0.003926657
348	PINLYP	0.082589632	17.02346885	0.155289015	1.16799548	0.000173417	0.003926657
349	PPFIA4	0.017581163	77.27701587	0.154363167	1.166914594	0.000188454	0.004254989
350	LHB	0.01753279	77.48179196	0.154279547	1.166817022	0.000189871	0.004274787
351	PRELP	0.090606724	15.56359437	0.154240785	1.166771794	0.000190531	0.004277492
352	CEP55	0.053275548	25.95466367	0.153531243	1.165944214	0.000202993	0.004544408
353	APOD	0.137441197	10.47258314	0.151488122	1.163564481	0.000243243	0.005430138
354	TMEM200A	0.088259236	15.90993514	0.151179524	1.163205462	0.00024993	0.005563754
355	ASPM	0.058198699	23.739392	0.150184919	1.162049108	0.000272663	0.00605281
356	ERRFI1	0.083275615	16.80255154	0.150152663	1.162011625	0.000273431	0.006052912
357	ADRA1D	0.017415095	77.65887664	0.149888337	1.161704516	0.000279804	0.006176733
358	HLA-DPB1	0.20231605	7.320471049	0.148703362	1.160328741	0.000310102	0.006826559
359	MRM1	0.023915047	56.6742718	0.148670372	1.160290462	0.000310988	0.006827087
360	FAM131C	0.022035124	61.39036733	0.147710075	1.159176773	0.000337812	0.007395471
361	PLAU	0.061193863	22.55213962	0.147535188	1.158974066	0.000342922	0.007486657
362	NUDCD1	0.042133059	32.43067195	0.147358451	1.15876925	0.000348159	0.007580099
363	CNEP1R1	0.049965515	27.42567647	0.146210608	1.157439929	0.00038401	0.008337742
364	ECM2	0.038428779	35.41499647	0.14527153	1.156353512	0.000415846	0.00900432

Supplementary Table S4. Significantly enriched motifs found in HVGs.

Results were generated by GREAT (version 3.0.0) using hg19 human genome reference with association rule: Basal+extension: 5000 bp upstream, 1000 bp downstream, 1000000 bp max extension, curated regulatory domains included.

Cell type	# Term Name	Binom Rank	Binom Raw P-Value	Binom FDR Q-Val	Binom Fold Enrichment	Binom Obs. Region Hits	Binom Region Set Cov.
LCL	Motif NNNNKGGRAANTCCCN (no known TF)	1	3.35E-06	0.002058	2.685652	28	0.066038
LCL	Motif GGGAMTTYCC matches NFKB; RELA: v-rel reticuloendotheliosis viral oncogene homolog A, nuclear factor of kappa light polypeptide gene enhancer in B-cells 3, p65 (avian)	2	2.51E-05	0.007732	2.500147	26	0.061321
LCL	Motif NGGGACTTTCCA (no known TF)	3	7.05E-05	0.014461	2.346414	26	0.061321
LCL	Motif SRTGAGTCANC matches BACH2: BTB and CNC homology 1, basic leucine zipper transcription factor 2	5	0.000357	0.043963	2.236156	23	0.054245
LAEC	Motif STATAAAWRNNNNNN matches TAF<br>TATA	1	1.89E-05	0.011642	2.441259	28	0.06422
LAEC	Motif NNNNKGGRAANTCCCN (no known TF)	3	0.000107	0.022002	2.331906	25	0.057339
LAEC	Motif NGGGGAMTTTCCNN (no known TF)	4	0.000218	0.033477	2.1836	26	0.059633
LAEC	Motif GGGAMTTYCC matches NFKB; RELA: v-rel reticuloendotheliosis viral oncogene homolog A, nuclear factor of kappa light polypeptide gene enhancer in B-cells 3, p65 (avian)	5	0.000256	0.031449	2.24431	24	0.055046
LAEC	Motif TTGCWCAAY matches CEBPB: CCAAT/enhancer binding protein (C/EBP), beta	6	0.000471	0.048241	3.215633	12	0.027523

Supplementary Table S5. HVGs of induced pluripotent stem cell (iPSCs) (FDR<0.01)

ID	genes	u	cv2	residualcv2	fitratio	pval	fdr
1	TAGLN	1.358291551	5.316720491	1.245047724	3.473100546	2.86E-269	2.53E-265
2	LEFTY1	1.449540248	3.483336718	0.868531795	2.38340895	2.61E-114	1.15E-110
3	LINC00458	0.084785095	38.9765791	0.768449702	2.156420566	1.06E-86	3.12E-83
4	CSAG1	0.025124373	112.5062851	0.629075088	1.875874757	5.87E-56	1.30E-52
5	DHRS2	0.061374347	45.88767746	0.615139004	1.849913727	2.46E-53	4.34E-50
6	ACTC1	0.106228014	26.79126118	0.613042192	1.84603887	6.00E-53	8.83E-50
7	PTPN5	0.010049445	275.0743101	0.611091422	1.842441182	1.37E-52	1.73E-49
8	UBE2C	5.085267443	1.32896039	0.60791611	1.836600136	5.23E-52	5.77E-49
9	KPNA2	3.206429901	1.391359031	0.440113907	1.552884093	1.10E-26	1.08E-23
10	ANXA3	0.355106386	7.184182196	0.436654241	1.547520916	2.87E-26	2.54E-23
11	HIST1H4C	6.549518143	1.00178354	0.420596334	1.522869424	2.20E-24	1.77E-21
12	RP11-393M11.2	0.009718893	232.8120879	0.410930711	1.508220849	2.73E-23	2.01E-20
13	MT1G	0.617528499	4.28481023	0.40703696	1.502359632	7.36E-23	5.01E-20
14	LEFTY2	0.229302265	10.43600344	0.405975711	1.5007661	9.64E-23	6.08E-20
15	ADM	0.510891112	4.915200814	0.381067783	1.463846826	4.19E-20	2.47E-17
16	BBS9	0.254034066	9.014193821	0.355303548	1.426613635	1.40E-17	7.72E-15
17	ZBTB26	0.021019308	101.6929439	0.350797447	1.420199631	3.68E-17	1.91E-14
18	HIST1H2BK	0.932271558	2.885895107	0.349759093	1.418725726	4.59E-17	2.25E-14
19	ZNRF2	0.027444108	77.82998069	0.348244938	1.416579182	6.33E-17	2.94E-14
20	RP11-390B4.5	0.031742452	66.30285643	0.332231339	1.394075316	1.71E-15	7.55E-13
21	ID3	6.616024676	0.896206741	0.312726887	1.367148094	7.46E-14	3.14E-11
22	MYL9	0.71092063	3.446197116	0.307553036	1.360092941	1.95E-13	7.81E-11
23	MIR205HG	0.456579216	5.008210733	0.301108132	1.351355458	6.26E-13	2.40E-10
24	PKIB	0.890497935	2.847567571	0.300049754	1.34992597	7.56E-13	2.78E-10
25	STC1	0.05657726	36.20551005	0.298122856	1.347327304	1.07E-12	3.77E-10
26	RAB33B	0.031898313	63.36805048	0.291812008	1.338851301	3.22E-12	1.09E-09
27	H1F0	0.705648021	3.404457754	0.289180828	1.335333173	5.06E-12	1.66E-09
28	MT1H	0.186388053	11.28910528	0.28896598	1.33504631	5.25E-12	1.66E-09
29	TRDN	0.109527566	18.50747403	0.272810875	1.313651777	7.67E-11	2.34E-08
30	HIST1H3D	0.019917811	98.93258866	0.269765438	1.309657219	1.25E-10	3.67E-08
31	RP11-277P12.10	0.043369923	45.61356536	0.267012968	1.306057383	1.92E-10	5.49E-08
32	HIST1H2AC	0.658554839	3.523569842	0.265804971	1.304480623	2.33E-10	6.38E-08
33	RTTN	0.018073921	108.5204427	0.265647409	1.304275103	2.38E-10	6.38E-08
34	MEGF10	0.04095088	48.15885286	0.264605919	1.302917419	2.80E-10	7.28E-08
35	C19orf73	0.054664903	36.1849328	0.263709363	1.301749805	3.22E-10	8.13E-08
36	CDK1	1.105159503	2.313709115	0.260495829	1.297573302	5.28E-10	1.30E-07
37	HIST1H1D	0.320603237	6.605609406	0.259506513	1.296290227	6.14E-10	1.47E-07
38	FAM149A	0.042287502	46.2284187	0.255434844	1.291022893	1.14E-09	2.64E-07
39	RP11-509E16.1	0.037459502	52.04248952	0.2540395	1.289222727	1.40E-09	3.17E-07
40	RAB40B	0.035136909	55.27744009	0.250995366	1.285304128	2.19E-09	4.84E-07

Supplementary Table S5. HVGs of induced pluripotent stem cell (iPSCs) (FDR<0.01)

41	LOH12CR2	0.015353552	125.4799757	0.248508871	1.282112196	3.15E-09	6.79E-07
42	PIANP	0.052738739	36.7410168	0.243633162	1.275876205	6.35E-09	1.34E-06
43	GAS7	0.021426679	89.14497962	0.238182704	1.268941012	1.37E-08	2.81E-06
44	TOP2A	0.937896996	2.5569175	0.233469416	1.262974201	2.61E-08	5.24E-06
45	CBR3	0.48845566	4.406397665	0.232516721	1.261771545	2.97E-08	5.83E-06
46	PYY	0.019033068	99.56366965	0.230940122	1.259783804	3.67E-08	7.05E-06
47	NPHP3	0.05198402	36.78872646	0.230730365	1.259519583	3.78E-08	7.10E-06
48	PDLIM3	0.064477328	29.74121303	0.229923	1.258503101	4.21E-08	7.75E-06
49	AMER1	0.077281659	24.61017297	0.218090273	1.243699336	1.96E-07	3.54E-05
50	RP11-152N13.5	0.062086252	30.42922975	0.215677898	1.240702682	2.66E-07	4.69E-05
51	NLGN2	0.118080454	16.19799415	0.212336345	1.236563727	4.02E-07	6.97E-05
52	AC144652.1	0.034723198	53.78102082	0.21182458	1.23593106	4.28E-07	7.28E-05
53	PHLDA3	0.356382971	5.688520749	0.206478815	1.22934169	8.18E-07	1.36E-04
54	SCARA3	0.138605217	13.79482015	0.206323803	1.229151142	8.34E-07	1.36E-04
55	RP11-563N4.1	0.084197692	22.31960343	0.204166493	1.226502341	1.08E-06	1.73E-04
56	DDHD1	0.074228873	25.15644649	0.200598692	1.222134221	1.64E-06	2.58E-04
57	HIST1H2BD	0.170575644	11.24073375	0.20033741	1.221814941	1.69E-06	2.61E-04
58	LRRN2	0.0515808	35.95400351	0.200106545	1.2215329	1.73E-06	2.64E-04
59	SPP1	1.172423656	2.078221922	0.19768931	1.218583733	2.29E-06	3.42E-04
60	SOCS1	0.785849162	2.842336206	0.197408123	1.218241131	2.36E-06	3.48E-04
61	CA8	0.085442136	21.79835054	0.194858606	1.215139161	3.15E-06	4.57E-04
62	HEXIM1	0.119661433	15.67568642	0.192421161	1.212180933	4.14E-06	5.90E-04
63	RECQL5	0.050193579	36.6431141	0.192221665	1.211939131	4.24E-06	5.94E-04
64	DIAPH2	0.805863445	2.765084983	0.190326236	1.209644163	5.22E-06	7.21E-04
65	SPATA5L1	0.065481422	28.1465296	0.189983226	1.209229314	5.42E-06	7.37E-04
66	RBBP8NL	0.021450769	84.12688105	0.18136163	1.19884864	1.37E-05	1.83E-03
67	CHKB-AS1	0.048275848	37.57218191	0.178847104	1.195837891	1.78E-05	2.34E-03
68	MT1E	0.348290947	5.642531497	0.177502954	1.194231585	2.04E-05	2.65E-03
69	TCEAL7	0.07176915	25.37528562	0.176253232	1.19274006	2.32E-05	2.97E-03
70	SNURF	0.031051185	57.830128	0.173686899	1.189683017	3.01E-05	3.80E-03
71	MED14-AS1	0.031294699	57.35577271	0.173192992	1.189095569	3.16E-05	3.93E-03
72	ARHGAP22	0.056162387	32.15464327	0.172225705	1.187945928	3.48E-05	4.27E-03
73	CTGF	0.175639565	10.59863384	0.169388837	1.184580658	4.61E-05	5.53E-03
74	ZNF329	0.044877329	39.99652234	0.169339629	1.184522368	4.63E-05	5.53E-03
75	FGF8	0.56474581	3.638032498	0.167006696	1.181762178	5.81E-05	6.84E-03
76	EVC2	0.027584883	64.58996816	0.166853679	1.181581362	5.90E-05	6.85E-03
77	CTA-29F11.1	0.408061265	4.834980168	0.165950288	1.180514414	6.43E-05	7.38E-03
78	EME1	0.057503737	31.14927497	0.163683664	1.177841663	7.98E-05	9.04E-03
79	TBC1D31	0.085827082	21.03369336	0.163537279	1.177669257	8.09E-05	9.05E-03
